# Antibiotic treatment of acute and recurrent otitis media in children: an Italian intersociety Consensus

**DOI:** 10.1186/s13052-025-01894-z

**Published:** 2025-02-20

**Authors:** Guido Castelli Gattinara, Marcello Bergamini, Giovanni Simeone, Lamberto Reggiani, Mattia Doria, Daniele G. Ghiglioni, Alberto Terminiello, Federica Cosentino, Laura Cursi, Daniele Donà, Elena Chiappini, Luisa Galli, Andrea Lo Vecchio, Alfredo Guarino, Alberto Villani, Giuseppe Di Mauro, Nicola Principi, Susanna M. R. Esposito, Maria Carmen Verga

**Affiliations:** 1 Institute of Child Health, Bambino Gesù Children’s Hospital, IRCCS, Piazza S. Onofrio 4, 00165 Rome, Italy; 2 Pediatrician, Indipendent Researcher, AUSL Ferrara, 44121 Ferrara, FE Italy; 3 Family Pediatrician, Local Health Unit Brindisi, Mesagne, Brindisi, Italy; 4 Family Pediatrician, Local Health Unit, Imola, Italy; 5 Primary Care Paediatrician, Local Social and Health Unit “Serenissima”, National Health Service, Venice, Italy; 6 Fondazione IRCCS Ca’ Granda Ospedale Maggiore Policlinico, Milan, Italy; 7 Department of Health Sciences, Meyer University Hospital, University of Florence, Florence, Italy; 8 Unit of Infectious Diseases, ARNAS Garibaldi Hospital, University of Catania, Catania, Italy; 9 Department of Pediatrics, Bambino Gesù Children’s Hospital, IRCCS, Rome, Italy; 10 Division of Pediatric Infectious Diseases, Department for Women’s and Children’s Health, University of Padua, Padua, Italy; 11 Department of Translational Medical Sciences, Federico II University, Naples, Italy; 12 Department of Pediatrics, Bambino Gesù Children’s Hospital, IRCCS and University of Rome Tor Vergata, Rome, Italy; 13 Pediatric Primary Care, National Pediatric Health Care System, Caserta, Italy; 14 Università Degli Studi Di Milano, 20122 Milan, Italy; 15 Department of Medicine, Pediatric Clinic, Pietro Barilla Children’s Hospital, University Hospital of Parma, Parma, Italy; 16 Family Pediatrician, Local Health Unit Salerno, Vietri Sul Mare, Salerno, Italy

**Keywords:** Acute otitis media, Recurrent otitis media, Tympanic membrane perforation, Antibiotic therapy, Children primary care

## Abstract

Acute Otitis Media (AOM) typically affects previously healthy children and can be recurrent. This inter-society consensus aims to provide evidence-based recommendations for the antibiotic therapy of mild, severe and recurrent otitis media in previously healthy children in Italy.

A systematic literature review was conducted to identify the most recent/relevant evidence. The Embase, Scopus, PubMed, and Cochrane databases were used with the terms "children," "acute otitis media”, “recurrent otitis media”, and "antibiotics," from 2012 to April 2024, with no language restrictions. The review focused on studies conducted in high-income countries involving antibiotic therapy in children over 3 months of age diagnosed with AOM or Recurrent AOM (RAOM). The GRADE ADOLOPMENT was used to assess the possibility of adopting or adapting recommendations from two evidence-based guidelines: 'NICE guideline Otitis media (acute): antimicrobial prescribing', updated to 2022 and SIP Intersocietal GL 2019 “Management of acute otitis media in paediatric age: diagnosis, therapy and prevention” The certainty of the evidence was assessed using the GRADE approach. Final recommendations were formulated through a Delphi consensus process with an expert panel.

All major randomised trials and international guidelines promote the appropriate use of antibiotics and advocate a therapy with narrow-spectrum molecules (amoxicillin). The amoxicillin-clavulanic acid is only envisaged when there is a risk of infection by β-lactamase-producing bacteria. In healthy children, amoxicillin should be initiated as a first-line treatment only after a 48–72-h period of appropriate "watchful waiting", during which symptoms are treated while monitoring the patient clinically to see if symptoms resolve. Amoxicillin-clavulanate or second-generation cephalosporins should be reserved for non-immunized children, those with immune deficiencies or those with underlying conditions. In these cases, the use of a clavulanic acid-protected amoxicillin is preferred even though there is no specific scientific evidence to support this choice.

The recommended amoxicillin dosage is 90 mg/kg/day, divided into three doses, though two doses may be considered to improve compliance. A five-day duration of therapy is advised.

In conclusion the diagnosis of AOM/RAOM relies primarily on clinical assessment, which often introduces uncertainty in distinguishing between viral and bacterial infections whereby there remains significant potential to improve antibiotic utilisation. Future studies could play a key role in enhancing the management of AOM/RAOM in children, ensuring that antibiotic treatments are appropriate and effective.

**Supplementary Information:**

The online version contains supplementary material available at 10.1186/s13052-025-01894-z.

## Background

Acute otitis media (AOM) is an upper respiratory tract infection and one of the most prevalent childhood illnesses. Over 60% of children under 3 years old have at least one episode, and about 24% have at least three episodes [1, 2]. AOM remains a leading reason for antibiotic prescriptions in children, accounting for up to 40% of all prescriptions in some cases [3, 4]. Considering that the Italian Medicines Agency has reported a rise in antibiotic consumption, campaigns and guidelines for their judicious use are very important [5]. Moreover, in a European comparison, Italy shows a greater use of broad-spectrum antibiotics, which have a higher impact on the development of antibiotic resistance. Italy is also among the countries with the lowest proportion of antibiotics in the "Access" group (47%), which are considered the first-choice antibiotics and should, according to WHO recommendations, make up at least 60% of total consumption [6]. In the AIFA document, significant regional variability in consumption is noted, which cannot be explained epidemiologically but rather reflects inappropriate prescribing and consumption practices, highlighting substantial room for improvement in prescriptive appropriateness. In the paediatric population, the most significant consumption is concentrated in the age group between 2 and 5 years, in which about 4 out of 10 children receive at least one antibiotic prescription annually. Ensuring the appropriate use of antibiotics in children with AOM is therefore crucial. The development of guidelines for AOM treatment has been shown to reduce inappropriate antibiotic prescriptions by up to 20% [4, 7]. In Italy, national guidelines were published in 2019 [8], followed by updates from the NICE group in March 2022 [9]. This document aims to update treatment strategies for AOM and Recurrent Acute Otitis Media (RAOM) by stating appropriate antibiotic stewardship, considering the high prevalence of these conditions, and addressing the ongoing changes in pathogen epidemiology and antibiotic resistance.

*Diagnosis*: AOM is an inflammation of the middle ear characterised by three key diagnostic elements [8–10]:

1) Acute onset of symptoms:

- Otalgia (in older children, this can manifest as pulling, rubbing or striking the ear)

- Non-specific symptoms, which include fever, irritability, crying, poor feeding, nocturnal restlessness, cough or rhinorrhea, particularly in younger children

plus

2) Signs of inflammation on examination:

- A marked hyperemic (red), yellowish, and/or opaque eardrum

plus

3) Presence of endo tympanic exudate, indicated by:

- moderate to severe swelling of the tympanic membrane with loss of typical landmarks

or

- impaired compliance of the tympanic membrane (as determined by pneumatic otoscope or impedance measurement)

or

- Perforation of the tympanic membrane with serum effusion or exudate in the external auditory canal.

Recurrent AOM is defined by Goycoolea [11] as at least three distinct episodes of acute otitis media within 6 months or four or more episodes within 12 months. Major risk factors for recurrent otitis media include allergies, upper respiratory infections, chronic nasal obstruction and snoring.

AOM must be differentiated from Exudative Otitis Media (EOM), which involves the presence of exudate in the middle ear without symptoms or signs of infection.

Diagnosing AOM in children younger than 6 months can be difficult due to non-specific symptoms or concurrent systemic illnesses, such as bronchiolitis. Misdiagnoses, especially confusing EOM or myringitis (a nonspecific infection causing tympanic membrane hyperemia) with AOM, can lead to inappropriate antibiotic use.

The pneumatic otoscope (and/or tympanometer) is the optimal diagnostic tool for reducing diagnostic uncertainty. However, these tools remain underused in Italian clinical practice, and their increased use in pediatric settings is recommended.

*Defining the degree of severity*: the AOM severity significantly influences treatment decisions. However, international studies and guidelines often lack consistent definitions of severity. The 2019 Italian guidelines recommend that diagnosis and severity be based on the simultaneous presence of clinical symptoms and otoscopic signs. An Italian severity score was developed to standardise the severity assessment [8], considering both symptoms and signs. This scoring system is straightforward and practical (Table 1).

**Table 1:** Definition of severity: Severe AOM with an overall score of 4 or more [8]

**Axillary body temperature**	
38,0–38,9 °C	1
≥ 39,0 °C	2
**Compromised general condition**	
Absent	0
Present	4
**Otalgia**	
Mild/moderate	0
Intense and/or inconsolable crying	2
**Tympanic membrane hyperemia**	
Mild/moderate	0
Bilateral	2
Intense	2
**Tympanic membrane eversion**	
Mild/moderate	1
Marked	4

AOM can be caused by both viruses and bacteria, often coexisting. Distinguishing between viral and bacterial infections based solely on clinical presentation is challenging. Children with viral infections or low-virulence bacterial pathogens often experience spontaneous resolution of AOM, with 60% showing symptom improvement within 24 h, and over 80% recovering within 3 days [12, 13]. Conversely, a child with progressively worsening symptoms is more likely to have a bacterial infection requiring treatment. Clinical indicators of bacterial infection include [14]:

- convex or everted tympanic membrane

- a perforated tympanic membrane with discharge of purulent exudate

which are correlated, in fact, with increased severity of pathology.

The most common bacterial pathogens are *Streptococcus pneumoniae, Haemophilus influenzae and parainfluenzae, Moraxella catarrhalis and Streptococcus pyogenes*. In more severe AOM with tympanic membrane perforation *Streptococcus pneumoniae* is still the most frequent pathogen (26%) followed by *Haemophilus influenzae* (18.8%) and *Staphylococcus aureus* (12.3%) [15, 16].

AOM is essentially a self-limiting condition, with complications being rare even without immediate antibiotic use. This has led to recommendations for a "watchful waiting" approach in selected cases, observing the child with AOM for the first 48–72 h, providing symptomatic treatment only, and re-assessing the patient if needed [8, 16]. The benefits of this approach include reduced side effects, lower risk of antibiotic-resistant bacterial strains, and decreased healthcare costs.

The *American Academy of Pediatrics* (AAP) [10] and other guidelines [17, 18] recommend immediate antibiotic therapy only for bilateral AOM, AOM associated with otorrhea, or in children under 2 years of age, who typically experience more severe and prolonged infections. In other cases, AOM often resolves without antibiotics. Antibiotics or further investigations are advised only for patients with severe symptoms or at high risk of complications from comorbid conditions such as cardiac, pulmonary, renal, hepatic, or neuromuscular disease, immunosuppression, cystic fibrosis and prematurity.

### Antibiotic therapy

International guidelines recommend using narrow-spectrum molecules, such as high-dose amoxicillin, to ensure the appropriate use of antibiotics. The amoxicillin-clavulanic acid is only recommended when there is a risk of infection by β-lactamase-producing microorganisms. This strategy provides adequate coverage for most bacteria involved in AOM, even in immunocompromised children where the risk of therapeutic failure must absolutely be avoided. However, comprehensive epidemiological data on AOM in Italy remain lacking.

Pain management is crucial for the therapeutic success of the “watchful waiting” strategy. Systemic painkillers, like paracetamol and ibuprofen [8] can be used to relieve the pain and fever of AOM. Additionally, topical analgesic ear drops may help control ear pain [19, 20].

### Complications of AOM

Severe systemic infections or acute complications, such as mastoiditis, meningitis, intracranial abscess, sinus thrombosis and facial nerve palsy, are rare and generally require hospitalization [21, 22]. The risk of mastoiditis after AOM is 1.8 per 10,000 episodes with antibiotic use, compared to 3.8 per 10,000 without antibiotics. To prevent one case of mastoiditis, 4,831 patients would need to be treated with antibiotics. Other common complications include recurrence of the infection and tympanic membrane perforation (rupture of the eardrum), which occurs in about 2% of AOM cases [23]. Certain clinical conditions, such as congenital immune deficits, immunosuppressive therapy, craniofacial and ear malformations, genetic diseases and disabilities, may predispose patients to complications.

## Materials and Methods

This document refers to the outpatient and emergency room setting of Family Paediatrics and General Practitioners, and hospitalised patients. It provides recommendations for children aged one month to 18 years with acute otitis media (OMA) and recurrent acute otitis media (OMAR).

The questions and outcomes were identified, shared and discussed by the panel. Questions were formulated using the "PICO" model (Patient/Population [P]; Intervention/Indicator [I]; Comparator/Control [C]; Outcome [O]) and developed following the GRADE method.

The panel identified outcomes a priori, classified them and voted (from 1 to 9) based on their importance in the decision-making process. Only those classified as "critical" and "important" were considered in the systematic review and subsequent formulation and grading of the recommendations.

The methodology of the document is detailed in the Supplementary file S8. “Italian Intersociety Consensus (SIPPS-SIP-SITIP-FIMP-SIAIP-SIMRI-FIMMG) on antibiotic therapy of respiratory infections in pediatric age. Structure and Methodology of the Document”.

Critical outcomes considered in this analysis include:

- symptom reduction (duration or severity), e.g. time to significant improvement.
- time to clinical recovery (mean or median time to disease resolution).
- complication rates (including mortality) with or without treatment, including treatment escalation.
- health and social care utilisation (planned and unplanned contacts).
- thresholds or indications for antimicrobial treatment (which people are more or less likely to benefit from antimicrobials).

Important outcomes include:

- patient-reported outcomes, such as medication adherence.
- changes in patterns, trends and levels of antimicrobial resistance following treatment.

To address the questions posed by the consensus panel:

- Guidelines (GLs) published in the last 5 years were reviewed.
- the GRADE ADOLOPMENT method was used (SNL Methodological manual for the production of clinical practice guidelines) [24] to assess the possibility of adopting or adapting recommendations from two evidence-based guidelines: the 2018 NICE (National Institute for Health and Care Excellence) GLs 'NICE guideline [NG91] Otitis media (acute): antimicrobial prescribing', updated to 2022 [19] and SIP (Società Italiana di Pediatria) Intersocietal GL 2019 “Management of acute otitis media in paediatric age: diagnosis, therapy and prevention” [8], with possible additions/amendments based on updates and assessments of the Italian outpatient setting. The most important questions answered by the 2 GLs are superimposable, to a certain extent, with those envisaged by the Consensus.

An update of the NICE GLs (Update 2022) has recently been published, but it only updates some recommendations not relevant to antibiotic therapy.

In practice, the systematic search of the 2018 NICE GLs ends in December 2016, while that of the SIP LGs 2019 ends on 31.12.2018.

A systematic review was conducted from December 2016 to June 2022 on PubMed, EMBASE, and relevant governmental and scientific society websites.

For questions on antibiotic prophylaxis of RAOMs (question 7) and topical antibiotic therapy of AOMs with spontaneous perforation otorrhea (question 19), a systematic review of the original evidence was performed due to limited recommendations in the 2018 NICE guidelines and the 2019 SIP guidelines’ reliance on a single search engine. Systematic reviews published within the last 10 years weresearched and assessed, and if none were found, original studies were reviewed. 

The inclusion criteria were:

- type of study: systematic reviews with and without meta-analysis, randomised and non-randomised controlled trials.
- conducted on the population of interest,
- with correct diagnostic criteria of AOM and/or RAOM,
- with a minimum follow-up period of at least 3 months to assess the recurrence of AOM.

The excluded criteria were:

- non-controlled studies,
- conducted in settings and on populations other than those specified in the questions (e.g. children with comorbidities or adults, hospitalised patients),
- with interventions and outcomes not relevant to the questions,
- of very low methodological quality.

Refer to Supplementary files 1–7 for the detailed search strategy, quality assessment, study results, and GRADE evaluation.

This article is part of the Italian Inter-society Consensus on the judicious use of antibiotic therapy in respiratory tract infections in childhood (including pharyngitis, sinusitis, and community pneumonia, published separately). It involves the Italian Society of Preventive and Social Pediatrics (SIPPS), the Italian Society of Pediatrics (SIP), the Italian Society of Pediatric Infectious Diseases (SITIP), the Italian Federation of Pediatricians (FIMP), the Italian Society of Pediatric Allergy and Immunology (SIAIP), the Italian Society of Pediatric Respiratory Diseases (SIMRI), the Italian Federation of General Practitioners (FIMMG), and the Italian Society of General Medicine and Primary Care (SIMG).

### Questions and recommendation on acute and recurrent acute otitis media

#### Question 1. Does the age of the child influence the treatment strategy for AOM?

Acute otitis media is an infection of the middle ear caused by viruses or bacteria, often both, and it typically resolves without treatment. This has led to the recommendation of an observation period or 'watchful waiting' before initiating antibiotic treatment in some patients [8, 16].

Watchful waiting involves observing the clinical course of a child with AOM during the first 48–72 h, treating only the symptoms with pain relief, and reassessing the patient after this period. The anticipated benefits of avoiding immediate antibiotic use include reduced side effects, decreased selection of antibiotic-resistant bacterial strains, and lower healthcare costs.

A delayed prescription is another option, where a 'post-dated' prescription is provided but only dispensed if symptoms worsen after a few days. According to the latest NICE guidelines [2018] [25], immediate antibiotic therapy is recommended only in more severe cases, such as children under 2 years old with bilateral AOM (Number needed to treat—NNT = 4 for symptom resolution) and in children with AOM accompanied by tympanic perforation otorrhea, where the NNT for symptom resolution is 3 [26]. A meta-analysis of six Randomized Control Trials (RCTs) [26] indicates that antibiotic treatment is more effective for bilateral AOM, but not statistically significant for unilateral AOM. The 2019 SIP guidelines [8] also suggest limiting antibiotic intervention in children over 2 years old unless the AOM presents severe symptoms. An analysis of international guidelines shows consensus on recommending watchful waiting in children over two years old, with less agreement for those between six and 24 months [27]. Several observational studies support the wait-and-see approach to antibiotic therapy [28, 29]. The 2013 AAP guidelines [25] also recommended watchful waiting in children aged six to 24 months with mild unilateral AOM, in contrast to earlier guidelines. This recommendation is based on a meta-analysis of individual data from studies included in the AAP guidelines [26]. A double-blind clinical trial [30] on 291 children aged 6–24 months with AOM, comparing immediate treatment with amoxicillin-clavulanic acid (90 mg/kg/day) to placebo, shows a greater resolution of symptoms in children who received the antibiotic (*P* = 0.04). However, the percentage of persistence of signs of acute otitis at otoscopic evaluation was higher in the placebo group (23% vs. 4% *P* < 0.001) on day 4 and day 10 (51% vs. 16% *P* < 0.001 NNT = 2.9). The authors conclude that treatment of AOM with an antimicrobial agent is beneficial for children between 6 and 24 months, even though a more careful analysis of the results shows that this therapy is only helpful in about 20% of cases with persistent signs of AOM at otoscopy.

An extensive review of 13 RCTs involving 3400 children [1] shows that antibiotics have a modest effect on reducing tympanic perforations, contralateral otitis episodes and abnormal tympanometric findings at 2–4 weeks and 6–8 weeks compared to placebo. However, antibiotics come with risks: for every 14 children treated with antibiotics, one experienced an adverse event such as vomiting, diarrhoea, or rash, that would not have occurred without antibiotics. The reduction in the number of children with perforation of the eardrum in this study appears relative: 5% vs. 2% of children had a perforation (NNT 33 [range 20–100]; moderate quality evidence). Antibiotics were most beneficial in children under two years old with bilateral AOM or AOM with otorrhea.

In conclusion, based on the literature, there is no increased risk of complications when antibiotic therapy is not administered immediately, even in children under two years old. Watchful waiting up to 72 h after diagnosis may be considered in all forms of otitis that do not present severe symptoms (such as perforation or bilaterality) and where follow-up visits or telephone contact with parents are feasible.

### Recommendation 1

For children older than 6 months with AOM: A 'watchful waiting' treatment strategy, where antibiotics are not prescribed immediately (or prescribed with a delay), should be considered and agreed upon with parents to verify the actual need for antimicrobial therapy *(Low-moderate quality evidence. Strong recommendation in favour of the intervention).*

- 1.1 For infants younger than 6 months with AOM: immediate antibiotic therapy is recommended due to a lack of safety and efficacy data on the watchful waiting treatment strategy in this age group *(Expert opinion. Strong recommendation in favour of the intervention).*
- 1.2 For children aged 6–24 months with bilateral AOM: immediate prescription of antibiotics is recommended, irrespective of severity and except point 1 *(Low-quality evidence. Weak recommendation in favour of intervention).*
- 1.3 For children aged 6–24 months with mild unilateral AOM: A watchful waiting strategy should be recommended. *(Moderate-quality evidence. Weak recommendation in favour of intervention)*
- 1.4 For children older than 24 months and adolescents with unilateral or bilateral AOM: A watchful waiting strategy is recommended in all cases without comorbidities or risk factors, and considering specific severity conditions requiring immediate treatment. *(Moderate-quality evidence. Strong recommendation in favour of intervention).*
- 1.5 For children older than 6 months where watchful waiting is indicated, this strategy is only recommended if follow-up within 72 h is possible and comorbidities are absent. *(Expert opinion. Strong recommendation in favour of this approach).*

#### Question 2. Does the severity of the AOM episode influence the timing of antibiotic therapy?

The 2018 NICE guidelines recommend that an immediate antibiotic prescription be offered to all children and young people with severe systemic symptoms, serious symptoms or signs of AOM, or those at high risk of serious complications due to pre-existing comorbidity (such as significant cardiac, pulmonary, renal, hepatic or neuromuscular disease, cystic fibrosis, prematurity, immunodeficiency or on immunosuppressive therapies). They also recommend referring children and adolescents with AOM associated with severe systemic infection or acute complications (e.g. mastoiditis, meningitis, intracranial abscesses, sinus thrombosis or facial nerve palsy) to a hospital. However, the guidelines do not explicitly define what constitutes severe conditions. The benefits of immediate antibiotic treatment in cases of AOM with otorrhea are highlighted in systematic reviews by Rovers et al. [26] and Shekelle et al. [31], while treatment for severe bilateral otitis media is supported by evidence from RCTs by Tahtinen et al. [30] and Hoberman et al. [32]. The Italian Society of Paediatrics (SIP) also recommends immediate antibiotic treatment for children with severe AOM or systemic disease. The 2019 SIP guidelines define “severe AOM” using a clinical scoring system, considering AOM severe if the overall score is 4 or higher (Table 1). The 2013 guidelines also support treatment for children with a compromised appearance, persistent otalgia for more than 24 h, or a temperature of 39°C in the past 48 h, and they emphasise the importance of ensuring reliable follow-up care. In contrast, no specific evidence support the need for immediate treatment for severe but unilateral AOM, as noted in the 2019 SIP guidelines and a systematic review by Venekamp et al. [1], along with 4 different randomised clinical trials [19]. The rationale is that watchful waiting, especially in older children, does not lead to an increased risk of serious complications, such as mastoiditis and abscesses, or recurrence of infections.

### Recommendations 2

#### 2.1 In children and adolescents with AOM, immediate antibiotic therapy is recommended in the following severe conditions

- AOM with otorrhea *(Moderate quality evidence. Weak recommendation for intervention).*

- severe bilateral AOM *(Moderate quality evidence. Strong recommendation for intervention).*

- AOM with systemic symptoms and impaired general status *(Moderate quality evidence. Strong recommendation in favour of intervention).*

#### 2.2 In children older than 24 months and adolescents with AOM, considering the low risk of complications, the "watchful waiting" strategy should be recommended in the following conditions

- severe unilateral AOM without systemic symptoms, impaired general status, or otorrhea *(Expert opinion. Weak recommendation in favour of intervention).*

- unilateral or bilateral AOM without signs of severity *(Moderate quality of evidence. Strong recommendation in favour of intervention).*

#### Question 3. When does antibiotic therapy become necessary

3.1 can amoxicillin still be considered the first-choice antibiotic for children with AOM?

The NICE guidelines committee, recognising that AOM is a non-life-threatening infection, recommends that if an antibiotic is needed, a narrow-spectrum antibiotic should be the first choice. The excessive use of broad-spectrum antibiotics can lead to the selection of resistant bacteria and disrupt the normal flora, increasing the risk of infections with harmful, antibiotic-resistant organisms like *Clostridium difficile*. Broad-spectrum antibiotics should therefore be reserved for cases where narrow-spectrum antibiotics have proven ineffective [19].

Based on available evidence, the NICE committee found no significant difference in clinical efficacy among different antibiotic classes. As such, they recommend that the choice of antibiotic should be guided by minimising the risk of resistance. The NICE committee and SIP guidelines suggest that amoxicillin remains the first-choice antibiotic for treating AOM in children, as it is commonly used and has an acceptable resistance profile. Thus, the NICE committee and the SIP GLs recommend amoxicillin as the first choice, because it aligns with current practice for treating AOM and poses an acceptable risk of resistance.

### Recommendations 3.1

For children and adolescents with AOM requiring antibiotic therapy, amoxicillin is recommended as the first-choice antibiotic. *(Moderate quality evidence for penicillin vs. cephalosporins, low for penicillin vs. macrolide. Strong recommendation in favour of intervention).*

3.2 is amoxicillin at 80–90 mg/kg/day more effective than 50mg/kg/day?

Increasing the amoxicillin dosage from 40–50 mg/kg to 80–90 mg/kg per day enhances its concentration in the middle ear [33], which improves the efficacy against most strains of *Streptococcus pneumoniae*, including those with intermediate resistance (Minimum Inhibitory Concentration—MIC ≥ 2 and < 8 mcg /mL) [34]. Only the highly resistant *Streptococcus pneumoniae* strains (MIC ≥ 8 μg /mL), which represent less than 2% of isolates, do not respond to high doses of amoxicillin [35]. Therefore, significant guidelines. including NICE, SIP and AAP, recommend high amoxicillin doses (80–90 mg/kg/day). This recommendation is supported by other guidelines (Belgium, France, South Africa, etc.) and is aligned with Italy’s antibiotic resistance data, where penicillin resistance in *Streptococcus pneumoniae* is 8% (Emilia Romagna data 2016) [17].

### Recommendations 3.2

For cases of uncomplicated AOM with mild symptoms in children without risk factors for bacterial resistance or a history of recurrence, amoxicillin should be prescribed, when necessary, at 80–90 mg/kg/day *(Low quality of evidence. Expert opinion. Strong recommendation in favour of intervention).*

3.3 Should amoxicillin (or amoxicillin/clavulanate) be administered in 2 or 3 daily doses?

The NICE guidelines (2018) [19] reference a systematic review and meta-analysis of RCTs [36] that found no significant differences in clinical cure rates between administering amoxicillin or amoxicillin-clavulanic acid twice daily versus three times daily over a 10-day course (high-quality evidence). Additionally, no significant differences were found in infection recurrence rates, adverse effects (low-quality evidence) or treatment adherence (high-quality evidence). Despite this, the 2018 NICE committee [19] agreed that a three-times daily schedule should be recommended. The SIP working group [8] also strongly supports the three daily administrations of amoxicillin-clavulanic acid (strong positive recommendation). Therefore, in children with AOM, a twice daily schedule for amoxicillin or amoxicillin-clavulanic acid is not recommended. While there is no clear evidence that two-dose administration increased the risk of treatment failure, suppurative complications or short-term relapse, it does not appear to enhance adherence to therapy [19]. However, in particular conditions where administration in three doses creates great difficulties for the family, administration in two doses may be considered, especially if it facilitates better adherence.

### Recommendations 3.3

In children and adolescents with AOM, it is recommended to divide the daily dose of amoxicillin (or amoxicillin/clavulanate) into three administrations. *(Low quality of evidence. Expert opinion. Weak recommendation in favour of intervention).*

#### Question 4. Is a 5, 7, or 10-day duration of therapy more effective for treating AOM in children and adolescents?

Based on evidence, panel experience and antibiotic resistance data, the NICE guidelines suggest that a 5–7-day course of any recommended antibiotic is sufficient to treat AOM in children. While some studies have involved longer durations, no direct comparisons between 5-day and 7-day courses have been identified. However, evidence indicates that a shorter course of fewer than 7 days may lead to a higher risk of therapeutic failure than a course of 7 days or more, although the difference is small. As a result, the NICE committee agreed that a 5-day course might be adequate for many children, with 7-day courses reserved for those with more severe or recurrent infections.

Similarly, the SIP guidelines recommend extending amoxicillin or amoxicillin-clavulanic acid therapy to 10 days in children at risk of unfavourable outcomes (e.g., under 2 years of age or with spontaneous otorrhea). A shorter 5-day course is weakly recommended for children without such risk factors (e.g., older than 2 years, without otorrhea, bilaterality, or severe symptoms). This recommendation is supported by a 2010 Cochrane review, which found that antibiotic courses shorter than 7 days were associated with a higher risk of failure concerning those longer than 7 days (21% vs. 18%) [37].

In children under two years of age, a high-quality randomised clinical trial by Hoberman et al. [38] compared the efficacy of amoxicillin-clavulanic acid therapy for 10 days versus a 5-day schedule in 520 children. Children treated with the antibiotic for 5 days had a higher risk of clinical alliment than those treated for 10 days (34% vs. 16%; *P* = 0.02); this difference increased further in those with bilateral AOM (*P* < 0.001). Clinically, the percentage of children with symptom reduction was lower in the group treated with a short course than in those treated for 10 days (80% vs. 91%; *P* = 0.003), although the clinical significance of the result is modest.

### Recommendations 4

4.1. For children requiring antibiotic therapy, amoxicillin or amoxicillin-clavulanate should be administered for 7 or 10 days in high-risk or severe conditions. *(High quality of evidence. Strong recommendation in favour of intervention).*

4.2 For children without risk factors for unfavourable outcomes (e.g. older than 2 years, without otorrhea, without recurrence, without bilaterality, without severe symptoms, after a period of watchful waiting), a shorter course of 5 days may be recommended*. (Very low quality of evidence. Weak recommendation in favour of intervention).*

#### Question 5. Which antibiotic is recommended for children with AOM who do not recover or experience short-term relapse after therapy?

Both the NICE and SIP guidelines recommend amoxicillin-clavulanic acid (80–90 mg/kg/day in 3 doses) as the second-choice antibiotic for children whose symptoms worsen after 2–3 days of first-line antibiotic therapy, based on evidence, experience and data on antibiotic resistance [6, 17, 29, 37]. This broad-spectrum treatment combines a penicillin (amoxicillin) with a β-lactamase inhibitor, making it active against β-lactamase producing bacteria that are resistant to amoxicillin alone.

When using this combination, it is necessary to consider that.

- 1. amoxicillin should be administered at a dose of 80-90 mg/kg/day,
- 2. the standard ratio of amoxicillin to clavulanic acid in Italian formulations is 7:1,
- 3. at the recommended amoxicillin dose (80-90 mg/kg/day), the clavulanic acid dose is slightly higher than the maximum recommended (11.7 mg/kg/day vs. 10 mg/kg/day)
- 4. clavulanic acid is primarily responsible for the most common adverse events, such as diarrhoea,
- 5. Lower doses of clavulanic acid reduce the incidence of diarrhoea without compromising clinical efficacy.

To improve the tolerability profile of amoxicillin-clavulanic acid, one should therefore, if possible, maintain the standard dose of the amoxicillin-clavulanic acid formulation (amoxicillin dose of 40 mg/kg/day: 0.5 ml/kg/day divided into 3 administrations), supplementing with a plain amoxicillin formulation (50 mg/kg/day: 1 ml/kg/day divided into 3 doses) to reach the desired dose of amoxicillin (80/90mg/kg/day) and clavulanic acid (< 10 mg/Kg/day). If supplementation is not possible, the full dose of amoxicillin is administered only as a second choice with the amoxicillin-clavulanic acid combination.

### Recommendations 5

In children and adolescents with AOM who do not recover or experience short-term relapse (within 30 days), or whose symptoms worsen after at least 2–3 days of amoxicillin therapy at a dose of 80/90mg/kg/day it is recommended to use the combination amoxicillin-clavulanate at a dose of 40 mg/kg/day of amoxicillin (0.5ml/kg/day) in 3 administrations, supplemented with a plain amoxicillin formulation (50 mg/kg/day or 1 ml/kg in 3 administrations) to ensure optimal doses of amoxicillin and clavulanic acid (< 10 mg/Kg/day). *(Moderate quality of evidence. Strong recommendation in favour of intervention).*

#### Question 6. What is the first-choice antibiotic therapy for an acute episode in children with RAOM?

The question of what constitutes the first-choice therapy for RAOM in children is primarily based on the 2010 NICE systematic review [31], as the updated review did not provide any new findings. The treatment outcomes for recurrent acute otitis media were also considered. None of the studies demonstrated a significant benefit from using a particular antibiotic (moderate quality evidence). Specifically, five individual RCTs compared different antibiotic treatments: amoxicillin-clavulanic acid versus gatifloxacin (2 RCTs), amoxicillin-clavulanic acid versus levofloxacin (1 RCT), amoxicillin-clavulanic acid versus azithromycin (1 RCT), and cefaclor versus cefuroxime (1 RCT). The evidence suggests that there are no substantial differences in clinical efficacy between these classes of antibiotics. Therefore, the NICE guidelines recommend that the choice of antibiotic should primarily be guided by the goal of minimising the risk of resistance.

### Recommendation 6

In children and adolescents with recurrent acute otitis media, amoxicillin-clavulanate for 7—10 days is recommended as the first choice for episodes of AOM. *(Moderate quality of evidence. Weak recommendation in favour of intervention).*

#### Question 7. Is antibiotic prophylaxis effective in reducing recurrence in RAOM?

A systematic literature review was conducted to identify studies evaluating antibiotic prophylaxis for reducing RAOM recurrence in children. The initial screening of 35 papers included one guideline, no systematic reviews, and 8 relevant studies (see supplementary information files).

The 2018 NICE guidelines do not provide specific recommendations for preventing recurrent AOM. In contrast, the Italian guidelines [8] indicate that although some studies demonstrate a statistically significant reduction in RAOM episodes in children receiving antibiotic prophylaxis compared to placebo, the limited number of AOM events prevented, the risk of antibiotic adverse events, and the potential for developing resistant bacteria contraindicate such prophylaxis except in selected cases (Weak negative recommendation).

Regarding the studies included in this systematic review, only the study by Koivunen et al. [39] reported a decrease in RAOM recurrences after 1 to 2 years of follow-up. In this study of 662 children, reductions in recurrence rates ranged from 18 to 73%, and the duration of endotympanic effusion was decreased by 33–41% in children receiving amoxicillin prophylaxis compared to placebo. The study concluded that amoxicillin prophylaxis is the most effective method for reducing recurrent otitis media in children compared to other surgical procedures. Particularly in children under two years of age, antibiotic prophylaxis is beneficial in reducing the prevalence and frequency of otitis media recurrences, and the total time children experience otitis media.

Several other studies [40, 41] have demonstrated some efficacy in reducing AOM recurrences among children receiving antibiotic treatment. However, these studies are limited by small sample sizes, often lack blinding, and were primarily conducted in the 1980s [42, 43], when vaccination coverage was not widespread. Consequently, many of these studies do not establish definite efficacy. A significant limitation is that most cultures taken during AOM recurrences isolated pneumococcus, which was more common before widespread vaccination, further limiting these studies’ generalizability and definitive conclusions.

A 2006 Cochrane systematic review [44], which included 17 studies involving 1,586 children at an unspecified increased risk of middle ear infections, concluded that continuous antibiotic prophylaxis (administered once or twice daily) only slightly reduced infection recurrences, from 3 to 1.5 episodes per year (a 21% reduction). This reduction was below the current recurrence threshold of 4 AOM episodes per year, even in untreated groups.

### Recommendations 7

7.1 Antibiotic prophylaxis should not be routinely recommended for children and adolescents with RAOM (*Moderate quality of evidence; Weak recommendation against intervention).*

7.2 In select cases with frequent recurrences where other invasive interventions are considered (e.g., tympanostomy tubes, adenotonsillectomy), antibiotic prophylaxis may be recommended as an alternative *(Low quality of evidence; Weak recommendation).*

#### Question 8. What is the first-line antibiotic for therapy of AOM/RAOM in patients allergic to penicillin?

The prevalence of proven penicillin allergy is low, ranging from 0,7 to 1%, and the anaphylaxis prevalence is about 0.004–0.015% [45–47]. The literature on penicillin allergy in children with AOM or other bacterial infections is limited. β-lactam hypersensitivity reactions can be classified based on the time elapsed between antibiotic intake and symptom onset: immediate reactions (within 1 h) are usually IgE-mediated and manifest as anaphylactic shock, angioedema, urticaria, and bronchospasm, which are considered high-risk symptoms. Delayed reactions (after 1 h) are typically non-IgE-mediated, with the most common clinical manifestation being a maculopapular rash, which is typically non-pruritic [45, 48].

The risk of cross-reactivity between penicillin and cephalosporins remains contentious [49]. Non-recent studies have reported a cross-reactivity frequency of 10% of cases. However, these studies were affected by evident biases, including the definition of allergy based solely on clinical history, the utilisation of reference preparations of cephalosporins that were still "crude" and potentially contaminated by penicillin, and a lack of precise knowledge regarding the molecular structures of the compounds involved. Studies utilising monoclonal antibodies have further revealed that the side chain represents the principal antigenic determinants for cefalosporine. Therefore, an increased risk of cross-reactions has not been demonstrated in cases where the side chains differ from those of penicillin or amoxicillin [50]. Supporting this, cefuroxime (a second-generation cephalosporin) has frequently been observed to be safe in patients with confirmed hypersensitivity to other beta-lactams, with cross-reactions occurring in only 6.3% of cases. Cross-reactions are more familiar with first-generation cephalosporins, which possess a beta-lactam ring similar to penicillins [51]. Given the low cross-reactivity observed in various studies, Drug Provocation Tests (DPT) are often conducted with cephalosporins featuring different side chains.

Although macrolide therapy can be effective in the treatment of AOM [52, 53], it is important to note that some of these studies were conducted many years ago, when the prevalence of pneumococcal resistance to macrolides was lower than it is today, and that only very high doses are effective [53].

T-lymphocytes play a role in non-IgE-mediated forms, and therefore, cross-reactivities are even rarer. 97.2% of individuals with hypersensitivity to aminopenicillins tolerate cephalosporins with different side chains, suggesting tolerability in most cases [50]. If penicillin allergy is confirmed, DPT with cephalosporins featuring different side chains may be considered.

In various allergy guidelines [44, 46, 52, 53] there is no definitive consensus on the optimal method to ascertain whether a patient with penicillin allergy can safely take a cephalosporin with different side chains directly or following DPT. However, the allergological assessment for a patient with penicillin allergy should ideally culminate in recommendations regarding the potential use of other beta-lactams, particularly cephalosporins. Knowing whether such use is feasible, is useful to pediatricians for their future therapeutic prescriptions.

Considering the limited evidence, children with AOM and penicillin allergy, whether IgE or non-IgE mediated, may be treated with cephalosporins with different side chains (such as cefuroxime or cefpodoxime proxetil) only after undergoing an adequate allergological workup to assess their potential use [50, 51]. Alternatively, depending on the local prevalence of pneumococcal resistance to specific drug classes, clindamycin, azithromycin, or, in older children, levofloxacin can be utilised [54].

### Recommendation 8

8.1 In patients with AOM/RAOM and a suspected allergy to amoxicillin who have not undergone allergological workup, the selection of alternative antibiotics (such as second/third-generation cephalosporins—cefuroxime or cefpodoxime proxetil) should be guided by meticulous risk stratification. *(Very low-quality evidence. Expert opinion. Strong recommendation in favour of the intervention. Panel consensus 100%).*

8.2 The use of macrolides (like clarithromycin or azytromicine) or clindamycin should be reserved for patients at high risk of allergic reactions. *(Very low-quality evidence. Expert opinion. Weak recommendation in favour of the intervention).*

#### Question 9. Are antibiotics administered parenterally (e.g. intramuscularly) more effective than oral amoxicillin/amoxicillin-clavulanic acid in treating AOM?

A large systematic review with meta-analysis of RCTs [31] found no significant differences in treatment efficacy between classes of antibiotics, including penicillins, cephalosporins and macrolides for uncomplicated acute otitis media in children. Specifically, there was no difference in treatment success between ampicillin or amoxicillin versus ceftriaxone; amoxicillin-clavulanic acid versus ceftriaxone; amoxicillin-clavulanic acid versus clarithromycin; or cefaclor versus azithromycin (low to moderate quality evidence). Amoxicillin-clavulanic acid was associated with significantly more adverse events than cephalosporin (very low to moderate quality evidence) or azithromycin (moderate quality evidence) [31]. In addition, many studies have documented no advantage of parenteral therapy over oral therapy when gastrointestinal absorption is normal, as blood levels of the drug are similar regardless of the route of administration for the same therapies given parenterally.

### Recommendation 9

For uncomplicated AOM, there are no substantial differences in efficacy between routes of administration, so parenteral antibiotics are not recommended. *(Moderate quality of evidence for penicillin vs. cephalosporins. Strong recommendation against intervention).*

#### Question 10. Can topical antibiotic therapy be helpful in acute perforated otitis?

No studies have analysed the effects of topical antibiotic therapy in cases of AOM with spontaneous perforation. The 2018 NICE guidelines only recommend using ear drops containing an anaesthetic and an analgesic, reporting no statistically significant differences in otalgia scores compared to usual care (low to very low-quality evidence). In the SIP guidelines [8] topical auricular antibiotic therapy, whether or not in combination with a steroid, is not recommended except in children with otorrhea from ventilation tubes. (Strong negative recommendation). The efficacy of intra-auricular antibiotic therapy has been evaluated by experimental research and clinical studies often on heterogeneous samples that included mainly subjects with external otitis and subjects with otitis media with or without perforation of the tympanic membrane, treated with different antibiotic combinations. Other studies of good methodological quality have evaluated the efficacy of topical antibiotic therapy only in a specific condition (otorrhea in AOM with ventilation tubes): their results are insufficient to hypothesise changes to antibiotic therapy in AOM, even if complicated by spontaneous perforation.

### Recommendation 10

In the absence of evidence of efficacy and safety, topical antibiotic therapy, in addition to oral antibiotic treatment, is not recommended in children and adolescents with AOM and spontaneous perforated otitis. *(Very low quality of evidence. Expert opinion. Strong recommendation against intervention).*

#### Question 11. Is an antibiotic other than amoxicillin indicated in acute perforated otitis?

The most common complications of acute otitis media are recurrence of the infection, hearing loss (usually temporary) and eardrum. However, according to NICE 2018 data, antibiotics make little difference to the risk of these complications [19]. In children with AOM with spontaneous perforation otorrhea, the relevance of β-lactamase-producing pathogens has led several authors to suggest using the combination of amoxicillin-clavulanic acid [53]. Even the 2019 SIP guidelines recommend, in cases of spontaneous perforation otorrhea and with increased risk of resistant pathogens (child community attendance, lack of anti-pneumococcal vaccination, geographical areas with a high prevalence of resistant bacteria), the use of amoxicillin-clavulanic acid at a dose of amoxicillin 40 mg/kg/day, supplemented with a plain amoxicillin formulation to ensure optimal doses of amoxicillin (80–90 mg/kg/day in 3 administrations) and clavulanic acid (< 10 mg/Kg/day) [8]. The addition to amoxicillin of clavulanic acid can effectively neutralise β-lactamase-producing micro-organisms such as *Haemophilus influenzae* and *Moraxella catarrhalis* while maintaining excellent antibacterial activity against penicillin-resistant strains of *S. pneumoniae* [55, 56]^.^

Available evidence [8, 57] indicates AOM with spontaneous membrane perforation is a condition of greater severity, more correlated with β-lactamase-resistant microorganisms for which amoxicillin-clavulanic acid is generally recommended.

### Recommendation 11

Based on epidemiological data, considering the frequent aetiology from β-lactamase-producing bacteria, the combination of amoxicillin-clavulanic acid should be recommended for cases of severe AOM with spontaneous perforation otorrhea. Amoxicillin-clavulanic acid at a dose of 40 mg/kg/day amoxicillin, supplemented with a plain amoxicillin formulation to ensure optimal doses of amoxicillin (80–90 mg/kg/day in 3 administrations) and clavulanic acid (< 10 mg/Kg/day) *(Low quality of evidence. Expert opinion. Weak recommendation in favour of intervention).*

## Conclusions

The diagnosis of AOM is based primarily on clinical and otoscopic assessment, which cannot reliably distinguish between viral and bacterial infection [58, 59]. This pose a significant challenge when establishing the therapy, including antibiotic treatment. It is generally recommended to assess the need for antibiotics before prescribing them. The optimal therapeutic approach in middle ear infections involves a 'watchful waiting' period to avoid over-prescription of antibiotics in otalgia and inflammation due to viral infection. Pediatricians often disregard the recommendation by the scientific community emphasising the use of amoxicillin as first-line treatment for patients with AOM who need antibiotics.

Instead, they often prefer broader-spectrum antibiotics such as amoxicillin-clavulanic acid, second- or third-generation cephalosporins or macrolides [5]. Moreover, there is no clear consensus on the optimal dosage and duration of therapy, highlighting the need for further studies to enhance our understanding on this issue. To improve the proper management of antibiotics in children with AOM or RAOM treated on an outpatient basis, this updated consensus was developed after meticulously evaluating of the best available evidence and existing guidelines, which the expert group thoroughly evaluated and discussed (Table 2).

**Table 2:** Summary of recommendations

1- The age of the child influences the treatment strategy for AOM a. in infants < 6 months immediate antibiotic therapy is recommended b. in children aged 6–24 months with bilateral AOM immediate prescription is recommended c. in children > 6–24 months with mild unilateral AOM a watchful waiting strategy is recommended d. For children older than 24 months and adolescents with AOM: A watchful waiting strategy is recommended in all cases without comorbidities, risk factors or specific conditions of severity e. For children older than 6 months where watchful waiting is indicated, it is recommended only if follow-up within 72 h is possible and comorbidities are absent
2- The severity of the AOM episode influences the timing of antibiotic therapy a. In children and adolescents with AOM, immediate antibiotic therapy is recommended in: - AOM with otorrhea - severe bilateral AOM - AOM with systemic symptoms or impaired general status b. In children > 24 months and adolescents the "watchful waiting" strategy is recommended in - severe unilateral AOM without systemic symptoms, or impaired general status, or otorrhea - unilateral or bilateral AOM without signs of severity
3- When the antibiotic therapy is necessary, then amoxicillin is recommended as the first-choice, prescribed, at 80–90 mg/kg/day in three doses (in uncomplicated AOM, with no risk factors for bacterial resistance or history of recurrence)
4- Duration of effective therapy for treating AOM in children and adolescents a. amoxicillin (or amoxi-clav) should be administered for 7–10 days in high-risk or severe conditions b. a shorter course of 5 days may be recommended in children without risk factors (older than 2 years, no otorrhea, no recurrence, no bilaterality, no severe symptoms,…)
5- The antibiotic recommended for children who do not recover or experience short-term relapse (within 30 days)), or whose symptoms worsen after at least 2–3 days of amoxicillin 6- combination of amoxicillin-clavulanate 40 mg/kg/day of amoxicillin (0.5ml/kg/day) plus plain amoxicillin formulation 50 mg/kg/d (1 ml/kg) in 3 administrations to ensure optimal doses
7- First-choice antibiotic therapy for an acute episode in children with recurrent AOM In recurrent AOM amoxicillin-clavulanate for 7—10 days is recommended as the first choice
8- Antibiotic prophylaxis in recurrent AOM should not be recommended for children and adolescents Antibiotic prophylaxis may be recommended as an alternative in select cases with frequent recurrences where other invasive interventions are considered (e.g., tympanostomy tubes)
9- The first-line antibiotic for therapy of AOM/RAOM in patients allergic to penicillin a. the choice of alternative antibiotics (such as 2nd/3rd-generation cephalosporins—cefuroxime or cefpodoxime proxetil) should be guided by risk stratification b. use of macrolides or clindamycin should be reserved for patients at high risk of allergic reactions
10- Parenterally administered antibiotics are no more effective than oral amoxicillin/clavulanic acid in the treatment of AOM (no substantial differences in efficacy) so they are not recommended
11- Topical antibiotic therapy is not considered helpful in acute perforated otitis
12- In the absence of evidence of efficacy and safety topical antibiotic therapy is not recommended
13- The combination of amoxicillin-clavulanic acid should be recommended for cases of severe AOM with spontaneous perforation otorrhea (suspected β-lactamase-producing bacteria) Amoxicillin-clavulanic acid at a dose of 40 mg/k/d amoxi, supplemented with plain amoxicillin formulation to ensure amoxicillin (80–90 mg/k/d in 3 doses) and clavulanic acid (< 10 mg/K/d)

Several limitations persists, and priorities for further studies should focus on key areas:

1. improve diagnostic accuracy: research should aim to refine diagnostic tools, including appropriate devices, point-of-care tests, and methods to accurately distinguish between viral and bacterial AOM in a clinical setting.
2. promoting antibiotic stewardship: Studies should explore the factors leading to the frequent use of broader-spectrum antibiotics instead of the recommended first-line treatments, such as amoxicillin.
3. evaluating therapeutic approaches: Further research should evaluate the efficacy of different antibiotic regimens, including the recommended first-line treatment with amoxicillin, verify antibiotic therapy’s optimal dosage and duration, evaluate special group populations as children with immunodeficiencies. Clinical trials are needed to establish treatment protocols that minimise side effects and resistance while maximising therapeutic outcomes.
4. Addressing barriers to guideline adherence: Understanding why first-line treatment recommendations are often disregarded and developing strategies to improve adherence among healthcare professionals is crucial.

## Supplementary Information

Additional file 1. S1_AOM GLs, SRs, Studies Search.pdf (PICOs, GRADE Adolopment, Keywords, Guidelines search, Systematic review search, studies search, algorithm for guidelines search, Algorithm for Systematic Review search, Algorithm for Studies search).

Additional file 2. S2_RAOM Antibiotic prophylaxis GLs, SRs, Studies Search.pdf (PICOs, Keywords, Guidelines search, Systematic Review Search, Studies search, Algorithm for Guidelines search, Algorithm for Systematic Review search, Algorithm for Studies search).

Additional file 3. S3 S3_ AOM-RAOM_GLs, SRs, Studies Appraisal.pdf (methodological assessment).

Additional file 4. S4_AOM-RAOM RESULTS.pdf (guidelines reccomendations, RSs and studies results).

Additional file 5. S5_RAOM META-ANALYSIS.pdf (RAOM antibiotic prophylaxis meta-analysis).

Additional file 6. S6_AOM-RAOM GRADE.pdf (RAOM GRADE evidence profile).

Additional file 7. S7_AOM-RAOM References of tables.pdf (reference of tables).

Additional file 8. S8_Italian intersociety consensus_Antibiotics_STRUCTURE AND METHODOLOGY OF THE DOCUMENT.pdf (Itaian Intersociety consensus on antibiotic therapy of respiratory infections in pediatric age).

## Data Availability

All data generated or analysed during this study are included in this published article and its supplementary information files S1-S8, where the following topics can be found for each group of recommendations: evidence search; selection and appraisal; list of the studies excluded with relevant reasons; list, characteristics, and results of the SRs of the studies included; the GRADE tables; references of tables; and structure and methodology of the document.

## Abbreviations

AAP: American Academy of Pediatrics
AOM: Acute Otitis Media
DPT: Drug Provocation Test
EOM: Exudative Otitis Media
GL: Guideline
GRADE: Grading of Recommendations Assessment, Development, and Evaluation
HICs: High-Income Countries
IDSA: Infectious Diseases Society of America
LMICs: Low- or Middle-Income Countries
MIC: Minimum Inhibitory Concentration
NICE: National Institute for Health and Care Excellence
NNT: Number Needed to Treat
PRISMA: Preferred Reporting Items for Systematic Reviews and Meta-analyses
RAOM: Recurrent Acute Otitis Media
RCT: Randomized Clinical Trial
RS: Randomized study
SIP: Italian Society of Pediatrics
TM: Tympanic membrane
