# Supplementary material for: Antibiotic treatment of acute and recurrent otitis media in children: an Italian intersociety Consensus
Source: Ital J Pediatr. 2025 Feb 20;51:50. doi: 10.1186/s13052-025-01894-z (PMC11844117; doi:10.1186/s13052-025-01894-z)

S1. ACUTE OTITIS MEDIA (AOM)

PICOs

Question 1: **Does the child's age influence the type of therapeutic strategy to adopt in AOM?**

P In children under 24 months of age diagnosed with AOM
I does watchful waiting
C compared with immediate antibiotic therapy
O1 delay the resolution of symptoms?
O2 alter the risk of suppurative complications?
O3 increase the risk of short-term relapse?
O4 increase the risk of RAOM?

Question 2: **Does the severity of the AOM episode have an effect on the timing of antibiotic treatment?**

P1 In children diagnosed with AOM and fever >39°C
P2 In children diagnosed with AOM and otorrhoea
P3 In children diagnosed with AOM and impaired general condition
P4 In children diagnosed with bilateral AOM
I does the watchful waiting strategy
C compared with immediate antibiotic therapy
O1 delay the resolution of symptoms?
O2 alter the risk of suppurative complications?
O3 increase the risk of short-term relapse?
O4 increase the risk of RAOM?

Question 3: **If antibiotic therapy is necessary, can amoxicillin still be considered the first choice in children with acute otitis media?**

P In a child with AOM requiring antibiotic therapy
I does the use of amoxicillin versus
C1 amoxicillin/clavulanate
C2 2nd generation cephalosporins
C3 3rd generation cephalosporins
C4 macrolides
O1 reduce the cure rate
O2 alter the risk of suppurative complications?
O3 increase the risk of short-term relapse?
O4 increase the risk of RAOM?

Question 4: **Is the use of amoxicillin at a dose of 80-90 mg/kg per day more effective than the use of amoxicillin at a dose of 50 mg/kg per day?**

P In children with AOM
I is the use of amoxicillin at a dose of 80-90 mg/kg per day

C compared with a dose of 50 mg/kg per day
O1 equally effective in achieving a cure?
O2 alter the risk of suppurative complications?
O3 increase the risk of short-term relapse?
O4 increase the risk of RAOM?

Question 5: **Should amoxicillin or amoxicillin/clavulanate therapy be divided into 2 or 3 daily doses?**

P In children with AOM
I1 does fractionation of amoxicillin therapy into two daily doses
I2 does fractionation of amoxicillin/clavulanate therapy into two daily doses
C compared with fractionation into three daily doses
O1 increase therapeutic adherence?
O2 increase the risk of treatment failure?
O3 increase the risk of suppurative complications?
O4 increase the risk of short-term relapse?
O5 increase the risk of RAOM?

Question 6: **For children with AOM, is a 5-day or 10-day course of antibiotics more effective?**

P1 In children with AOM and age < 24 months
P2 In children with AOM and age ≥ 24 months
I1 does a duration of antibiotic therapy of 5 days
I2 does a duration of antibiotic therapy of 7 days
C compared with a duration of more than 10 days
O1 increase therapeutic adherence?
O2 increase the risk of therapeutic failure?
O3 increase the risk of suppurative complications?
O4 increase the risk of short-term relapse?
O5 increase the risk of RAOM?
O6 reduce the risk of adverse events (allergic/ urticarial reactions, antibiotic diarrhea, etc.)?

Question 7: **Which antibiotic is recommended for children with AOM who do not recover or who have a short-term relapse after amoxicillin therapy at 80-90mg/kg/day?**

P In children with AOM who do not recover or who have a short-term relapse after amoxicillin therapy at 80-90mg/kg/day
I1 is therapy with 2nd generation cephalosporins
I2 is therapy with 3rd generation cephalosporins
I3 is therapy with macrolides
I4 is therapy with quinolones
C compared with amoxicillin/clavulanate therapy
O1 more effective in achieving a cure?
O2 more effective in reducing the risk of short-term relapse?
O3 more effective in reducing the risk of RAOM?
O4 more effective in reducing the risk of suppurative complications?

Question 8: **What is the antibiotic therapy of first choice for an episode of AOM in children with RAOM?**

P In children with RAOM
I is antibiotic therapy with a different antibiotic
C compared with first-choice antibiotic amoxicillin or second-choice antibiotic amoxicillin/clavulanate (worsening of symptoms with first-choice therapy taken for at least 2 or 3 days)
O1 more effective in achieving a cure?
O2 more effective in reducing the risk of short-term relapse?
O3 most effective in reducing the risk of RAOM?
O4 most effective in reducing the risk of suppurative complications?

Question 9: **In children with RAOM, is the use of antibiotic prophylaxis effective in reducing the recurrence of episodes?**

P In children with RAOM, is treatment with
I1 long-term antibiotic prophylaxis with amoxicillin or amoxicillin/clavulanate or
I2 long-term antibiotic prophylaxis with other antibiotics (e.g. macrolides, co-trimoxazole)
C versus antibiotic treatment of each episode
Or more effective in reducing the recurrence of episodes?

Question 10: **Which antibiotics can be used to treat AOM/RAOM in children who are allergic to penicillin?**

P In penicillin-allergic children with AOM/RAOM,
I is therapy with
I1 2nd generation cephalosporins or
I2 3rd generation cephalosporins or
I3 quinolones
C compared with macrolides
O1 equally/more effective in achieving a cure?
O2 equally/more effective in reducing the risk of short-term relapse?
O3 equally/more in reducing the risk of RAOM?
O4 equally/more in reducing the risk of suppurative complications?

Question 11: **Are antibiotics given parenterally, specifically intramuscularly, more effective than amoxicillin/amoxicillin-clavulanic acid given orally (po) in treating AOM?**

P In children with AOM
I is therapy with antibiotics by injection (ceftriaxone or other cephalosporins i.m.)
C compared with therapy with amoxicillin/amoxicillin-clavulanic acid po
O1 significantly more effective and faster in achieving a cure?
O2 significantly more effective in reducing the risk of short-term relapse?
O3 significantly more effective in reducing the risk of RAOM?
O4 significantly more effective in reducing the risk of suppurative complications?

Question 12: **Can topical antibiotics be useful in the treatment of acute perforated otitis media?**

P In children with AOM and otorrhea
I is combined topical and oral therapy
C compared with oral therapy alone
O1 more effective in achieving a cure?
O2 more effective in reducing the risk of short-term relapse?
O3 more effective in reducing the risk of RAOM?
O4 more effective in reducing the risk of suppurative complications?

Question 13: **In acute perforated otitis media, is an antibiotic other than amoxicillin indicated?**

P In children with perforated AOM
I1 is amoxicillin-clavulanic acid or
I2 cephalosporin or
I3 quinolone
C compared to amoxicillin
O1 more effective in achieving a cure?
O2 more effective in reducing the risk of short-term relapse?
O3 more effective in reducing the risk of RAOM?
O4 more effective in reducing the risk of suppurative complications?

S1.1. GRADE ADOLOPMENT

Translated by: *Journal of Clinical Epidemiology* 2017 81, 101-110 DOI: 10.1016/j.jclinepi.2016.09.00

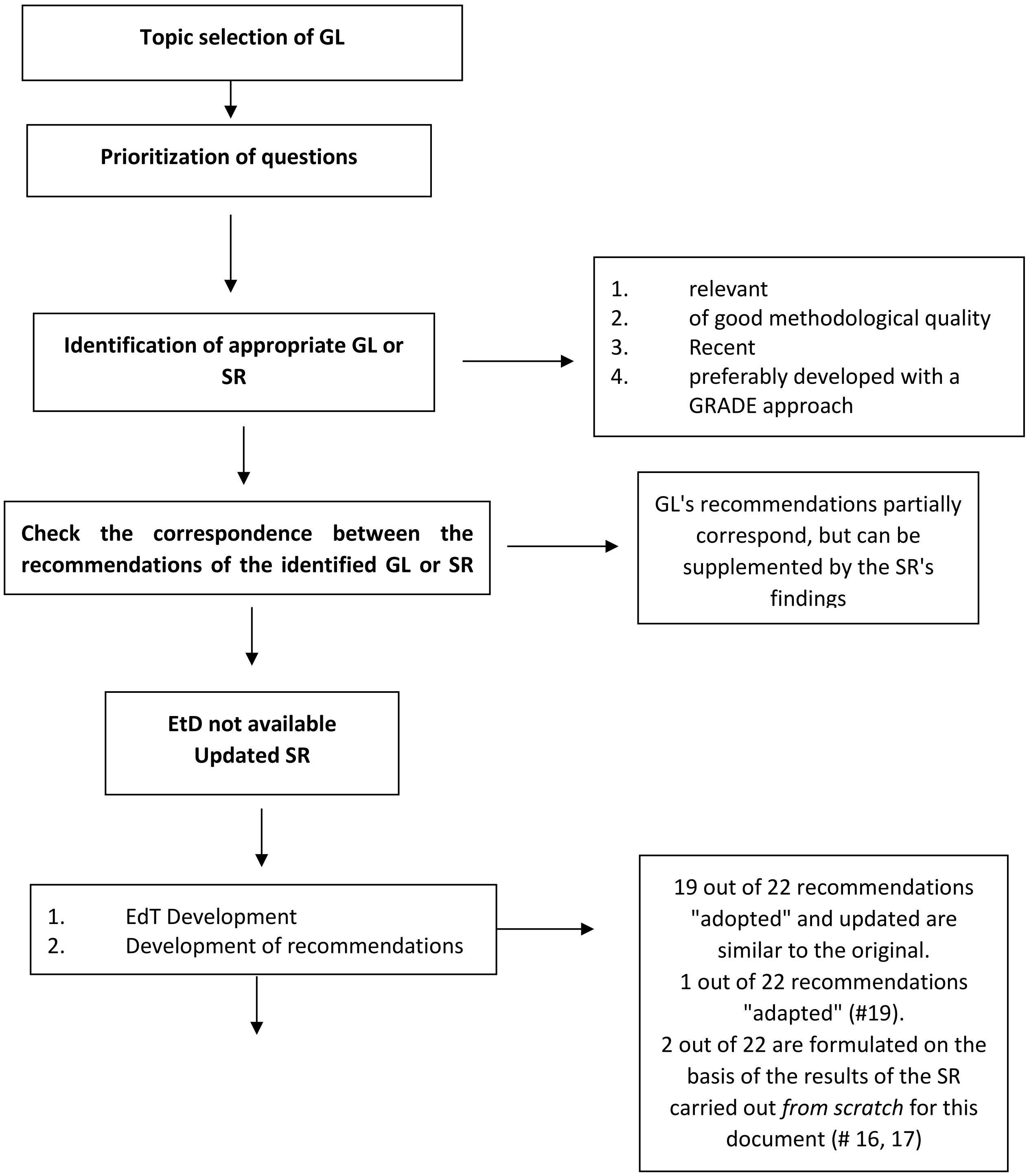

KEYWORDS

Population

A. 0-18 years old
B. infant, child, adolescent

Exposure factors/Comparison

A. Anti-bacterial agents
B. Amoxicillin
C. amoxicillin potassium clavulanate
D. Penicillin
E. Benzylpenicillin
F. Cephalosporins
G. Macrolides
H. Cefuroxime
I. Erythromycin
J. Azithromycin
K. Clarithromycin
L. Sulfamethoxazole drug combination
M. Trimethoprim
N. Administration
O. Dosage

Outcomes

A. otitis media
B. acute otitis media
C. middle ear effusion
D. middle ear inflammation
E. middle ear infection
F. otitis media with effusion
G. glue ear
H. otorrhea
I. earache
J. ear pain
K. adverse effects

Guidelines search

Time interval: 2017-2022

UPTODATE https://www.uptodate.com/home

Society Guideline Links

SNLG https://snlg.iss.it/

National Guideline Centre (NGC) - National Institute of Health and Care Excellence (NICE) https://www.rcplondon.ac.uk/about-us/what-we-do/national-guideline-centre-ngc

Australian Clinical Practice Guidelines (ACPG) https://www.clinicalguidelines.gov.au/

MJA (Medical Journal of Australia) Clinical Guidelines https://www.mja.com.au/journal/guidelines

Canadians Medical Association (CMA) https://www.cma.ca/clinicalresources/practiceguidelines

G-I-N Guidelines International Network https://g-i-n.net/

New Zealand Guidelines Group (NZGG) https://www.health.govt.nz/about-ministry/ministry-health-websites/new-zealand-guidelines-group

Scottish Intercollegiate Guidelines Network (SIGN) https://www.sign.ac.uk/our-guidelines.html

Guidelines Central https://www.guidelinecentral.com/

Società Italiana di Pediatria (SIP) http://www-sip.it/

Società Italiana di Pediatria Preventiva e Sociale (SIPPS) https://www.sipps.it/

American Academy of Pediatrics (AAP) https://www.aap.org/en-us/Pages/Default.aspx

European Paediatric Association /Union of National European Paediatric Societies and Associations EPA-UNEPSA http://www.epa-unepsa.org/

PubMed https://www.ncbi.nlm.nih.gov/pubmed/

("Otitis Media"[MeSH Terms] OR "acute otitis media"[All Fields] AND ((Practice Guideline[ptyp] OR Guideline[ptyp]) AND "2017/06/30"[PDat] : "2022/06/30"[PDat] AND (allchild[Filter])

EMBASE https://www.embase.com

('acute otitis media'/exp OR 'acute otitis media' AND 'practice guideline'/de AND ([adolescent]/lim OR [child]/lim OR [infant]/lim OR [newborn]/lim OR [preschool]/lim OR [school]/lim) AND [2017-2022]/py

Systematic Review Search

Time interval: 2016-2022

PubMed https://www.ncbi.nlm.nih.gov/pubmed/

("Otitis Media"[MeSH Terms] OR "acute otitis media"[All Fields] OR "middle ear inflammation"[All Fields] OR "middle ear infection"[All Fields] OR "glue ear"[All Fields] OR "otorrhea"[All Fields] OR ("Earache"[MeSH Terms] OR "ear pain"[All Fields] OR (("ear"[MeSH Terms] OR "ear"[All Fields]) AND ("pain"[MeSH Terms] OR "pain"[All Fields] OR "aches"[All Fields] OR "aching"[All Fields])))) AND ((meta-analysis[Filter] OR systematicreview[Filter]) AND (2016/12/01:2022/6/30[pdat]) AND (allchild[Filter]))

EMBASE https://www.embase.com

('otitis media' OR 'acute otitis media'/exp) AND ([systematic review]/lim OR [meta analysis]/lim) AND ([adolescent]/lim OR [child]/lim OR [infant]/lim OR [newborn]/lim OR [preschool]/lim OR [school]/lim) AND [2016-2022]/py

Studies search (since the closure of the research in the guidelines evidence-based NICE 2018)

Time interval: 2016-2022

PubMed https://www.ncbi.nlm.nih.gov/pubmed/

("Otitis Media"[MeSH Terms] OR "acute otitis media"[All Fields] OR ("middle ear"[All Fields] OR "middle ear effusion"[All Fields] OR "middle ear inflammation"[All Fields] OR "middle ear infection"[All Fields]) OR ("otitis media with effusion"[MeSH Terms] OR ("otitis"[All Fields] AND "media"[All Fields] AND "effusion"[All Fields]) OR "otitis media with effusion"[All Fields] OR ("glue"[All Fields] AND "ear"[All Fields]) OR "glue ear"[All Fields]) OR ("otorrhea"[All Fields] OR "otorrhoea"[All Fields]) OR ("Earache"[MeSH Terms] OR "ear pain"[All Fields] OR (("ear"[MeSH Terms] OR "ear"[All Fields]) AND ("pain"[MeSH Terms] OR "pain"[All Fields] OR "aches"[All Fields] OR "aching"[All Fields]))))

AND (("Anti-Bacterial Agents"[Mesh]) OR ("amoxicillin"[MeSH Terms] OR "amoxicillin"[All Fields] OR "amoxicilline"[All Fields] OR "amoxicillins"[All Fields] OR ("cefuroxime"[MeSH Terms] OR "cefuroxime"[All Fields] OR "cefuroxim"[All Fields]) OR ("erythromycin"[MeSH Terms] OR "erythromycin"[All Fields] OR "erythromycins"[All Fields] OR "erythromycine"[All Fields]) OR ("azithromycin"[MeSH Terms] OR "azithromycin"[All Fields] OR "azithromycine"[All Fields] OR "azithromycin s"[All Fields]) OR ("clarithromycin"[MeSH Terms] OR "clarithromycin"[All Fields] OR "clarithromycine"[All Fields]) OR ("amoxicillin potassium clavulanate combination"[MeSH Terms] OR ("amoxicillin potassium"[All Fields] AND "clavulanate"[All Fields] AND "combination"[All Fields]) OR "amoxicillin potassium clavulanate combination"[All Fields] OR ("amoxicillin"[All Fields] AND "potassium"[All Fields] AND "clavulanate"[All Fields] AND "combination"[All Fields]) OR "amoxicillin potassium clavulanate combination"[All Fields]) OR ("amoxicillin*"[All Fields] OR "amix"[All Fields] OR ("amoxicillin"[MeSH Terms] OR "amoxicillin"[All Fields] OR "amoxil"[All Fields])) OR ("cefuroxime*"[All Fields] OR ("cefuroxime"[MeSH Terms] OR "cefuroxime"[All Fields] OR "zinacef"[All Fields]) OR ("cefuroxime axetil"[Supplementary Concept] OR "cefuroxime axetil"[All Fields] OR "zinnat"[All Fields])) OR ("erythromycin*"[All Fields] OR ("buserelin"[MeSH Terms] OR "buserelin"[All Fields] OR "tiloryth"[All Fields]) OR "primacine"[All Fields] OR ("erythromycin"[MeSH Terms] OR "erythromycin"[All Fields] OR "erymax"[All Fields]) OR ("erythrocin"[All Fields] OR "erythromycin"[MeSH Terms] OR "erythromycin"[All Fields] OR "erythromycins"[All Fields] OR "erythromycine"[All Fields]) OR ("erythromycin ethylsuccinate"[MeSH Terms] OR ("erythromycin"[All Fields] AND "ethylsuccinate"[All Fields]) OR "erythromycin ethylsuccinate"[All Fields] OR "erythroped"[All Fields]) OR ("erythromycin ethylsuccinate"[MeSH Terms] OR ("erythromycin"[All Fields] AND "ethylsuccinate"[All Fields]) OR "erythromycin ethylsuccinate"[All Fields] OR "erythroped"[All Fields])) OR ("azithromycin*"[All Fields] OR ("azithromycin"[MeSH Terms] OR "azithromycin"[All Fields] OR "azithromycine"[All Fields] OR "zithromax"[All Fields] OR

"azithromycin s"[All Fields])) OR ("clarithromycin*"[All Fields] OR "klaricid"[All Fields] OR "XL"[All Fields] OR ("amoxicillin potassium clavulanate combination"[MeSH Terms] OR ("amoxicillin potassium"[All Fields] AND "clavulanate"[All Fields] AND "combination"[All Fields]) OR "amoxicillin potassium clavulanate combination"[All Fields] OR "coamoxiclav"[All Fields]) OR "co-amoxiclav"[All Fields] OR ("amoxicillin"[MeSH Terms] OR "amoxicillin"[All Fields] OR "amoxicilline"[All Fields] OR "amoxicillin potassium clavulanate combination"[MeSH Terms] OR ("amoxicillin potassium"[All Fields] AND "clavulanate"[All Fields] AND "combination"[All Fields]) OR "amoxicillin potassium clavulanate combination"[All Fields] OR "augmentin"[All Fields] OR "amoxicillins"[All Fields] OR "augmentine"[All Fields] OR "clavulanic acid"[MeSH Terms] OR ("clavulanic"[All Fields] AND "acid"[All Fields]) OR "clavulanic acid"[All Fields] OR "clavulanate"[All Fields] OR "clavulanic"[All Fields])) OR ("trimethoprim, sulfamethoxazole drug combination"[MeSH Terms] OR ("trimethoprim"[All Fields] AND "sulfamethoxazole"[All Fields] AND "drug"[All Fields] AND "combination"[All Fields]) OR "sulfamethoxazole drug combination trimethoprim"[All Fields] OR ("trimethoprim"[All Fields] AND "sulfamethoxazole"[All Fields] AND "drug"[All Fields] AND "combination"[All Fields]) OR "trimethoprim sulfamethoxazole drug combination"[All Fields] OR ("cotrimoxazol"[All Fields] OR "trimethoprim, sulfamethoxazole drug combination"[MeSH Terms] OR ("trimethoprim"[All Fields] AND "sulfamethoxazole"[All Fields] AND "drug"[All Fields] AND "combination"[All Fields]) OR "sulfamethoxazole drug combination trimethoprim"[All Fields] OR "cotrimoxazole"[All Fields] OR "Co-trimoxazole"[All Fields] OR ("trimethoprim, sulfamethoxazole drug combination"[MeSH Terms] OR ("trimethoprim"[All Fields] AND "sulfamethoxazole"[All Fields] AND "drug"[All Fields] AND "combination"[All Fields]) OR "sulfamethoxazole drug combination trimethoprim"[All Fields] OR "septrin"[All Fields]))) OR ("macrolid"[All Fields] OR "macrolides"[MeSH Terms] OR "macrolides"[All Fields] OR "macrolide"[All Fields] OR "macrolids"[All Fields]) OR "macrolide*"[All Fields] OR ("benzylpenicillins"[All Fields] OR "penicillin g"[MeSH Terms] OR "penicillin g"[All Fields] OR "benzylpenicillin"[All Fields] OR "penicilline"[All Fields] OR "penicillines"[All Fields] OR "penicillins"[MeSH Terms] OR "penicillins"[All Fields] OR "penicillin"[All Fields]) OR "penicillin*"[All Fields] OR ("cephalosporine"[All Fields] OR "cephalosporines"[All Fields] OR "cephalosporins"[MeSH Terms] OR "cephalosporins"[All Fields] OR "cephalosporin"[All Fields]) OR "cephalosporin*"[All Fields])

AND ((2016/12/01:2022/6/30[pdat])
AND (allchild[Filter]))
AND ((controlledclinicaltrial[Filter] OR randomizedcontrolledtrial[Filter])

EMBASE https://www.embase.com

('otitis media' OR 'acute otitis media'/exp) AND ('antibiotic agent'/exp OR 'antibiotic therapy'/exp) AND ([randomized controlled trial]/lim OR 'controlled clinical trial'/de) AND ([adolescent]/lim OR [child]/lim OR [infant]/lim OR [newborn]/lim OR [preschool]/lim OR [school]/lim) AND [2016-2022]/py

S1.2. Algorithm for Guidelines search

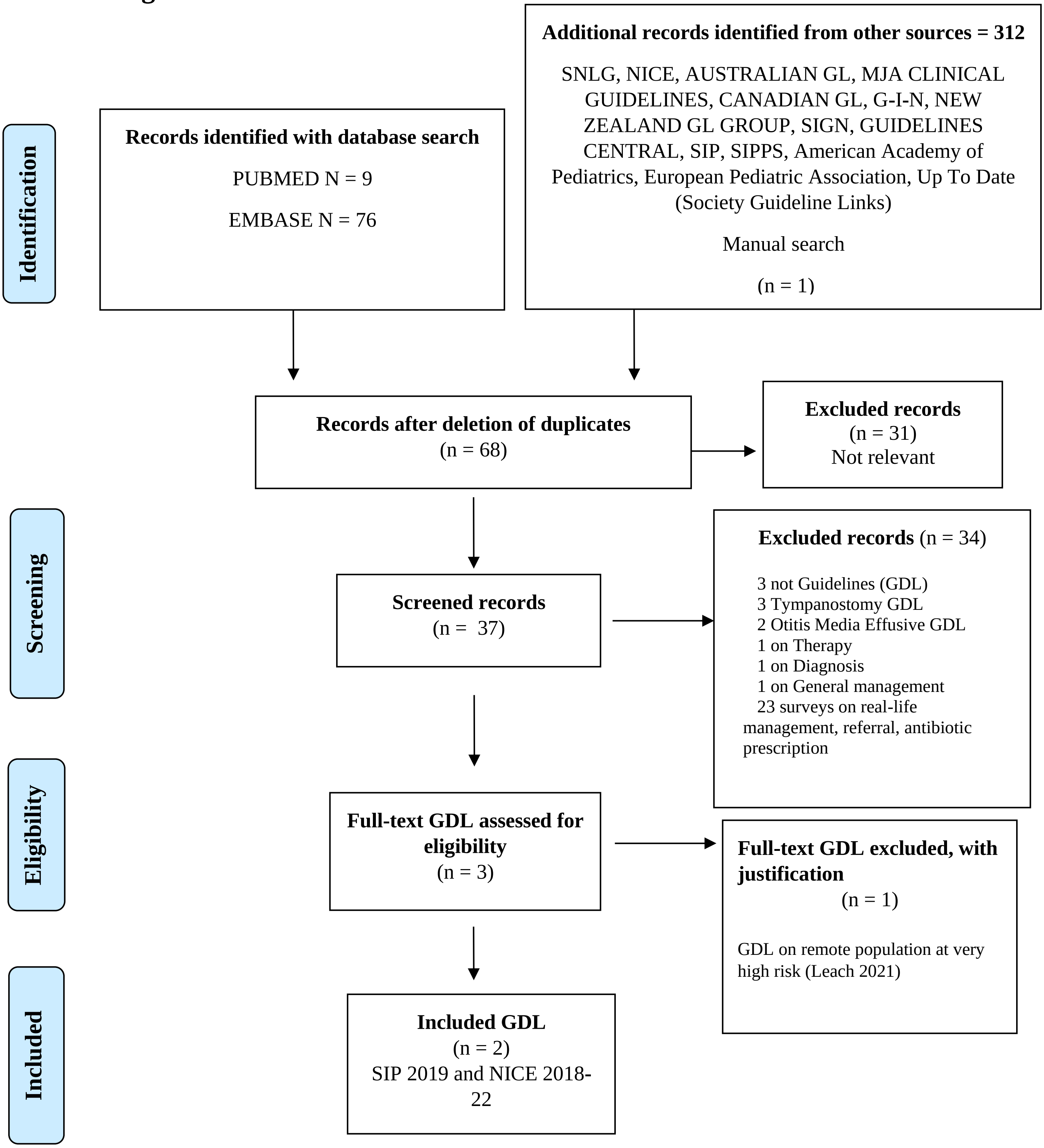

S1.3. Algorithm for Systematic Review (SR) search

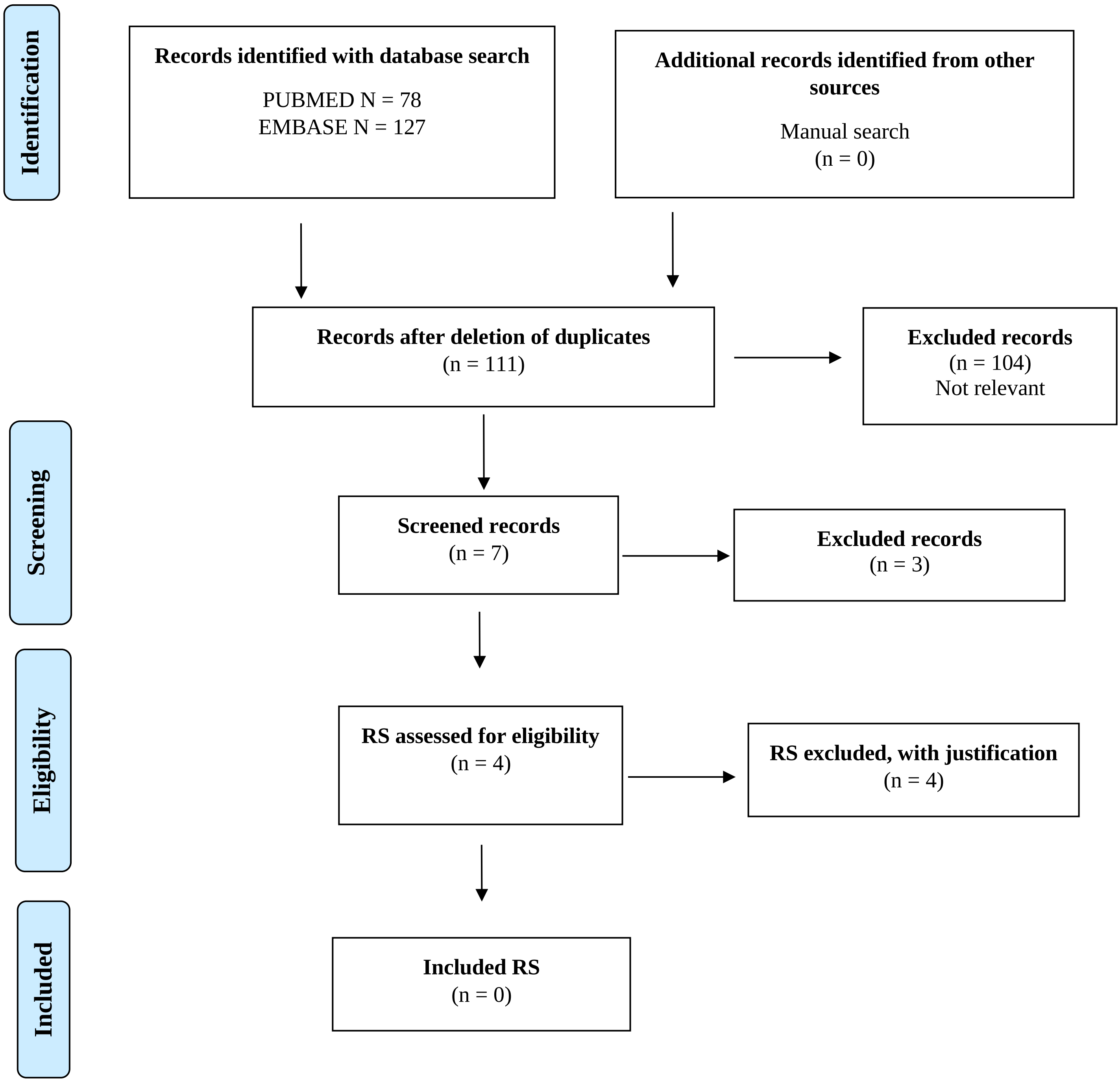

S1.4. Algorithm for Studies search

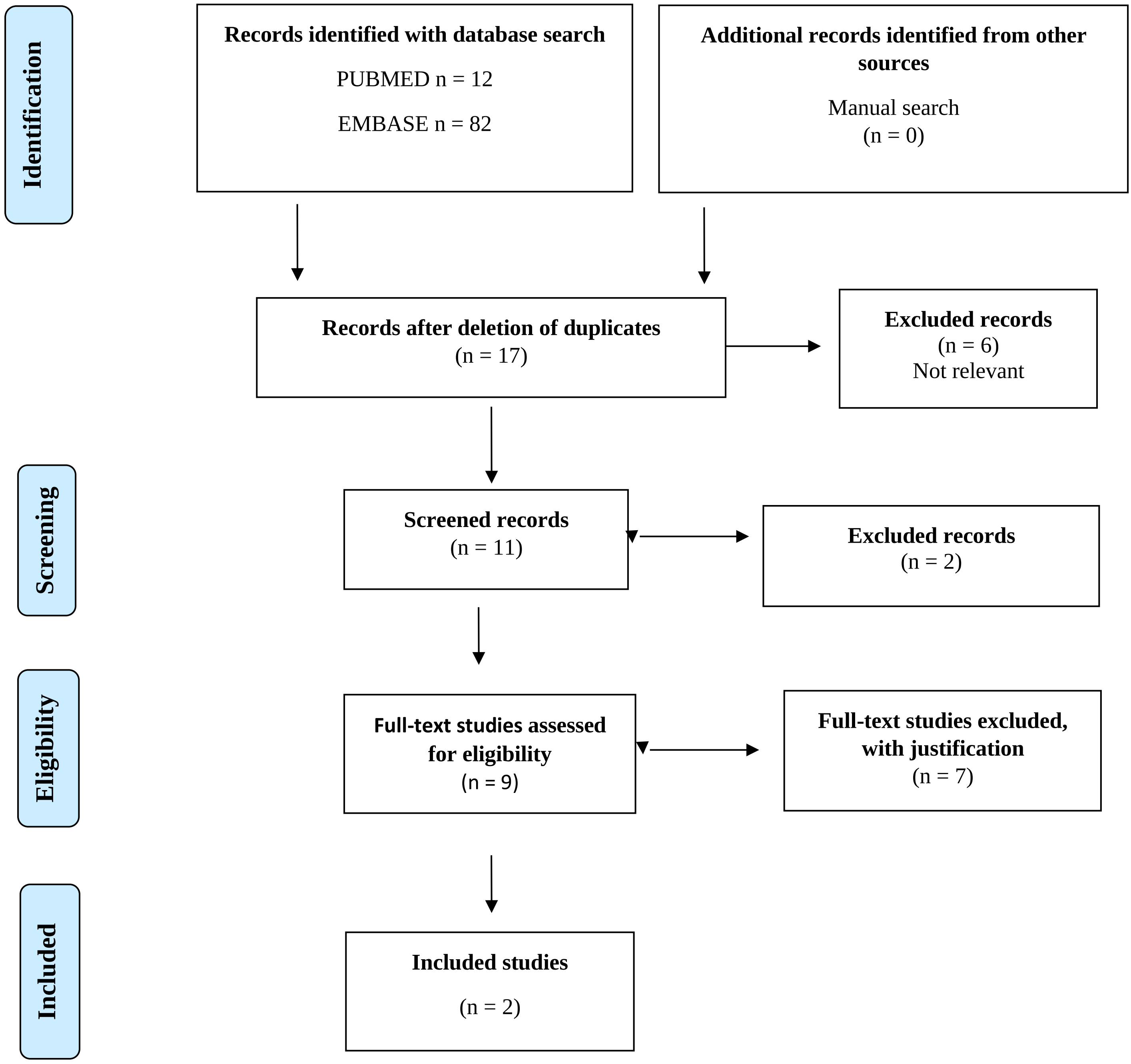

Supplement: Supplementary file 1 — Additional file 1. S1_AOM GLs, SRs, Studies Search.pdf (PICOs, GRADE Adolopment, Keywords, Guidelines search, Systematic review search, studies search, algorithm for guidelines search, Algorithm for Systematic Review search, Algorithm for Studies search). [file 13052_2025_1894_MOESM1_ESM.pdf]
