# Supplementary material for: Antibiotic treatment of acute and recurrent otitis media in children: an Italian intersociety Consensus
Source: Ital J Pediatr. 2025 Feb 20;51:50. doi: 10.1186/s13052-025-01894-z (PMC11844117; doi:10.1186/s13052-025-01894-z)

S2. RECURRENT ACUTE OTITIS MEDIA (RAOM)

Antibiotic prophylaxis

***Question 9*: Is antibiotic prophylaxis effective in reducing the recurrence of episodes in children with RAOM?**

PICOs

P: In children with RAOM
I: is medium to long-term antibiotic prophylaxis
C: compared with antibiotic treatment of single episodes alone
O: more effective in reducing the recurrence of episodes?

KEYWORDS

Population

A. 0-18 years old
B. infant, child, adolescent

A. Exposure factors/Comparison

B. Anti-bacterial agents
C. Antibiotic prophylaxis
D. Prophylactic antibiotics
E. Antimicrobial prophylaxis
F. Amoxicillin
G. Amoxicillin potassium clavulanate
H. Penicillin
I. Benzylpenicillin
J. Cephalosporins
K. Macrolides
L. Cefuroxime
M. Erythromycin
N. Azithromycin
O. Clarithromycin
P. Sulfamethoxazole drug combination
Q. Trimethoprim

Outcomes

A. otitis media
B. acute otitis media
C. recurrent acute otitis media
D. middle ear effusion
E. middle ear inflammation
F. middle ear infection
G. otitis media with effusion
H. glue ear

I. otorrhea
J. earache
K. ear pain
L. adverse effects

Guidelines search

<u>Time interval</u>: 2017-2022

UPTODATE https://www.uptodate.com/home

Society Guideline Links

SNLG https://snlg.iss.it/

National Guideline Centre (NGC) - National Institute of Health and Care Excellence (NICE) https://www.rcplondon.ac.uk/about-us/what-we-do/national-guideline-centre-ngc

Australian Clinical Practice Guidelines (ACPG) https://www.clinicalguidelines.gov.au/

MJA (Medical Journal of Australia) Clinical Guidelines https://www.mja.com.au/journal/guidelines

Canadians Medical Association (CMA) https://www.cma.ca/clinicalresources/practiceguidelines

G-I-N Guidelines International Network https://g-i-n.net/

New Zealand Guidelines Group (NZGG) https://www.health.govt.nz/about-ministry/ministry-health-websites/new-zealand-guidelines-group

Scottish Intercollegiate Guidelines Network (SIGN) https://www.sign.ac.uk/our-guidelines.html

Guidelines Central https://www.guidelinecentral.com/

Società Italiana di Pediatria (SIP) http://www-sip.it/

Società Italiana di Pediatria Preventiva e Sociale (SIPPS) https://www.sipps.it/

American Academy of Pediatrics (AAP) https://www.aap.org/en-us/Pages/Default.aspx

European Paediatric Association /Union of National European Paediatric Societies and Associations EPA-UNEPSA http://www.epa-unepsa.org/

PubMed https://www.ncbi.nlm.nih.gov/pubmed/

#1

("Otitis Media"[MeSH Terms] OR "acute otitis media"[All Fields] OR ("recurrent acute otitis media"[All Fields] AND ((Practice Guideline[ptyp] OR Guideline[ptyp]) AND "2017/06/30"[PDat] : "2022/06/30"[PDat] AND (allchild[Filter])

EMBASE

('acute otitis media'/exp OR 'acute otitis media' OR 'recurrent acute otitis media'/exp) AND 'practice guideline'/de AND ([adolescent]/lim OR [child]/lim OR [infant]/lim OR [newborn]/lim OR [preschool]/lim OR [school]/lim) AND [2017-2022]/py

Systematic Review Search

Time interval: 2012-2022

PubMed https://www.ncbi.nlm.nih.gov/pubmed/

#1

("recurrent acute otitis media"[All Fields] OR "RAOM"[All Fields] OR ("acute otitis media"[All Fields] AND "recurren*"[All Fields])) AND ((y_10[Filter]) AND (meta-analysis[Filter] OR systematic review[Filter]) AND (all child[Filter]))

EMBASE https://www.embase.com

1

(('acute otitis media'/exp OR 'acute otitis media') AND 'recurren*' OR 'recurrent acute otitis media'/exp OR 'raom') AND ([adolescent]/lim OR [child]/lim OR [infant]/lim OR [newborn]/lim OR [preschool]/lim OR [school]/lim) AND ([cochrane review]/lim OR [systematic review]/lim OR [meta analysis]/lim) AND [2012-2022]/py

2

'acute otitis media'/exp AND ([systematic review]/lim OR [meta analysis]/lim) AND [2012-2022]/py AND ([cochrane review]/lim OR [systematic review]/lim OR [meta analysis]/lim)

Studies search

Time interval: none

PubMed https://www.ncbi.nlm.nih.gov/pubmed/

1

((("recurrent acute otitis media"[All Fields] OR "RAOM"[All Fields]) AND ("Antibiotic Prophylaxis"[MeSH Terms] OR "Anti-Bacterial Agents"[MeSH Terms])) OR "prophylactic antibiotics"[All Fields] OR "antimicrobial prophylaxis"[All Fields]) AND ((controlledclinicaltrial[Filter] OR randomizedcontrolledtrial[Filter]) AND (allchild[Filter]))

2

(("recurrent acute otitis media"[All Fields] OR ("acute otitis media"[All Fields] AND "recurren*"[All Fields]))) AND (Therapy/Narrow[filter]) Filters: Controlled Clinical Trial, Randomized Controlled Trial, Child: birth-18 years

EMBASE https://www.embase.com

1

(('acute otitis media'/exp OR 'acute otitis media') AND 'recurren*' OR 'recurrent acute otitis media'/exp OR 'raom') AND ('antibiotic agent'/exp OR 'antibiotic therapy'/exp OR 'antibiotic prophylaxis'/exp) AND ('controlled clinical trial'/de OR 'controlled study'/de OR 'randomized controlled trial'/de) AND ([adolescent]/lim OR [child]/lim OR [infant]/lim OR [newborn]/lim OR [preschool]/lim OR [school]/lim)

S2.1. Algorithm for Guidelines search

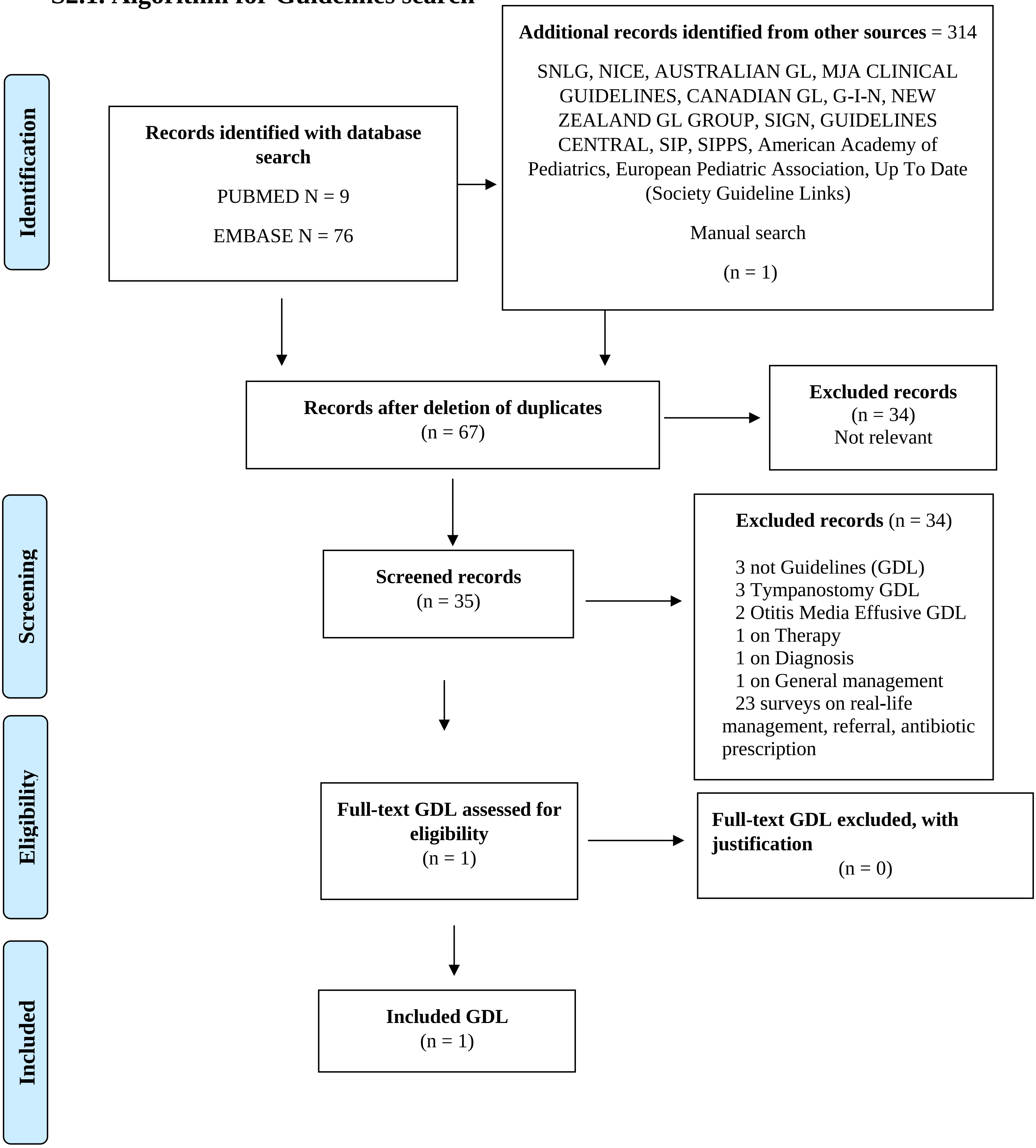

S2.2. Algorithm for Systematic Review (SR) search

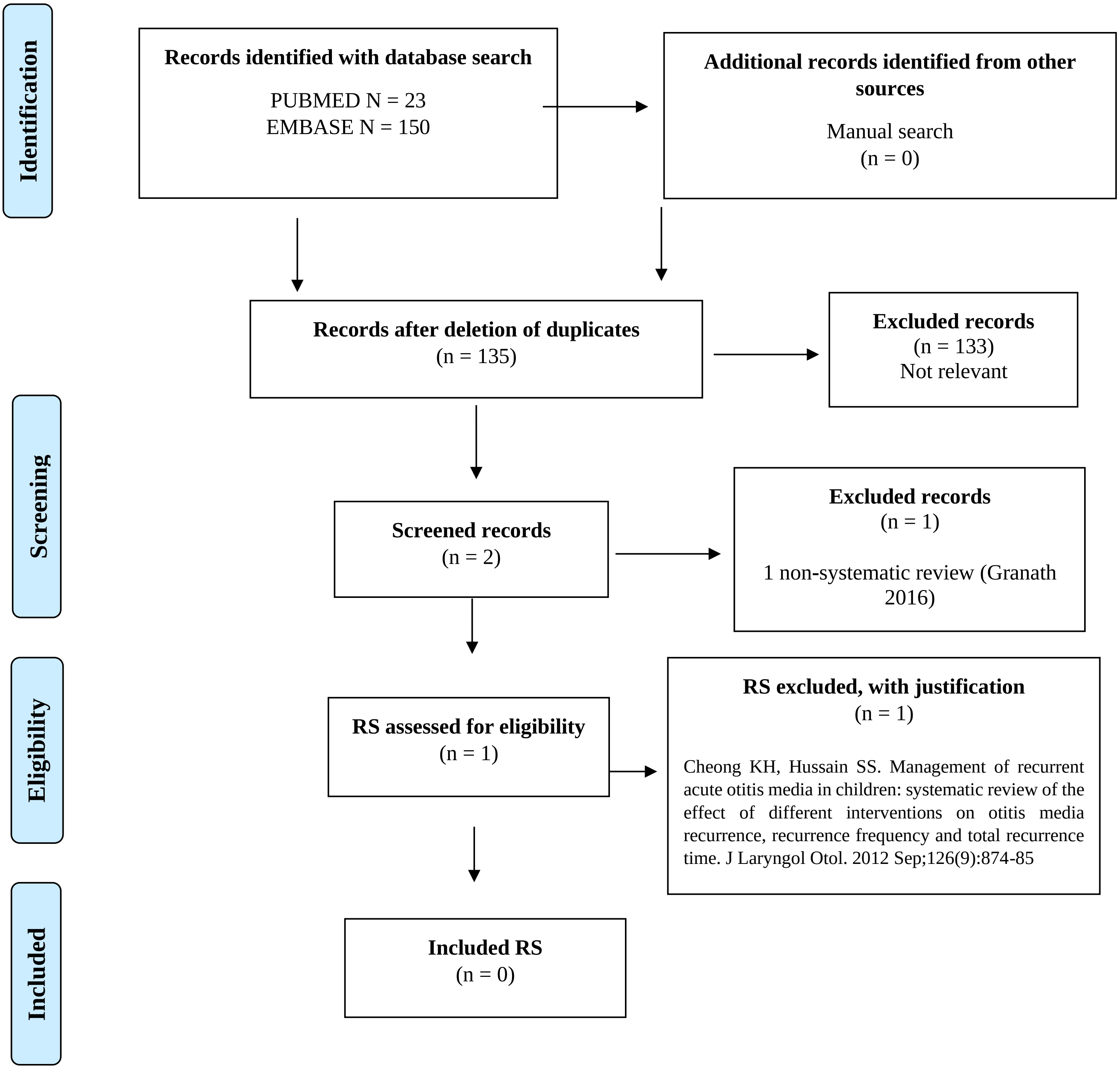

S2.3. Algorithm for Studies search

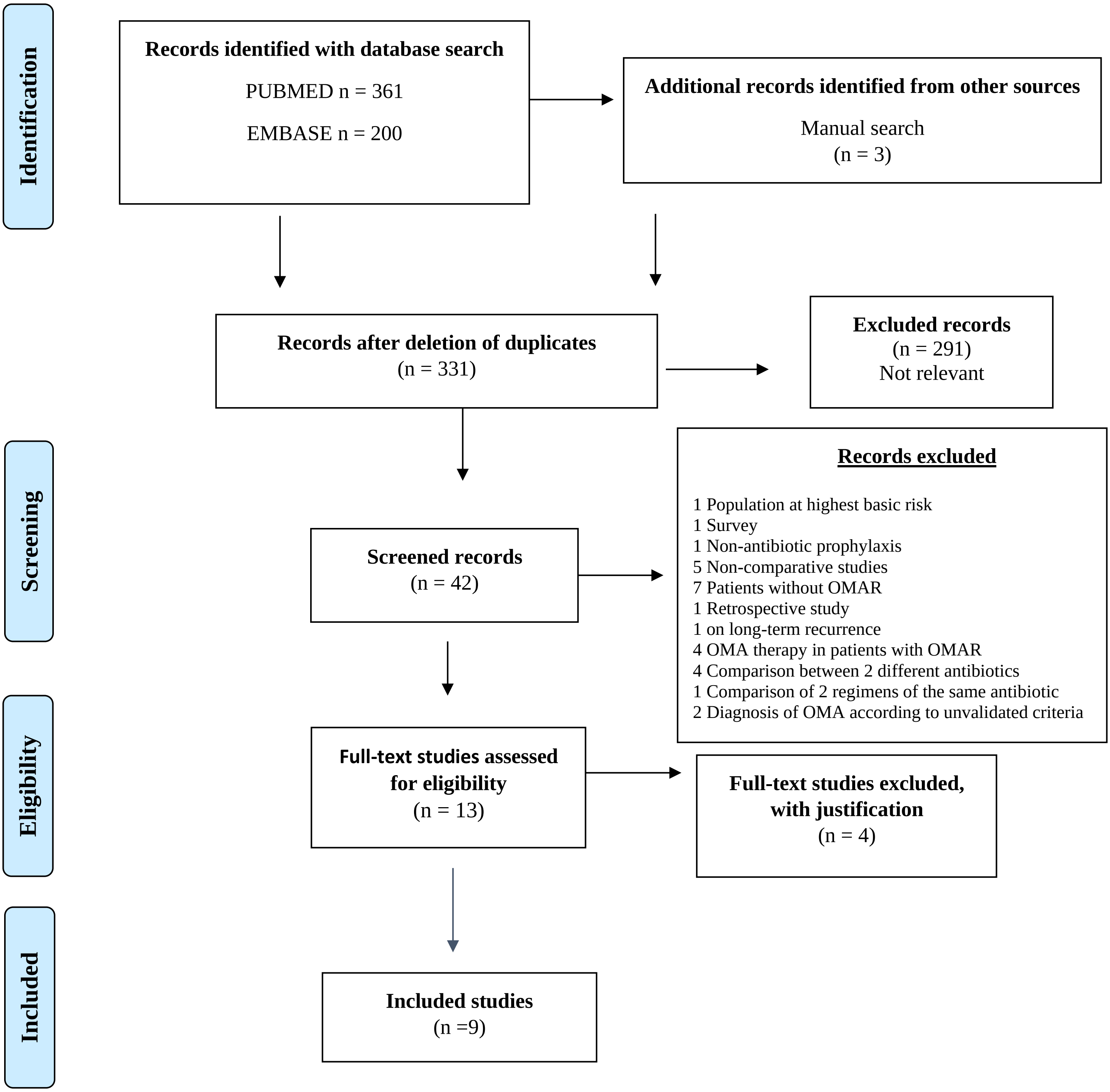

Supplement: Supplementary file 2 — Additional file 2. S2_RAOM Antibiotic prophylaxis GLs, SRs, Studies Search.pdf (PICOs, Keywords, Guidelines search, Systematic Review Search, Studies search, Algorithm for Guidelines search, Algorithm for Systematic Review search, Algorithm for Studies search). [file 13052_2025_1894_MOESM2_ESM.pdf]
