# Supplementary material for: Antibiotic treatment of acute and recurrent otitis media in children: an Italian intersociety Consensus
Source: Ital J Pediatr. 2025 Feb 20;51:50. doi: 10.1186/s13052-025-01894-z (PMC11844117; doi:10.1186/s13052-025-01894-z)
Supplement: Supplementary file 3 — Additional file 3. S3 S3_ AOM-RAOM_GLs, SRs, Studies Appraisal.pdf (methodological assessment). [file 13052_2025_1894_MOESM3_ESM.pdf]

S3. ACUTE OTITIS MEDIA - RECURRENT ACUTE OTITIS MEDIA

METHODOLOGICAL ASSESSMENT

Table S3.1. Appraisal of the Guidelines

**Guidelines**	**Methodological Appraisal AGREE II**		**Evaluators n = 2**
**NICE 2018**	**Score**		**Score**
**DOMAIN 1. SCOPE AND PURPOSE**	**92%**	**DOMAIN 3. RIGOUR OF DEVELOPMENT**	**79%**
1. The general objectives of the guideline are specifically described.	13	7. Systematic methods were used to search for evidence.	13
2. The health questions addressed by the guideline are specifically described.	12	8. The criteria for the selection of evidence are clearly described.	14
3. The population (patients, etc.) to which the guideline is to be applied is specifically described.	14	9. The strengths and limitations of the body of evidence are clearly described.	13
		10. The methods for formulating recommendations are clearly described.	13
**DOMAIN 2. USER INVOLVEMENT**	**92%**	11. Health benefits, side effects and risks were considered when formulating the recommendations.	12
4. The guideline development group includes individuals from all relevant professional groups.	14	12. There is an explicit link between recommendations and supporting evidence.	14
5. The opinions and preferences of the target population (patients, etc.) were sought.	12	13. The guideline was subject to external expert review prior to its publication.	2
6. The target users of the guideline are clearly defined.	13	14. A procedure for updating the guidelines is provided.	11
**DOMAIN 4. CLARITY OF PRESENTATION**	**64%**	**DOMAIN 5. APPLICABILITY**	**85%**
15. The recommendations are specific and unambiguous.	8	18. The guideline describes facilitating factors and obstacles to its implementation	12
16. The different options for managing the health condition or problem are clearly presented.	8	19. The guideline provides advice and/or tools on how to put the recommendations into practice.	13
17. The key recommendations are easily identified.	13	20. The potential resource implications of implementing the recommendations were considered.	13
		21. The guideline presents criteria for monitoring and/or auditing.	11
**DOMAIN 6. EDITORIAL INDEPENDENCE**	**88%**		

22. The views of the funder did not influence the content of the guideline.	12
23. Conflicts of interest of the members of the guideline development group were recorded and addressed.	13

**TOTAL SCORE**	**83%**
**Would you recommend the use of this guideline?**	**YES**<br>**Good methodological quality**

Guidelines	Methodological Appraisal AGREE II	Evaluators n = 2

SIP 2019	Score
**DOMAIN 1. SCOPE AND PURPOSE**	**86%**
1. The general objectives of the guideline are specifically described.	12
2. The health questions addressed by the guideline are specifically described.	12
3. The population (patients, etc.) to which the guideline is to be applied is specifically described.	13

**DOMAIN 2. USER INVOLVEMENT**	**78%**
4. The guideline development group includes individuals from all relevant professional groups.	13
5. The opinions and preferences of the target population (patients, etc.) were sought.	12
6. The target users of the guideline are clearly defined.	9

	Punteggio
**DOMAIN 3. RIGOUR OF DEVELOPMENT**	**61%**
7. Systematic methods were used to search for evidence.	7
8. The criteria for the selection of evidence are clearly described.	8
9. The strengths and limitations of the body of evidence are clearly described.	6
10. The methods for formulating recommendations are clearly described.	13
11. Health benefits, side effects and risks were considered when formulating the recommendations.	10
12. There is an explicit link between recommendations and supporting evidence.	5
13. The guideline was subject to external expert review prior to its publication.	13
14. A procedure for updating the guidelines is provided.	13

**DOMAIN 4. CLARITY OF PRESENTATION**	**81%**
15. The recommendations are specific and unambiguous.	12
16. The different options for managing the health condition or problem are clearly presented.	10
17. The key recommendations are easily identified.	13

**DOMAIN 5. APPLICABILITY**	**54%**
18. The guideline describes facilitating factors and obstacles to its implementation	7
19. The guideline provides advice and/or tools on how to put the recommendations into practice.	11
20. The potential resource implications of implementing the recommendations were considered.	7
21. The guideline presents criteria for monitoring and/or auditing.	9

**DOMAIN 6. EDITORIAL INDEPENDENCE**	**92%**
22. The views of the funder did not influence the content of the guideline.	13
23. Conflicts of interest of the members of the guideline development group were recorded and addressed.	13

**TOTAL SCORE**	**58%**
**Would you recommend the use of this guideline?**	**YES with modifications**

			**Moderate quality**

Table S3.2. Excluded Guidelines with reason

**Excluded Guidelines**	**Reason for exclusion**
Leach et al. 2021	Guideline on remote population at very high risk

Table S3.3. Appraisal of the Systematic Reviews

**AMSTAR 2**	Cheong et al. 2012
**1. Did the search questions and inclusion criteria for the review include PICO components? (Yes No)**	NO
**2. Did the report of the SR contain an explicit statement that the review methods were established before the review was conducted and did the report justify any significant deviations from the protocol? (Yes / Partially Yes / No)**	NO
**3. Did the authors of the review justify their selection of study designs for inclusion in the review? (Yes No)**	YES
**4. Did the review authors use a comprehensive literature search strategy? (Yes / Partially Yes / No)**	NO
**5. Did the authors of the review carry out the selection of duplicate studies? (Yes No)**	NO
**6. Did the review authors perform duplicate data extraction? (Yes No)**	NO
**7. Did the authors of the review provide a list of excluded studies and did they justify the exclusions? (Yes / Partially Yes / No)**	YES
**8. Did the authors describe the included studies in sufficient detail? (Yes / Partially Yes / No)**	NO
**9. Did the authors use a satisfactory technique to assess the risk of bias (RoB) in the individual studies included in the SR? (Yes / Partially Yes / No / It includes NRSI-RCT only)**	NO
**10. Did the authors report the funding sources of the studies included in the review? (Yes No)**	NO
**11. If a meta-analysis was performed, did the authors use appropriate methods to statistically consolidate the results? (Yes / No / No meta-analysis performed)**	No meta-analysis performed
**12. If meta-analysis was performed, did the authors assess the potential impact of RoB in individual studies**	No meta-analysis performed

**on the results of the meta-analysis or other evidence synthesis? (Yes / No / No meta-analysis conducted)**	
**13. Did the authors take the RoB into account in the individual studies when interpreting/discussing the results of the review? (Yes No)**	NO
**14. Did the authors provide a satisfactory explanation and discuss any heterogeneity observed in the review results? (Yes No)**	NO
**15. If they performed a quantitative synthesis, did the authors adequately search for publication bias (small study bias) and discuss its likely impact on the review results? (Yes / No / No meta-analysis performed)**	No meta-analysis performed
**16. Did the authors report potential sources of conflict of interest, including any funding received for conducting the review? (Yes No)**	YES
**OVERALL ASSESSMENT**	**CRITICALLY LOW QUALITY***
**CRITICAL DOMAINS AMSTAR 2**<br>Protocol recorded before the start of the review (item 2)<br>Adequacy of the bibliographic search (item 4)<br>Reason for exclusion of individual studies (item 7)<br>Risk of bias from individual studies included in the review (item 9)<br>Appropriateness of meta-analytical methods (item 11)<br>Consideration of the risk of bias in the interpretation of the result of the review (item 13)<br>Assessment of the presence and likely effect of publication bias (item 15)	***** Presence of 3 critical items (n. 2, 4, 15) and 7 failed non-critical items (n. 1, 5, 6, 8, 9, 10, 14)

Table S3.4. SRs excluded with reasons

SRs excluded	Reason for exclusion
**AOM questions**	
Djabali et al 2022	Abstract only for meeting. Full text not available
Dawit et al. 2021	Not relevant.<br>The comparison is with amoxicillin-clavulanate and not with amoxicillin alone as required in question 3.<br>The selection of patients: includes all OM, not just AOM, so also effusive and recurrent.
Holm et al. 2020	Not relevant as the comparison is between antibiotic (ATB) or placebo, not between ATB immediately and 'watchful waiting'. In any case, it only includes work after the 2018 NICE LGs (i.e. Venekamp et al. Cochrane 2015 SR): Rouhola et al. 2017, a study that is not relevant as it only examines the efficacy of ATB on EOM persistence (at 3 months).
Spurling et al. 2017	Update of the 2013 Cochrane SR by the same group of authors. It is not relevant as it includes only one new RCT (De la Poza Abad JAMA 2016;176(1):21-9) conducted in adults, so it does not contribute to the update of recommendations.
**Question 9 – RAOM**	
Cheong et al. 2012	Low methodological quality

Table S3.5. Appraisal of the Non-randomized intervention studies

**ROBINS-I tool (Stage II)**	Liston et al. 1994

Specify a target randomized trial specific to the study

Design	Individually controlled Matched
Participants	26 patients with RAOM enrolled consecutively to receive prophylaxis with co-trimoxazole + 26 children with RAOM enrolled with randomization who did not receive prophylaxis
Experimental intervention	Prophylaxis with cotrimoxazole for 13 weeks
Comparator	Placebo

Is your aim for this study…?

☐**X**	to assess the effect of *assignment to* intervention

☐	to assess the effect of *starting and adhering to* intervention

Specify the outcome

Specify which outcome is being assessed for risk of bias (typically from among those earmarked for the Summary of Findings table). Specify whether this is a proposed benefit or harm of intervention.

Episodes of AOM (benefit)

Specify the numerical result being assessed

In case of multiple alternative analyses being presented, specify the numeric result (e.g. RR = 1.52 (95% CI 0.83 to 2.77) and/or a reference (e.g. to a table, figure or paragraph) that uniquely defines the result being assessed.

**(ii) Additional confounding domains relevant to the setting of this particular study, or which the study authors identified as important**				
Confounding domain	Measured variable(s)	Is there evidence that controlling for this variable was unnecessary?*	Is the confounding domain measured validly and reliably by this variable (or these variables)?	OPTIONAL: Is failure to adjust for this variable (alone) expected to favour the experimental intervention or the comparator?
N. of AOM episodes in the previous 3 months			Yes / No / No information	Favour experimental / Favour comparator / No information
Placement in the community			Yes / No / No information	Favour experimental / Favour comparator / No information
Atopy			Yes / No / No information	Favour experimental / Favour comparator / No information
Age			Yes / No / No information	Favour experimental / Favour comparator / No information
Sex			Yes / No / No information	Favour experimental / Favour comparator / No information

**(ii) Additional co-interventions relevant to the setting of this particular study, or which the study authors identified as important**		
Co-intervention	Is there evidence that controlling for this co-intervention was unnecessary (e.g. because it was not administered)?	Is presence of this co-intervention likely to favour outcomes in the experimental intervention or the comparator
None		Favour experimental / Favour comparator / No information

Risk of bias assessment

Responses underlined in green are potential markers for low risk of bias, and responses in red are potential markers for a risk of bias. Where questions relate only to sign posts to other questions, no formatting is used.

Signalling questions	Description	Response options
**Bias due to confounding**		
1.1 Is there potential for confounding of the effect of intervention in this study?<br>**If N/PN to 1.1:** the study can be considered to be at low risk of bias due to confounding and no further signalling questions need be considered		Y / PY / PN / N
**If Y/PY to 1.1**: determine whether there is a need to assess time-varying confounding:		
1.2. Was the analysis based on splitting participants' follow up time according to intervention received?<br>**If N/PN**, answer questions relating to baseline confounding (1.4 to 1.6)<br>**If Y/PY**, go to question 1.3.		NA / Y / PY / PN / N / NI
1.3. Were intervention discontinuations or switches likely to be related to factors that are prognostic for the outcome?<br>**If N/PN**, answer questions relating to baseline confounding (1.4 to 1.6)<br>**If Y/PY**, answer questions relating to both baseline and time-varying confounding (1.7 and 1.8)		NA / Y / PY / PN / N / NI
**Questions relating to baseline confounding only**		
1.4. Did the authors use an appropriate analysis method that controlled for all the important confounding domains?		NA / Y / PY / PN / N / NI
1.5. **If Y/PY to 1.4**: Were confounding domains that were controlled for measured validly and reliably by the variables available in this study?		NA / Y / PY / PN / N / NI
1.6. Did the authors control for any post-intervention variables that could have been affected by the intervention?		NA / Y / PY / PN / N / NI
**Questions relating to baseline and time-varying confounding**		
1.7. Did the authors use an appropriate analysis method that controlled for all the important confounding domains and for time-varying confounding?		NA / Y / PY / PN / N / NI
1.8. **If Y/PY to 1.7**: Were confounding domains that were controlled for measured validly and reliably by the variables available in this study?		NA / Y / PY / PN / N / NI
**Risk of bias judgement**		Low / Moderate / Serious / Critical / NI

Optional: What is the predicted direction of bias due to confounding?		Favours experimental / Favours comparator / Unpredictable

Bias in selection of participants into the study		
2.1. Was selection of participants into the study (or into the analysis) based on participant characteristics observed after the start of intervention?<br>**If N/PN to 2.1:** go to 2.4		Y / PY / PN / N / NI
2.2. **If Y/PY to 2.1**: Were the post-intervention variables that influenced selection likely to be associated with intervention?		NA / Y / PY / PN / N / NI
2.3 **If Y/PY to 2.2**: Were the post-intervention variables that influenced selection likely to be influenced by the outcome or a cause of the outcome?		NA / Y / PY / PN / N / NI
2.4. Do start of follow-up and start of intervention coincide for most participants?		Y / PY / PN / N / NI
2.5. **If Y/PY to 2.2 and 2.3, or N/PN to 2.4**: Were adjustment techniques used that are likely to correct for the presence of selection biases?		NA / Y / PY / PN / N / NI
**Risk of bias judgement**		Low / Moderate / Serious / Critical / NI
Optional: What is the predicted direction of bias due to selection of participants into the study?		Favours experimental / Favours comparator / Towards null /Away from null / Unpredictable

Bias in classification of interventions		
3.1 Were intervention groups clearly defined?		Y / PY / PN / N / NI
3.2 Was the information used to define intervention groups recorded at the start of the intervention?		Y / PY / PN / N / NI
3.3 Could classification of intervention status have been affected by knowledge of the outcome or risk of the outcome?		Y / PY / PN / N / NI
**Risk of bias judgement**		Low / Moderate / Serious / Critical / NI
Optional: What is the predicted direction of bias due to classification of interventions?		Favours experimental / Favours comparator / Towards null /Away from null / Unpredictable

Bias due to deviations from intended interventions		
**If your aim for this study is to assess the effect of assignment to intervention, answer questions 4.1 and 4.2**		
4.1. Were there deviations from the intended intervention beyond what would be expected in usual practice?		Y / PY / PN / N / NI
4.2. **If Y/PY to 4.1**: Were these deviations from intended intervention unbalanced between groups *and* likely to have affected the outcome?		NA / Y / PY / PN / N / NI
**If your aim for this study is to assess the effect of starting and adhering to intervention, answer questions 4.3 to 4.6**		

4.3. Were important co-interventions balanced across intervention groups?		Y / PY / PN / N / NI
4.4. Was the intervention implemented successfully for most participants?		Y / PY / PN / N / NI
4.5. Did study participants adhere to the assigned intervention regimen?		Y / PY / PN / N / NI
4.6. **If N/PN to 4.3, 4.4 or 4.5**: Was an appropriate analysis used to estimate the effect of starting and adhering to the intervention?		NA / Y / PY / PN / N / NI
**Risk of bias judgement**		Low / Moderate / Serious / Critical / NI
Optional: What is the predicted direction of bias due to deviations from the intended interventions?		Favours experimental / Favours comparator / Towards null /Away from null / Unpredictable

**Bias due to missing data**		
5.1 Were outcome data available for all, or nearly all, participants?		Y / PY / PN / N / NI
5.2 Were participants excluded due to missing data on intervention status?		Y / PY / PN / N / NI
5.3 Were participants excluded due to missing data on other variables needed for the analysis?		Y / PY / PN / N / NI
5.4 **If PN/N to 5.1, or Y/PY to 5.2 or 5.3**: Are the proportion of participants and reasons for missing data similar across interventions?		NA / Y / PY / PN / N / NI
5.5 **If PN/N to 5.1, or Y/PY to 5.2 or 5.3**: Is there evidence that results were robust to the presence of missing data?		NA / Y / PY / PN / N / NI
**Risk of bias judgement**		Low / Moderate / Serious / Critical / NI
Optional: What is the predicted direction of bias due to missing data?		Favours experimental / Favours comparator / Towards null /Away from null / Unpredictable

**Bias in measurement of outcomes**		
6.1 Could the outcome measure have been influenced by knowledge of the intervention received?	La mancanza di cecità poteva influire poco sulla diagnosi di AOM da parte del ricercatore, ma poteva influire sui genitori per il sospetto di AOM in caso di sintomi lievi	Y / PY / PN / N / NI
6.2 Were outcome assessors aware of the intervention received by study participants?		Y / PY / PN / N / NI
6.3 Were the methods of outcome assessment comparable across intervention groups?		Y / PY / PN / N / NI
6.4 Were any systematic errors in measurement of the outcome related to intervention received?		Y / PY / PN / N / NI
**Risk of bias judgement**		Low / Moderate / Serious / Critical / NI

Optional: What is the predicted direction of bias due to measurement of outcomes?		Favours experimental / Favours comparator / Towards null /Away from null / Unpredictable

Bias in selection of the reported result		
Is the reported effect estimate likely to be selected, on the basis of the results, from...<br>7.1. ... multiple outcome *measurements* within the outcome domain?		Y / PY / PN / N / NI
7.2 ... multiple *analyses* of the intervention-outcome relationship?		Y / PY / PN / N / NI
7.3 ... different *subgroups*?		Y / PY / PN / N / NI
**Risk of bias judgement**		Low / Moderate / Serious / Critical / NI
Optional: What is the predicted direction of bias due to selection of the reported result?		Favours experimental / Favours comparator / Towards null /Away from null / Unpredictable

Overall bias		
**Risk of bias judgement**		Low / Moderate / Serious / Critical / NI
Optional: What is the overall predicted direction of bias for this outcome?		Favours experimental / Favours comparator / Towards null /Away from null / Unpredictable

This work is licensed under a Creative Commons Attribution-NonCommercial-NoDerivatives 4.0 International License.

Randomised clinical trials (RCTs)

FigureS3.1. Risk of bias summary: review authors' judgements about each risk of bias item for each included study.

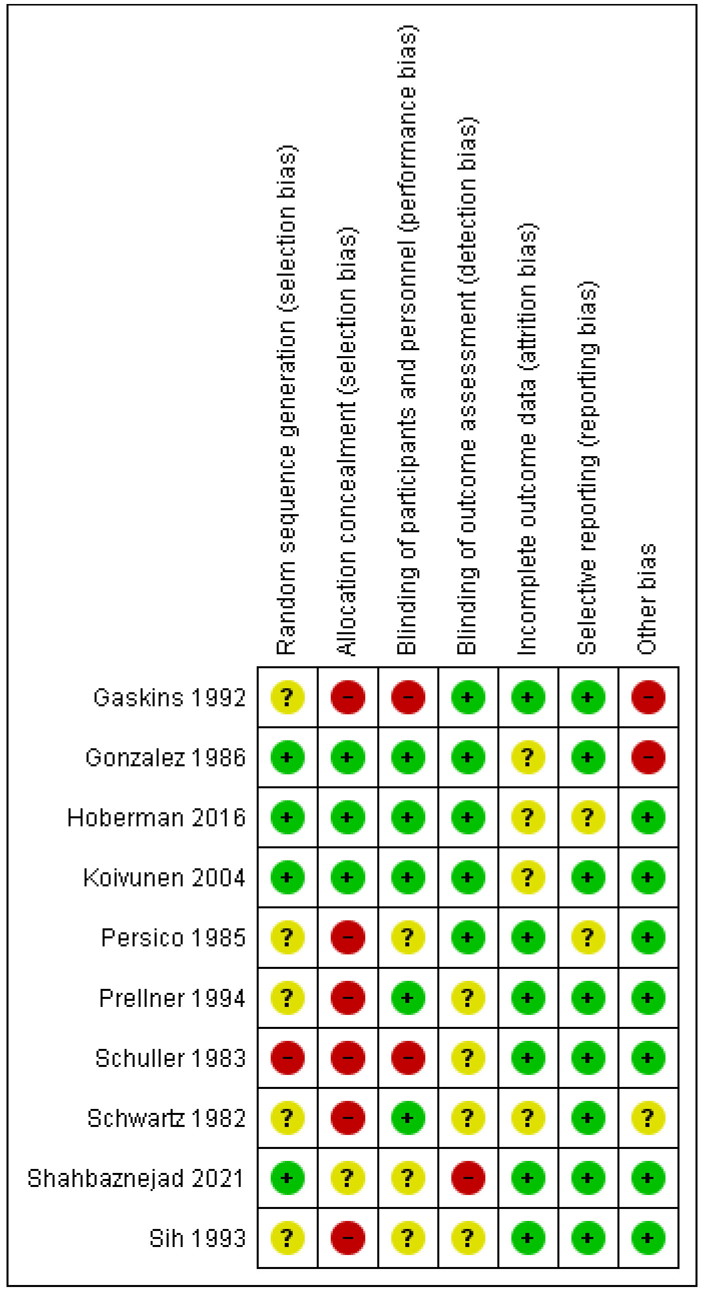

Figure S3.2. Risk of bias graph: review authors' judgements about each risk of bias item presented as percentages across all included studies.

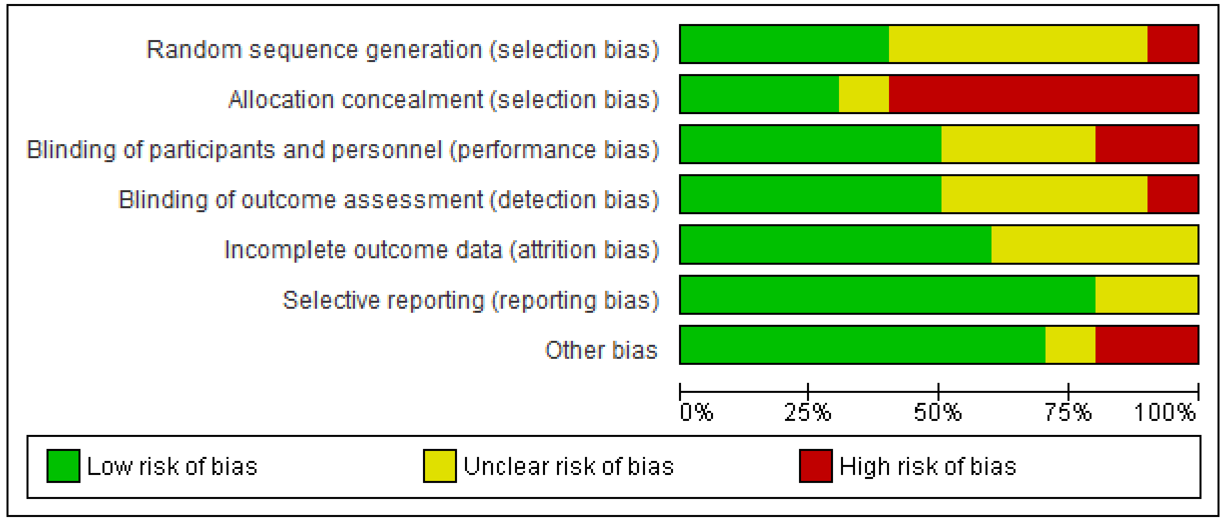

Table S3.6. Studies excluded with reason

**AOM questions**	
**Excluded studies**	**Reason for exclusion**
Ghosh et al. 2017	The comparisons do not correspond to PICOs
Hay et al. 2021	Not relevant. Comparisons do not correspond to PICOs
Hoberman et al 2017	Not relevant. It compares 2 dosages of clavulanic acid
Hullegie et al. 2021	Non relevant. Only protocol
Kono et al. 2017	Comparison with combination therapy, not between 2 antibiotics
Oliveira et al. 2021	Observational study. Only abstracts from Supplement
Ruohola et al. 2018	Not relevant. Study on the efficacy of ATB on OME persistence (at 3 months)
Tähtinen et al. 2017	Not relevant. The comparison is with no-therapy, not with Watchful Waiting
Uitti et al. 2016	Low quality. Uncontrolled study
**Question 9 - RAOM**	
Appelman et al. 1991	Patients without RAOM
Arguedas et al. 2003	Non-comparative study
Arrieta et al.2003	AOM therapy in patients with RAOM (not relevant to the question on prophylaxis)
Bezáková et al. 2009	Prospective study on the longtime effects of an ATB therapy for AOM
Block et al. 2001	Non-comparative study
Cardenas et al. 2018	Study on non-antibiotic prophylaxis

Casselbrant et al. 1992	Low methodological quality. Recurrence diagnoses could also be obtained by the mere finding of VET at follow-up visits. Loss to follow up > 20%.
Damoiseaux et al. 2006	Non-comparative study
De Diego et al.	Comparison of 2 antibiotics
Ellul et al. 2018	Non-comparative study
Fauskin et al. 1991	Non-comparative study
Fliss et al. 1989	Comparison of 2 antibiotics
Foglè-Hansson et al. 2001	Patients without RAOM
Gray et al. 1981	Congress report. Full text not available
Hampton et al. 2021	Survey, not trial
Jacobson et al. 1993	Patients without RAOM
Leach et al. 2008	Not relevant. To be excluded due to absence of diagnosis of RAOM
Mandel et al.1996	Low quality. They also include patients with persistent VET in the final analysis, considering them to be a new episode of AOM
Marchisio et al. 1996	Comparison of 2 regimens of the same antibiotic
Maynard et al. 1972.	Not applicable. Eskimo population with known high incidence
Noel et al. 2008	AOM therapy in patients with RAOM (not relevant to the question on prophylaxis)
Odio et al. 1995	Patients without RAOM
Perrin et al. 1974	Low quality. Cross-over study. Uncorrected diagnostic criteria for RAOM and AOM. Loss at follow-up > 20%.
Principi et al. 1989	Not applicable. Comparison of 2 antibiotics in RAOM prophylaxis
Roark et al. 1997	Low methodological quality. Persistence of VET is considered as a new episode of AOM
Roos et al. 2000	Comparison of 2 regimens of the same antibiotic

Saez-Llorens et al. 2005	AOM therapy in patients with RAOM (not relevant to the question on prophylaxis)
Salah et al. 2013	Retrospective study
Sher et al. 2005	AOM therapy in patients with RAOM (not relevant to the question on prophylaxis)
Te Molder et al. 2016	Study on patients without RAOM
Teele et al. 2000	Patients without RAOM
Varsano et al. 1985	Cross-over study. Conflicting data, low numerosity
