# Supplementary material for: Antibiotic treatment of acute and recurrent otitis media in children: an Italian intersociety Consensus
Source: Ital J Pediatr. 2025 Feb 20;51:50. doi: 10.1186/s13052-025-01894-z (PMC11844117; doi:10.1186/s13052-025-01894-z)
Supplement: Supplementary file 4 — Additional file 4. S4_AOM-RAOM RESULTS.pdf (guidelines reccomendations, RSs and studies results). [file 13052_2025_1894_MOESM4_ESM.pdf]

**S4. ACUTE OTITIS MEDIA - RECURRENT ACUTE OTITIS MEDIA (AOM-RAOM)**

**GLs RECOMMENDATIONS, RSs AND STUDIES RESULTS**

**Table S4.1. NICE GL 2018 Outcomes and Priorities.**

**NICE guideline [NG91] 2018 - Evidence Review. Appendix B**

| | |
|---|---|
| a) Clinical outcomes such as:<br>- mortality<br>- infection cure rates (number or proportion of people with resolution of symptoms at a given time, incidence of treatment escalation)<br>- time to clinical cure (mean or median time to resolution of disease)<br>- reduction in symptoms (duration or severity)<br>- rate of complications with or without treatment<br>- safety, tolerability, and adverse effects<br>b) Thresholds or indications for antimicrobial treatment (which people are more or less likely to benefit from antimicrobials)<br>c) Changes in patterns, trends, and levels of antimicrobial resistance following treatment<br>d) Patient-reported outcomes, such as medication adherence, patient experience, and patient satisfaction<br>e) Ability to perform activities of daily living<br>f) Service user experience<br>g) Health and social care-related quality of life, including long-term harm or disability<br>h) Health and social care utilization (including length of stay, stay at ITU, scheduled and unscheduled contacts) | The committee agreed that the following outcomes are critical:<br><br>- symptom reduction (duration or severity), e.g., difference in time to substantial improvement<br>- time to clinical recovery (mean or median time to disease resolution)<br>- rate of complications (including mortality) with or without treatment, including treatment escalation<br>- health and social care utilization (including length of stay, stays at ITU, scheduled and unscheduled contacts).<br>- thresholds or indications for antimicrobial treatment (which people are more or less likely to benefit from antimicrobials) |
| | The committee agreed that the following outcomes are important:<br><br>- Patient-reported outcomes, such as medication adherence, patient experience<br>- - Changes in patterns, trends, and levels of antimicrobial resistance following treatment |

**Table S4.2. Strength of the Recommendation in the NICE 2018 GLs**

| |
|---|
| **Developing NICE guidelines: the manual**<br>**Process and methods**<br>Published: 31 October 2014<br>www.nice.org.uk/process/pmg20<br><br>**9.2 Wording the recommendations** |
| **Reflect the strength of the recommendation**<br>The "strength" of a recommendation (see section on interpreting evidence to make recommendations) should be reflected in the consistent wording of recommendations within and across guidelines.<br>In recommendations about activities or interventions that should (or should not) be offered, use directive language such as "offer" (or "not offer"), "recommend" or "ask."<br>In line with the principles of shared decision making, people can choose whether or not to accept what is offered or recommended to them.<br>If there is a closer balance between benefits and harms (activities or interventions that could be used), use 'consider'.<br>If there is a legal requirement to implement a recommendation, or the consequences of not following a recommendation are extremely serious, the recommendation should use 'shall' or 'shall not' and be phrased in the passive voice |

**Table S4.3. Antibiotic choice NICE GL 2018 recommendations**

**NICE guideline [NG91] 2018 - Table 1 Treatment for children and young people under 18 years of age**

| **Treatment** | **Choice, dosage and duration of therapy** |
|---|---|
| Oral antibiotic of first choice: Amoxicillin | Amoxicillin<br>1 month to 11 months, 125 mg three times daily for 5-7 days<br>From 1 year to 4 years, 250 mg three times daily for 5-7 days<br>From 5 years to 17 years, 500 mg three times daily for 5-7 days |
| First alternative choice for penicillin allergy or intolerance (not in pregnancy) | Clarithromycin:<br>1 month to 11 yr:<br>Under 8 kg, 7.5 mg/kg twice daily for 5-7 days<br>From 8 kg to 11 kg, 62.5 mg twice daily for 5-7 days<br>From 12 kg to 19 kg, 125 mg twice daily for 5-7 days<br>From 20 kg to 29 kg, 187.5 mg twice daily for 5-7 days<br>From 30 kg to 40 kg, 250 mg twice daily for 5-7 days<br>From 12 to 17, 250 mg to 500 mg twice daily for 5-7 days |
| First alternative choice for penicillin allergy in pregnancy | Erythromycin:<br>8 years to 17 years,<br>250 mg to 500 mg four times daily |

| | |
|---|---|
| | or 500 mg to 1,000 mg twice daily for 5 to 7 days<br><br>Erythromycin is preferred if a macrolide is needed in pregnancy, such as if there is a true allergy to penicillin and the benefits of antibiotic treatment outweigh the harms. See the *Medicines and Healthcare products Regulatory Agency (MHRA) Public Assessment Report on the safety of macrolide antibiotics in pregnancy.* |
| Second-choice oral antibiotic: Amoxicillin-clavulanic acid (worsening of symptoms after at least 2 to 3 days of first-choice therapy) | Amoxicillin-clavulanic:<br><br>1 month to 11 months, 0.25 ml/kg suspension 125/31 three times daily for 5 to 7 days<br>From 1 year to 5 years, 5 ml of 125/31 suspension three times daily or 0.25 ml/kg of 125/31 suspension three times daily for 5-7 days<br>From 6 years to 11 years, 5 ml of 250/62 suspension three times a day or 0.15 ml/kg of 250/62 suspension three times a day for 5-7 days<br>From 12 to 17 years, 250/125 mg or 500/125 mg three times a day for 5-7 days |
| Second alternative choice for penicillin allergy or intolerance | Consult local microbiologist |

| | |
|---|---|
| ***Question 1: Does the age of the child condition the type of treatment strategy to be adopted in case of AOM?*** | **P** In the child with an age of less than 24 months and a diagnosis of AOM<br>**I** the watchful waiting<br>**C** versus immediate therapy<br>**O1** delays the resolution of symptomatology?<br>**O2** modifies the risk of suppurative complications?<br>**O3** increases the risk of short-term recurrence?<br>**O4** increases the risk of RAOM |

**Table S4.4. Question 1. NICE GL 2018 Recommendations and Grading.**

| | |
|---|---|
| **Question source.**<br>NICE guideline [NG91] 2018 - Evidence Review. **Appendix E – H**<br><br>***NICE question*.**<br>**Which people are most likely to benefit from an antibiotic? [Subgroup analysis of antibiotics versus placebo. Children younger than 2 years]** | |
| **Source of Recommendations**<br>NICE guideline [NG91] 2018<br><br>**Children and young people who may be most likely to benefit from antibiotics (those of any age with otorrhea or those younger than 2 years with infection in both ears)**<br><br>**Recommendation 1.1.11**<br>Consider:<br>- no antibiotic prescription with warning (see recommendation 1.1.9),<br>- a back-up antibiotic prescription§ with warning (see recommendation 1.1.10) or<br>- an immediate antibiotic prescription (see recommendation 1.2.1 for choice of treatment), taking into account:<br>• evidence that acute complications such as mastoiditis are rare with or without antibiotics<br>• possible adverse effects of antibiotics, particularly diarrhoea and nausea. [2018]<br><br>The committee accepted the recommendations of the previous NICE guideline on upper airway infections that, for acute otitis media, a strategy of prescribing without antibiotics or a strategy of prescribing back-up antibiotics should be agreed, but that, depending on the clinical assessment of severity, immediate antibiotic prescribing may be considered for children under 2 years of age with bilateral infection or children of any age with otorrhea (discharge of secretion following perforation of the eardrum). For these subgroups, the committee agreed that an immediate antibiotic prescription could also be considered as an option, as antibiotics may be more likely to be useful in these subgroups. The committee discussed that an immediate antibiotic may be | **Grading**<br><br>1.1.11. Quality of the evidence moderate. Weak recommendation, in favour of intervention. |

| | |
|---|---|
| preferred to no antibiotic or a back-up antibiotic prescription in some children based on clinical judgement.<br><br>*§ Back-up (delayed) prescription.*<br>NICE Quality standard [QS121] Antimicrobial stewardship<br><br>*A back-up (delayed) prescription is a prescription (which may be post-dated) given to a patient or caregiver on the assumption that it will not be dispensed immediately, but in a few days if symptoms worsen.* | |

**Table S4.5. Question 1. Included studies: Characteristics and Results.**

| Study (first author, year, country) | Study design | Population (sample size, characteristics) | Test | Primary outcome | Secondary outcome | Treatment effect measure | Follow-up | Outcomes | Funding |
|---|---|---|---|---|---|---|---|---|---|
| Shahbaznejad et al. 2021<br>Iran | non-blinded randomised clinical trial | 396 patients<br>age: 6 months - 6 years<br>Diagnosis of AOM: acute onset of fever, tympanic membrane erythema and effusive otitis media).<br>Exclusion criteria: severe AOM (fever greater than 39 °C, irritability, otalgia), otorrhea, conjunctivitis, immunodeficiency, penicillin allergy<br>note: only 1.5% of children vaccinated against PCV. | Amoxicillin 80 mg/kg/day for 7-10 days<br>vs<br>watchful waiting (control group)<br>In case of pain and fever prescribed ibuprofen or paracetamol | AOM cure rate (defined as resolution within 72h of fever, irritability, otalgia). | / | Incidence of recovery from OMA, persistence of clinical signs of AOM (fever, otalgia, irritability...) and occurrence of adverse reactions<br>Recurrence of AOM and effusive otitis media after 1 and 3 months | Visit at 72 h<br>Visit at 1 and 3 months to assess AOM frequency and effusive otitis media | Recovery rate from AOM 73% in the amoxicillin-treated group vs. 44% in the control group<br>Recovery from AOM greater in the treated group in both the 6-23 months and 24 months-6 years groups<br>3-day otalgia present in 15% of cases in the amoxicillin-treated group vs 28% in the control group<br>Fever in 19% of cases in the treated group vs. 44% control group<br>Irritability in 10% of the treated group vs 19% of controls<br>Significant improvement in patients with unilateral or bilateral tympanic membrane erythema and/or effusive otitis media in children 2 years of age and older<br>- Unilateral erythema: present in 24% of the treated group vs 51% of the control group<br>- Bilateral erythema: present in 31% of the treated group vs 65% of the control group<br>- Unilateral effusive otitis media: 25% in the treated group vs 58% in the control group<br>- Bilateral effusive otitis media: 36% in the treated group vs 69% control group<br>No statistically significant differences in AOM recurrence at 1 month; greater recurrence between 1 and 3 months in the control group.<br>Higher occurrence of side effects in the intervention group (11% vs 3%), in particular diarrhoea (7% vs 2%). | Not declared |

| | |
|---|---|
| ***Question 2: The severity of the AOM episode influences the time to start antibiotic therapy?*** | **P1** In children diagnosed with AOM and fever >39°C<br>**P2** In children diagnosed with AOM and otorrhoea<br>**P3** In children diagnosed with AOM and general condition impairment<br>**P4** In the child diagnosed with bilateral AOM **I** the watchful waiting strategy<br>**C** compared to immediate antibiotic therapy<br>**O1** delays the resolution of symptoms?<br>**O2** modifies the risk of suppurative complications?<br>**O3** does it increase the risk of short-term recurrence?<br>**O4** does it increase the risk of RAOM? |

**Table S4.6. Question 2. NICE GL 2018 Recommendations and Grading.**

| | |
|---|---|
| **Question source.**<br>NICE guideline [NG91] 2018 Evidence Review **Appendix E - H**<br><br>***NICE question*.**<br>**Which people are most likely to benefit from an antibiotic?** | |
| **Source of Recommendations**<br>NICE guideline [NG91] 2018 | **Grading**<br><br>1.1.13. Strong, in favour of intervention |

| | |
|---|---|
| **Children and young people who are systematically very ill, have symptoms and signs of a more serious illness or condition or are at high risk of complications**<br><br>Rec. 1.1.13 Offer an immediate antibiotic prescription with warning.<br><br>Rec. 1.1.14 Refer children and young people to hospital if they have acute otitis media associated with:<br>- a severe systemic infection (see NICE guidelines on sepsis)<br>- acute complications including mastoiditis, meningitis, intracranial abscesses, sinus thrombosis or facial nerve palsy. [2018]<br><br>The committee agreed that the immediate prescription of antibiotics is important for children who have severe systemic symptoms, have symptoms or signs of more severe AOM, or are at high risk of serious complications due to a pre-existing comorbidity. This includes children with significant heart, lung, kidney, liver or neuromuscular disease, immunosuppression, cystic fibrosis and children born prematurely. | 1.1.14. Strong, in favour of intervention |

| | |
|---|---|
| ***Question 3: When antibiotic therapy is required, can amoxicillin still be considered the molecule of first choice in children with acute otitis media?*** | **P** In a child with AOM requiring antibiotic therapy<br>**I** the use of amoxicillin versus<br>**C1** amoxicillin/clavulanate<br>**C2** 2nd generation cephalosporins<br>**C3** 3rd generation cephalosporins<br>**C4** macrolides<br>**O1** reduces cure rate<br>**O2** modifies the risk of suppurative complications?<br>**O3** does it increase the risk of short-term recurrence?<br>**O4** does it increase the risk of RAOM? |

**Table S4.7. Question 3. NICE GL 2018 Recommendations and Grading**

| **Question source.**<br>NICE guideline [NG91] 2018 - Evidence Review **Appendix E - H**<br><br>***NICE question*.**<br>**Antibiotics versus different antibiotics** | |
|---|---|
| **Source of Recommendations**<br>NICE guideline [NG91] 2018<br><br>Recommendation 1.2.1 Follow the indications in Table 1. Oral antibiotic of first choice: Amoxicillin.<br><br>Recommendation based on:<br>- minimise the risk of resistance<br>- narrow-spectrum antibiotic should generally be the first choice<br>Based on the evidence on the absence of substantial differences in clinical efficacy between antibiotic classes, the committee agreed that the choice of antibiotic should largely be guided by minimising the risk of resistance.<br><br>The committee discussed that if an antibiotic is needed to treat an infection that is not life-threatening, a narrow-spectrum antibiotic should generally be the first choice. The indiscriminate use of broad-spectrum antibiotics creates a selective advantage for bacteria resistant to even these 'last-line' broad-spectrum agents and also kills the normal commensal flora, leaving people susceptible to harmful antibiotic-resistant bacteria such as Clostridium difficile. For infections that are not life-threatening, broad-spectrum antibiotics should be reserved for second-line treatment when narrow-spectrum antibiotics are ineffective.<br><br>Based on evidence, personal experience and resistance data, the committee agreed to recommend amoxicillin as first choice because this is current practice for antibiotic treatment in children with acute otitis media and the risk of resistance is acceptable | **Grading**<br><br>1.2.1. Quality of evidence moderate for penicillin vs. cephalosporins<br>Low for penicillin vs. macrolide (consistent evidence, expert opinion, resistance data)<br>Strong recommendation in favour of Amoxicillin |

| | |
|---|---|
| ***Question 4: Is the use of amoxicillin at a dose of 80-90 mg/Kg/day more effective than its use at a dose of 50mg/Kg/day?*** | **P** In children with AOM<br>**I** the use of amoxicillin at a dose of 80-90 mg/Kg/day<br>**C** compared to the dose of 50 mg/Kg/day<br>**O1** is equally effective in achieving cure?<br>**O2** does it alter the risk of suppurative complications?<br>**O3** does it increase the risk of short-term recurrence?<br>**O4** does it increase the risk of OMAR? |

**Table S4.8. Question 4. NICE GL 2018 Recommendations and Grading.**

| **Question source.**<br>NICE guideline [NG91] 2018 - Evidence Review **Appendix E - H**<br><br>***NICE question***.<br>**What is the optimal dosage, duration and route of administration of the antibiotic? (Dosage)** | |
|---|---|
| **Source of Recommendations**<br>NICE guideline [NG91] 2018<br><br>Recommendation 1.2.1 Follow the indications in Table 1. Oral antibiotic of first choice: Amoxicillin.<br>1 month to 11 months, 125 mg three times daily for 5-7 days<br>1 year to 4 years, 250 mg three times a day for 5-7 days<br>From 5 years to 17 years, 500 mg three times a day for 5-7 days<br><br>The dose of 125 mg to 500 mg three times a day (depending on age) is the usual dose and was similar to that used in the studies. The committee discussed that phenoxymethylpenicillin has a lower risk of resistance than amoxicillin and that microbiologically it should be equivalent. However, medication adherence is particularly important for children and acute otitis media occurs most commonly in young children. Amoxicillin is administered three times a day instead of four times a day like phenoxymethylpenicillin and the liquid formulation is more palatable. | **Grading**<br><br>1.2.1. Quality of the evidence low Expert opinion (no systematic literature review on this question). Strong recommendation in favour of intervention |

**Table S4.9. Question 4. SIP GL 2019 Recommendations and Grading.**

| **Question source.**<br>SIP GL 2019 - Treatment<br><br>***Question from the 2019 SIP GLs***<br>***Question 3. Which molecules are recommended for use in antibiotic therapy?*** | |
|---|---|
| **Source of Recommendations**<br>SIP GL 2019<br><br>Recommendation 7. In uncomplicated AOM with mild symptoms in children without risk factors for bacterial resistance and without a history of recurrence, amoxicillin is recommended at a dose of 80 - 90 mg/kg/day<br><br>Increasing the dose from 40-50 mg/kg/day to 80-90 mg/kg/day is associated with an increased amoxicillin concentration in the middle ear (Seikel, 1997), which ensures efficacy against most S. pneumoniae strains, including those with intermediate resistance (MIC ≥2 and <8 mcg /mL) (Weinstein 2009).<br>Highly penicillin-resistant S. pneumoniae strains (MIC ≥8 µg / mL), which represent, however, less than 2% of isolates, do not respond to high doses of amoxicillin (Lee 2017).<br>However, regarding optimal dosing, the efficacy data available in the literature are conflicting. ...<br>Guideline recommendations are also conflicting. The 2013 AAP guideline favours the use of high dosage (80-90 mg/kg/day) and this choice is also shared by other guidelines (Belgium, France, South Africa), while still others advocate lower dosages (e.g. the Netherlands, 30-40 mg/kg/day) (Damoiseaux 2006, Ovnat Tamir 2017). The discrepancy is attributable to the different epidemiological and resistance situations (Segal 2005b). In Italy, S. pneumoniae resistance to penicillin is 8%, supporting the use of high dosages (Emilia Romagna data 2016). | **Grading**<br><br>Strong recommendation in favour of intervention |

| | |
|---|---|
| ***Question 5: Should amoxicillin or amoxicillin/clavulanate therapy be divided into 2 or 3 daily doses?*** | **P** In the child with AOM<br>**I1** the fractionation of amoxicillin therapy into two daily doses<br>**I2** the fractionation of amoxicillin/clavulanate therapy into two daily doses<br>**C** compared to fractionation into three daily doses<br>**O1** Does it increase therapeutic adherence?<br>**O2** Does it increase the risk of treatment failure?<br>**O3** Does it increase the risk of suppurative complications?<br>**O4** Does it increase the risk of recurrence in the short term?<br>**O5** Does it increase the risk of RAOM? |

**Table S4.10. Question 5. NICE GL 2018 Recommendations and Grading.**

| | |
|---|---|
| **Question source.**<br>NICE guideline [NG91] 2018 Evidence Review Appendix E – H<br><br>***NICE question***<br>***What is the optimal dosage, duration, and route of administration of the antibiotic? (Dosage)*** | |
| **Source of Recommendations**<br>NICE guideline [NG91] 2018<br><br>Recommendation 1.2.1 Follow the directions in Table 1.<br><br>First choice oral antibiotic: Amoxicillin.<br>1 month to 11 months, 125 mg three times daily for 5 to 7 days<br>1 year to 4 years, 250 mg three times daily for 5 to 7 days<br>5 years to 17 years, 500 mg three times daily for 5 to 7 days<br><br>Second choice oral antibiotic: Amoxicillin-clavulanic (worsening of symptoms after at least 2 to 3 days of first-choice therapy)<br>1 month to 11 months, 0.25 ml/kg 125/31 suspension three times daily for 5-7 days<br>1 year to 5 years, 5 mL of 125/31 suspension three times a day or 0.25 mL/kg of 125/31 suspension three times a day for 5-7 days<br>6 years to 11 years, 5 mL of 250/62 suspension three times daily or 0.15 mL/kg of 250/62 suspension three times daily for 5 to 7 days<br>12 to 17 years, 250/125 mg or 500/125 mg three times daily for 5 to 7 days<br><br>The Committee agreed that, when prescribing amoxicillin or co-amoxiclav, a frequency of administration of three times a day should be prescribed, as is current practice. [2018] The evidence to support once or twice daily dosing is for different doses and longer durations of treatment. This goes against the general principle of antimicrobial stewardship | **Grading**<br><br>1.2.1. Low quality of evidence Expert opinion (inconsistent literature data). Weak recommendation, in favor of intervention (3 doses) |

| | |
|---|---|
| ***Question 6: Is a duration of antibiotic therapy of 5 days or 10 days more effective in the child with AOM?*** | **P1** In the child with AOM and age < 24 months<br>**P2** In the child with AOM and age ≥ 24 months<br>**I1** a duration of antibiotic therapy of 5 days<br>**I2** a duration of antibiotic therapy of 7 days<br>**C** compared to a duration longer than 10 days<br>**O1** Does it increase therapeutic adherence?<br>**O2** Does it increase the risk of treatment failure?<br>**O3** Does it increase the risk of suppurative complications?<br>**O4** Does it increase the risk of recurrence in the short term?<br>**O5** Does it increase the risk of RAOM?<br>**O6** Does it reduce the risk of adverse events (allergic/urticarial reactions, antibiotic diarrhea,..)? |

**Table S4.11. Question 6. NICE GL 2018 Recommendations and Grading.**

| | |
|---|---|
| **Question source.**<br>NICE guideline [NG91] 2018 Evidence Review Appendix E – H<br><br>***NICE question***<br>***What is the optimal dosage, duration, and route of administration of the antibiotic? (Duration of treatment)*** | |
| **Source of Recommendations**<br>NICE guideline [NG91] 2018<br><br>Recommendation 1.2.1 Follow the directions in Table 1. First choice oral antibiotic: Amoxicillin.<br><br>1 month to 11 months, 125 mg three times daily for 5 to 7 days<br>1 year to 4 years, 250 mg three times daily for 5 to 7 days<br>5 years to 17 years, 500 mg three times daily for 5 to 7 days<br><br>Second choice oral antibiotic: Amoxicillin-clavulanic (worsening of symptoms after at least 2 to 3 days of first-choice therapy)<br><br>1 month to 11 months, 0.25 ml/kg 125/31 suspension three times daily for 5-7 days<br>1 year to 5 years, 5 mL of 125/31 suspension three times a day or 0.25 mL/kg of 125/31 suspension three times a day for 5-7 days<br>6 years to 11 years, 5 mL of 250/62 suspension three times daily or 0.15 mL/kg of 250/62 suspension three times daily for 5 to 7 days<br>12 to 17 years, 250/125 mg or 500/125 mg three times daily for 5 to 7 days | **Grading**<br><br>1.2.1. Low quality of evidence. Weak recommendation, in favor of intervention (3 doses) |

| | |
|---|---|
| Based on the evidence, their experience and resistance data, the committee agreed that a 5-7 day course of all recommended antibiotics is sufficient to treat acute otitis media in children. This takes into account both the evidence of clinical efficacy and the evidence of the safety and tolerability of antibiotics and minimizes the risk of resistance. Studies on the use of specific antibiotics for the treatment of acute otitis media have sometimes lasted longer than 7 days. [2018]<br>The committee noted that no studies were identified which directly compared a 5-day antibiotic cycle with a 7-day cycle. [2018]<br><br>Based on evidence, the committee recognized that more children may have a therapeutic failure with an antibiotic cycle less than 7 days compared to a 7-day or longer cycle. However, the absolute difference is small. From 8 to 19 days, 82% of children who took antibiotics for less than 7 days were better, compared with 86% of those who took antibiotics for 7 days or more. They agreed that if the decision is made to prescribe an antibiotic, a 5-day course may be sufficient for many children, reserving 7-day courses for those with a more severe or recurrent clinical assessment of infection. [2018] | |

**Table S4.12. Question 6. SIP GL 2019 Recommendations and Grading.**

| | |
|---|---|
| **Question source.**<br>SIP GL 2019 - Treatment<br><br>***Question from the 2019 SIP GLs***<br>***Question 5. What is the optimal duration of antibiotic therapy?*** | |
| **Source of Recommendations**<br>SIP GL 2019<br><br>Recommendation 11. The duration of antibiotic therapy with amoxicillin or amoxicillin-clavulanic acid should be 10 days in children at risk of adverse evolution (under 2 years and/ or with otorrhea spontaneous)<br><br>Recommendation 12. Duration may be reduced to 5 days in children without risk of adverse evolution (children aged over 2 years, without otorrhea, bilaterality and without serious symptomatology)<br><br>The Cochrane systematic review of Kozyrskyj in 2010 including 49 studies (N= 12045 patients under 18 years old) found that the risk of failure (understood as the absence of clinical resolution, recovery or recurrence of OMA one month after starting therapy) was overall higher in patients treated for less than 7 days compared to those treated with antibiotics for more than a week (21% versus 18%; OR:1.34; 95% CI 1.15-1.55) (Kozyrskyj 2010).<br>In children under two years of age, a randomized clinical trial by Hoberman et al., of good quality, compared the efficacy of amoxicillin clavulanate therapy for 10 days with a 5-day regimen in 520 children. Children treated with antibiotics for 5 days had a higher risk of clinical failure than those treated for 10 days (34% versus 16%; P=0.02); this difference increased further in those with bilateral OMA (P<0.001). Clinically, the percentage of children with symptom reduction was lower in the group treated with a short course than in the group treated for 10 days (80% vs. 91%; P= 0.003). | **Grading**<br><br>11. Strong recommendation for intervention<br><br>12. Weak recommendation in favor of intervention |

**Table S4.13. Included studies: Characteristics and Results.**

| Study (first author, year, country) | Study design | Population (sample size, characteristics) | Intervention / Exposure | Primary outcome | Secondary outcome | Measurement of treatment effect | Follow-up | Outcomes | Funding |
|---|---|---|---|---|---|---|---|---|---|
| Hoberman A, et al. 2016 USA | RCT double blind non inferiority study design | 467 pcs<br><br>age: 6-23 months<br><br>Inclusion criteria: AOM diagnosis, with at least 2 anti doses - PCV<br><br>Exclusion criteria: perforation - allergy amoxicillin - antibiotic in previous 96h | 10 days amoxicillin - clavulanic acid vs 5 days amoxicillin-ac.clavulanic + 5 days placebo<br><br>dose amoxicillin: 90 mg + 6 mg clavulanate/ per kg | Treatment failure in 2 groups | Recurrence of otitis media | Primary measure: % of children with clinical failure after infection treatment index<br><br>Secondary measures:<br>- symptoms from day 6 to day 14<br>- Rate of recurrence of otitis media<br><br>Outcomes in the treatment of acute otitis media relapses | Telephone conversation at day 4 or 5 or 6<br><br>Visit on day 12 or 13 or 14<br><br>Visit every 6 weeks until the end of winter season<br><br>End of study visit in September for subjects with first line failure: further post-treatment assessment | Highest failure rate in group 5gg (34%) vs 10 gg (16%)<br><br>Most symptoms at 6-14 days in the 5 day group compared to 10 days with AOM-SOS scores (P = 0.07); average score 12-14 days higher in the 5 day group (P = 0.001).<br><br>Lower rate of reduction of symptoms (reduction of more than 50%) in the group 5 days [80%] vs. [91%], P=0.003). | No commercial support. |

| | | | | | | | | | |
|---|---|---|---|---|---|---|---|---|---|
| | | | | | | | | Residual effusion in 62% 10-day group and 65% in 5-day group (P=0.35); relapse of acute otitis media among children with residual effusion higher than those without (48% vs. 29%, P<0.001) and among clinically successful children (45% vs. 29%, P = 0.01) both among those who had clinical failure (59% vs. 32%, P = 0.008).<br><br>Clinical failure rate for recurrence consistently higher in the 5-day group than in the 10-day group (28% versus 19%).<br><br>Adverse events: diarrhea 78 of 257 children (30%) in the 10-day group and 75 of 258 (29%) in the 5-day group; diaper dermatitis requiring a topical antifungal agent occurred in 85 of 257 (33%) and 87 of 258, respectively (34%) of the European Union. | |

| | |
|---|---|
| ***Question 7: What antibiotic is recommended for the child with AOM who does not heal or relapses in the short term after amoxicillin 80/90mg/kg/day therapy?*** | **P.** In the child with AOM that does not heal or relapses short term after amoxicillin therapy at 80-90mg/kg/day<br>**I1.** a 2nd generation cephalosporin therapy<br>**I2**. a 3rd generation cephalosporin therapy<br>**I3**. a macrolide therapy<br>**I4.** a therapy with quinolones<br>**C.** compared to amoxicillin/clavulanate therapy<br>**O1**. is more effective in achieving healing?<br>**O2**. is more effective in reducing the risk of short-term recurrence?<br>**O3**. is more effective in reducing the risk of RAOM?<br>**O4**. is more effective in reducing the risk of suppurative complications? |

**Table S4.14. Question 7. NICE GL 2018 Recommendations and Grading.**

| | |
|---|---|
| **Question source.**<br>NICE guideline [NG91] 2018 Evidence Review Appendix E – H<br><br>***NICE Question***<br>***What is the optimal dosage, duration and route of administration of the antibiotic??*** | |
| **Source of Recommendations**<br>NICE guideline [NG91] 2018<br><br>Recommendation 1.2.1. Recommendation 1.2.1 Follow the instructions in Table 1.<br>Second choice oral antibiotic: Amoxicillin-clavulanic<br>(worsening of symptoms after at least 2 or 3 days of first choice therapy)<br><br>Based on the evidence, their experience and resistance data, the committee agreed to recommend co-amoxiclav as a second-choice antibiotic to use if symptoms worsen with a first-choice antibiotic taken for at least 2 or 3 days. This broad-spectrum treatment combines a | **Grading**<br><br>1.2.1. Moderate quality of evidence (consistent evidence, expert opinion, resistance data). Strong recommendation, in favour of intervention |

| | |
|---|---|
| penicillin (amoxicillin) with a beta-lactamase inhibitor, making it active against the beta-lactamase producing bacteria that are resistant to amoxicillin alone. People who do not respond to amoxicillin may be more likely to have an infection that is resistant to it. | |

| | |
|---|---|
| ***Question 8: What is the first choice of antibiotic therapy for an episode of AOM in children with R AOM?*** | **P.** In the child with RAOM<br>**I.** antibiotic therapy with a different antibiotic<br>**C.** Compared to first choice therapy with amoxicillin antibiotic (or second choice antibiotic amoxicillin/clavulanate (worsening symptoms on first choice taken for at least 2 to 3 days)<br>**O1.** is more effective in achieving healing?<br>**O2.** is more effective in reducing the risk of short-term recurrence?<br>**O3.** is more effective in reducing the risk of RAOM?<br>**O4.** is more effective in reducing the risk of suppurative complications? |

**Table S4.15. Question 8. NICE GL 2018 Recommendations and Grading.**

| | |
|---|---|
| **Question source.**<br>NICE guideline [NG91] 2018 Evidence Review Appendix E – H<br><br>***NICE Question***<br>***Antibiotic choice. Antibiotics versus different antibiotics?*** | |
| **Source of Recommendations**<br>NICE guideline [NG91] 2018<br><br>Recommendation 1.2.1 Follow the instructions in Table 1.<br><br>Second choice oral antibiotic: Amoxicillin-clavulanic<br>(worsening of symptoms after at least 2 or 3 days of first choice therapy)<br><br>One RS (Shekelle et al. 2010) also considered evidence for the treatment of recurrent or persistent acute otitis media in children. None of the studies found a significant benefit in successful treatment with a particular antibiotic. | **Grading**<br><br>1.2.1. Quality of evidence moderate. Strong recommendation, in favor of intervention |

| | |
|---|---|
| ***Question 9: In children with RAOM, is antibiotic prophylaxis effective to reduce recurrence of episodes?*** | **P** In the child with RAOM treatment with:<br>**I1** Long-term antibiotic prophylaxis with amoxicillin or amoxicillin/clavulanate<br>**I2**Long-term antibiotic prophylaxis with other antibiotics (e.g. macrolides, cotrimoxazole)<br>**C** compared to antibiotic treatment of each episode<br>**O** is it more effective in reducing the recurrence of episodes? |

**Table S4.16. Question 9. SIP GL 2019 Recommendations and Grading.**

| Guideline | Recommendations | Grading |
|---|---|---|
| SIP GL 2019 | Recommendation 5. Antibiotic prophylaxis not recommended in the prevention of recurrent AOM except in selected cases<br><br>[Leach AJ, Morris PS. Antibiotics for the prevention of acute and chronic suppurative otitis media in children. Cochrane Database System Rev 2006;4:CD00440#<br><br>Cheong KH, Hussain SS. Management of recurrent acute otitis media in children: systematic review of the effect of different interventions on recurrence, frequency and total time of recurrence. Laryngol Otol 2012;126:874-85 ] | Weak recommendation against intervention |

**Table S4.17. Question 9. Included studies: Characteristics and Results.**

| Study (first author, year, country) | Study design | Population (sample size, characteristics) | Intervention / Exposure | Primary outcome | Secondary outcome | Measurement of effect | Follow-up | Outcomes | Funding |
|---|---|---|---|---|---|---|---|---|---|
| Gaskins et al., 1982<br><br>Porto Rico | RCT in open | 21 children (10 intervention group vs 11 control group)<br>Age = between 1 and 14 years with 3 or + episodes of AOM in the previous 18 months or 5 or + episodes in any time interval.<br>Criteria required for diagnosis of AOM:<br>-compatible symptomatology<br><br>+ at least:<br><br>-a tympanic (MT) erythematous membrane<br>-Outburst (bulging) of MT<br>-loss of TM reference points<br>-"abnormal findings" at the pneumatic otoscopy<br>-tympanocentesis with positive culture | Treatment of only acute vs prophylactic episode with TMP-SMX b.i.d from the 11th day after acute episode treatment for a total duration of 6 months. | Efficacy of daily prophylaxis with TMP-SMX for 6 months after acute event in the prevention of recurrent OMA in high risk children | / | Difference in incidence of AOM between the 2 groups over 6 months of observation | Visits at 6-week intervals. | No child in the study group had a relapse of AOM compared to 8 children in the control group (p<0.005).<br>No child has experienced any severe side effects that would cause them to be withdrawn from the study.<br>Nausea in 3 children only<br>An altered blood count was recorded in two cases | Not declared |
| Gonzalez et al.., 1986<br><br>USA | Prospective blind randomized study | 68 patients, with RAOM (defined by one or two episodes of AOM occurring in a 3-month period)<br>78% of the population <2aa<br>Three groups treated:<br>-20 with placebo<br>-21 with sulfisoxazole (500 mg x 2 times daily if <5aa, 1gr x twice daily > 5 aa)<br>-22 with tympanostomy tube (TT) | Treatment of R AOM with tympanostomy tube vs prophylaxis with sulfisoxazole vs placebo | Effectiveness of treatment with tympanostomy tube, prophylaxis antibiotic and placebo | / | Difference in number of ear infections per child in the first six months of observation (attack rate)<br>Treatment failure considered as two/three episodes of OMA between the 3 groups in less than three months | six months of follow-up | 40 episodes in the placebo group (20 pcs)<br><br>29 episodes in the OMA group treated with sulfisoxazole (21 pcs)<br><br>19 episodes of OMA in 22 pcs tube group tympanostomy (22 pcs)<br><br>Of the 3/20 placebo pz, there were no further episodes of AOM, compared to 12/22 in the group treated with TT (p=0.01). 5/21 of the prophylactic antibiotic patients had no further episodes of OMA (not statistically significant difference compared to the TT treated group )<br>Attack rate (infections/chi | Not declared |

| | | | | | | | | | |
|---|---|---|---|---|---|---|---|---|---|
| | | | | | | | | ld): placebo group 2.0, TT group 0.86 (p=0.06), prophylaxis antibiotic group 1.4.<br>12/20 (60%) placebo group failed treatment vs 5/22 (23%) tympanostomy tube treated group (p=0.02) vs 8/21 (38%) sulfisoxazole prophylaxis group failed treatment not significantly compared to others<br>In the TT group 9/22 patients were enrolled with exudative otitis media (EOM), both the attack rate and the number of new episodes of EOM were statistically better (p<0.05) in those who had OME at the beginning<br>19 patients (placebo group and antibiotic prophylaxis) failed treatment so they were treated with TT. Only 13 pieces have FU at three months: 11 had no more episodes of AOM.<br>4 of the 5 failed TT treatment units received chemotherapy, 3 without further problems, 1 with persistent otorrhea. | |
| Koivunen et al., 2004<br><br>Finlandia | Randomized, double-blind, controlled study | 180 children with RAOM (10 months - 2 years)<br>Hospital Finland<br>60 pcs adenoidectomy<br>60 pcs placebo<br>60 pcs chemotherapy | Children undergoing adenoidectomy or chemoprophylaxis (sulfisoxazole 50 mg/kg once daily for six months) to placebo | Failure of the intervention (two episodes in two months or three in six months or persistent effusion) in the first six months of follow-up | Average number of episodes of acute otitis media, medical visits, prescriptions for antibiotics, days of symptoms (rhinitis, earache, fever) and adverse events recorded in the diary | Identify the most effective preventive strategy to reduce RAOM incidents | 2 years | Chemotherapy vs placebo<br><br>Six-month failure of intervention FU 17 pcs in chemoprophylaxis (difference in failure rate 18%, 95% CI from -2 to 38)<br><br>Failure at 24 months 27 pcs ( differences in failure rate 15%; 95% CI from -4 to 35) | Not declared |
| Liston et al., 1984<br><br>USA | Non-randomized controlled trial | Children with three or more episodes of otitis media that occurred at least once every two months in the 13 weeks prior to the onset of prophylaxis. | Prophylaxis with sulphisoxazole, 75 mg per kg per day in two doses after 5 days from the acute episode vs exclusive treatment of | Efficacy of daily prophylaxis with sulphisoxazole, 75 mg/kg per day in two doses for 312 weeks (72 months), compared to | / | Difference in incidence of recurrent AOM between the 2 groups over 312 weeks (72 months) of observation. | Visit in case of suspected AOM for rhinitis, irritability, fever or otalgia.<br><br>Monthly interview on | The 26 patients in the study received sulfisoxazole for 312 weeks (72 months).<br>3 (11.5%) patients, who had two episodes of otitis media | Not declared |

| | | | | | | | | | |
|---|---|---|---|---|---|---|---|---|---|
| | | 26 patients (+ 1 excluded for allergic reaction after the prophylaxis started) recruited consecutively during January and February 1979.<br><br>Age < 5 years.<br><br>Mean age: 1.4 years, control population 1.6 years.<br>(control patients were selected of the same age and who had not yet received prophylaxis in the same period and before starting prophylaxis for the episodes of otitis media that occurred during the same weeks in which the patients in the intervention group were receiving chemoprophylaxis). | only the acute episode.<br><br>Both study and control patients started prophylaxis immediately after successfully finishing treatment for the acute episode. | the absence of prophylaxis in the prevention of acute otitis media (AOM) Recurrent in children who had three or more episodes of otitis media and at least one every two months. | | | any apparent side effects.<br><br>Diagnosis of AOM with pneumatic otoscopy.<br><br>Stop prophylaxis and tympanostomy in case of two episodes of otitis media. | during chemotherapy, underwent tympanostomy before completing the entire 3-month observation period and 2 (7.7%) completed ten weeks of follow-up (then moved away from the studio).<br><br>During this period, 11 patients had 16 episodes of otitis media, with a rate of 0.22 episodes per patient month, while the 26 untreated control patients had 63 episodes during the same period, obtaining a rate of 0.88 episodes per patient per month.<br><br>This 75% reduction in the incidence of otitis media is statistically significant (P <.005) (compared to different populations: 11 vs 26, not including 3 pcs that had to undergo tympanostomy).<br><br>The 21 patients who completed the whole study had nine episodes in 62 months (0.15 episodes per patient per month) vs controls, who had 51 episodes in the same period (0.82 episodes per patient per month), which represents an 82% reduction. | |
| Persico et al., 1985<br><br>Israel | Randomized controlled (random assignment, blind for examiners but not clear how blindness is maintained for examiners) | 111 patients with RAOM (defined as one or more episodes of acute otitis media, with or without purulent discharge, occurring at least once a month for 3 months)<br><br>2 groups by age<br>Group P 60, Group E 48 (3 | (1) group P (prophylaxis): treated with phenoxymethyl potassium penicillin V (penicillin V) 25 mg/kg/day for a minimum period of 3 months;<br><br>(2) Group E (episodic treatment): all received a short cycle (7-10 days) of ampicillin, 50 mg/kg/day in | Prophylaxis efficacy with potassium phenoxymethyl penicillin V (penicillin V) 25 mg/kg/day for a minimum of 3 months, compared to episodic treatment with a short cycle (7-10 days) of ampicillin, 50 mg/kg/day in the case of acute otitis media. | | Difference in incidence of recurrent AOM between the 2 groups in antibiotic prophylaxis and episodic treatment | For 6 (min)-15 (max) months.<br><br>Quarterly at the centre by two of the authors "blind" compared to the management group and in case of infection by the attending physician. | In 26 (42.7%) of the patients in the prophylaxis group (P) there were no new episodes of OMA.<br><br>In another 19.7% there was a partial improvement (reduction of the RAOM frequency to once every 3 months). | Not declared |

| | | | | | | | | | |
|---|---|---|---|---|---|---|---|---|---|
| | | out of 111 are missing):<br><br>-3-6 months 16.5% group P and 29.6% group E;<br><br>- 7-12 months (P) 47.2% and (E) 45%;<br><br>- 12-24 months (P) 34.0% versus (E) 36.5%;<br><br>(3) a control group of 26 patients, aged 3 to 8 years, who underwent the insertion of ventilation tubes (VT) for persistent secretory otitis media. | case of acute otitis media, as diagnosed by the family doctor. | | | | | In group E (episodic treatment) 14.7% of patients, while 25% showed partial improvement.<br><br>36.6% in P group and 60.4% in E group showed no reduction of RAOM frequency. | |
| Prellner et al., 1994<br><br>Sweden | Randomized, controlled, multicenter, double-blind study 76 children | 76 children<br><br>Inclusion criteria: 3 or more episodes of OMA in the 6 months preceding the study; age less than 18 months.<br><br>Exclusion criteria: severe underlying disease, penicillin allergy, children treated with tympanostomy tubes.<br><br>AOM was diagnosed by otomicroscopy and defined as an acute episode of earache in a child with red tympanum and outflow. | Administration of penicillin V (pcV) 25 mg/kg body weight b.i.d. vs placebo for 10 days in case of respiratory tract infection episode (RTI). | Effectiveness of administration of pcV during an episode of RTI in preventing recurrent attacks of AOM in children prone to otitis. | / | Difference in incidence of AOM between the 2 groups over the observation period | Once a month, from January to June in the study periods (1990/1991 and 1991/1992). | Prophylaxis with pcV in-progress RTI in children with a predisposition to otitis has reduced the incidence of recurrent episodes of AOM by 50% compared to placebo ($p<0.001$). | Not declared |
| Schuller_et al.1983<br><br>USA | RCT not blind | 72 patients<br>2-6 years<br>Inclusion criteria:<br><br>bronchial asthma developed at least 6 months before<br><br>4 or more episodes of AOM documented with physical examination (fever greater than 38°C and immobile swelling of the tympanic membrane) in the previous 12 months, confirmed by pneumatic otoscopy. | Group 1: treated with antibiotics after each episode (controls)<br><br>Group 2: antihistamine (chlorfenirami ne maleate) to be taken at the first nasal symptoms<br><br>Group 3: prophylaxis with sulphyoxazole 500 mg twice daily for 2 years<br>3A: sulphyoxazole only<br><br>3B*: sulphisoxazole + anti-VCP vaccination<br><br>Group 4: pneumococcal vaccine | Effectiveness of otitis media prophylaxis in asthmatic patients in different groups | / | Reduction % in the incidence of OMA between different groups over 2 years of observation difference in the occurrence of side effects | 2 years of follow-up<br><br>visit every 3 months (early in case of pathology)<br>in the cases treated with sulphoxazole:<br><br>blood test at the first month and then every 6 months for 2 years | Group 2 (antihistamine ): slight reduction (compared to group 1) not significant in the incidence of AOM (12% less at 1 year, 11% at 2 years, $P > 0.05$).<br><br>Group 3A (sulphisoxazol e): significant reduction of AOM episodes (70% reduction in the 1st year, 79% in the 2nd year, $p < 0.001$) even more pronounced in group 3B who also carried out anti-pneumococcal vaccination (70% in the 1st year, 93% in the second year) | Not declared |

| | | | | | | | | | |
|---|---|---|---|---|---|---|---|---|---|
| | | | 4A: vaccine only<br><br>4B*: PCV vaccination + prophylaxis with sulphisoxazole<br><br>Group 5: prophylaxis with sulphisoxazole + pneumococcal vaccine<br><br>*Subgroup B: after 12 months of treatment, 12 patients receiving sulphisoxazole alone or pneumococcal vaccination alone were given additional therapy by placing them in a double-treated group | | | | | Group 4A (pneumococcal vaccination): 41% reduction in 1° year, 36% in 2° year ($p = 0.011$). In group 4B: 41% in the 1st year, 89% in the 2nd year<br><br>Group 5 (sulphisoxazole + anti pneumococcal ab initio): 90% reduction in the 1st year, 91% in the 2nd year ($p < 0.001$)<br><br>In subjects treated with sulphisoxazole: no adverse effects, blood count in the norm.<br>In patients treated with anti pneumococcal: local pain (35%), local erythema (11%), fever <39°C (5%) | |
| Schwartz et al., 1982<br><br>USA | Randomized, cross-over, double blind, placebo controlled study | 43 children enrolled. Of these, 33 have completed the study.<br><br>Age 4 to 72 months (average 31 months).<br><br>Inclusion criteria: all children < or = 72 months with at least 3 episodes of OMA in the previous 12 months.<br>OMA defined by 3 criteria:<br>(1) Opacified tympanum extroflection (bulging);<br>(2) reduced mobility of the eardrum;<br>(3) Fire yellow or red color of the tympanic membrane. | A single daily dose of sulfamethoxazole (SMZ) 25 mg/kg vs placebo.<br><br>After the first two months, each child was given alternative treatment. | Effectiveness of prophylaxis with SMZ in the prevention of recurrent AOM or effusive otitis media in children with a predisposition to otitis. | / | Difference in incidence of AOM between the 2 groups over the observation period | The otoscopy and tympanometry were performed on each child at the time of study enrollment, 2 and 4 weeks later, and then monthly for the next three months. | 9 of the 33 (27%) children treated with SMZ experienced 10 episodes of AOM or effusive otitis media (EOM ) vs 19 out of 33 (57.5%) children in the placebo group who experienced 27 episodes ($p<0.02$).<br>.<br>During the first 2 months of study, 4 episodes of acute suppurative otitis media or OME were observed in 17 children who received SMZ prophylactic vs 12 episodes in 16 children in the placebo group ($p<0.01$).<br>After cross-over (2 months later), 5 episodes of AOM were reported in 17 children who received the placebo.<br><br>No significant difference was observed between the 2 treatment groups during the second phase of the study.<br>However, there was a | Not declared |

| | | | | | | | | | |
|---|---|---|---|---|---|---|---|---|---|
| | | | | | | | | significant decrease in AOM episodes (from 12 in the 16 children who received placebo during phase one to 5 episodes after cross-over to SMZ (p<0.02)).<br><br>The number of AOM episodes in children starting the placebo study (12 episodes in 16 patients) differs significantly from the number of AOM episodes observed in the group starting the SMZ prophylaxis study (7 episodes in 17 patients) (p<0.05).<br><br>Regardless of treatment type, 11 (33%) of the 33 children in the study had no AOM episodes during the 4-month study period.<br><br>No child has experienced severe side effects, only rash morbilliforme | |
| Sih et al., 1993<br><br>Brasil | Double-blind randomized, placebo-controlled study | 60 patients enrolled from March 1989 to March 1990 (12 months)<br><br>Average age 34 months<br><br>Randomized:<br>20 pcs TMP-SMZ (12 mg/kg/day)<br>20 pcs AMX (20 mg/kg/day)<br>20 pcs Placebo<br><br>Antibiotic administered by the parent once a day, at bedtime, for three months.<br><br>Inclusion criterion: at least three episodes of AOM in the previous 12 months.<br>All patients at the time of inclusion in the study had an AOM treated with amoxicillin for 10 days and reassessed healing with pneumatocopy and | Document the efficacy of antimicrobial prophylaxis x three months (AMX and TMP-SMZ) vs no therapy in reducing recurrent acute otitis media.<br><br>Episodes of AOM during this period were treated with cefaclor (50 mg/kg/day, 3 times daily, for 10 days); after which the patient was returned to the original prophylaxis group. | To evaluate the efficacy of amoxicillin (AMX) and trimethoprim sulfamethoxazole (TMP-SMZ) for prophylaxis of recurrent AOM in a population of Brazilian children | | Difference in incidence of recurrent AOM in children who received placebo compared to those who received antibiotics | Monthly with pneumootoscopy and tympanometric impedance<br><br>Otoscopic reassessment if AOM | Of the 20 patients receiving TMP-SMZ therapy, 4 (20%) had relapses during the 3-month study period (3 children had a single relapse and one had two recurrent episodes)<br><br>Of the 20 patients taking AMX, 3 (15%) had relapses of AOM (each with a single episode).<br><br>10 (50%) of the 20 patients who received PLACEBO had relapses (7 children had 1 episode, 2 children had 2 episodes and 1 child had 3 episodes)<br><br>The rate of recurrent OMA was significantly higher in children who received placebo than those who | |

| | | | | | | | | | |
|---|---|---|---|---|---|---|---|---|---|
| | | impedance/tympanometry | | | | | | received one of the two antibiotics (50% versus 18%, P < 0.0005).<br><br>No statistically significant difference in efficacy between the two antibiotics.<br><br>More children treated with placebo had a recurrent disease than those treated with antibiotics, and among children with recurrent AOM, episodes occurred earlier in the placebo group.<br><br>Children in the treatment groups were significantly less likely to have recurrent AOM during the first month of the study compared with the second 2 months (Odds Ratio = undefined, P < 0.002).<br><br>No adverse effects of antibiotic therapy occurred during the study. | |

| | |
|---|---|
| ***Question 10: In children allergic to penicillins, which antibiotics can be used in AOM/RAOM therapy?*** | **P** In children allergic to penicillins with AOM/RAOM<br>The therapy with:<br>**I1** cephalosporins of 2nd generation<br>**I2** 3rd generation cephalosporins<br>Quinolones<br>**C** compared to macrolides<br>**O1** is as/more effective in achieving healing?<br>**O2** is as/more effective in reducing the risk of short-term recurrence?<br>**O3** is as/more effective in reducing the risk of RAOM?<br>**O4** is as/more effective in reducing the risk of suppurative complications? |

**Table S4.18. Question 10. NICE GL 2018 Recommendations and Grading**

| | |
|---|---|
| **Question source.**<br>NICE guideline [NG91] 2018 Evidence Review Appendix E – H<br><br>***NICE Question***<br>***Antibiotic choice*** | |
| **Source of Recommendations Appendix E - H**<br>NICE guideline [NG91] 2018<br><br>Recommendation 1.2.1. Recommendation 1.2.1 Follow the instructions in Table 1.<br><br>First choice alternative for allergy or intolerance to penicillin (not in pregnancy)<br><br>Clarithromycin: The<br>From 1 month to 11 years: | **Grading**<br><br>1.2.1 Quality of evidence low for the efficacy of macrolides vs penicillin. Very low quality of evidence for macrolide choice (expert opinion on current practice)<br>Strong recommendation for intervention |

| | |
|---|---|
| under 8 kg, 7.5 mg/kg twice daily for 5-7 days<br>8 kg to 11 kg, 62.5 mg twice daily for 5-7 days<br>12 kg to 19 kg, 125 mg twice daily for 5-7 days<br>From 20 kg to 29 kg, 187.5 mg twice daily for 5-7 days<br>From 30 kg to 40 kg, 250 mg twice daily for 5-7 days<br>From 12 to 17 years, 250 mg to 500 mg twice daily for 5-7 days<br><br>Second alternative choice for allergy or intolerance to penicillin<br>Consult local microbiologist<br><br>Based on evidence, their experience and resistance data, the committee agreed to recommend clarithromycin as an alternative first choice antibiotic for use in penicillin allergy or amoxicillin intolerance. Recommended doses (based on weight and age) are the usual doses for children and were similar to those used in the studies in the trial review. The committee discussed the existence of evidence for another macrolide, azithromycin. However, they decided not to recommend this because clarithromycin or erythromycin is a standard practice and azithromycin should be reserved for more severe infections. | |

| | |
|---|---|
| ***Question 11: Are parenteral antibiotics, specifically intramuscular, more effective than amoxicillin/amoxicillin-ac clavulanic for the WMO?*** | **P** In children with AOM<br>**I** The therapy with antibiotics by injection (ceftriaxone or other i.m.)<br>**C** compared to amoxicillin/amoxicillin-ac clavulanic therapy for the<br>**O1** is significantly more effective and faster in achieving healing?<br>**O2** is significantly more effective in reducing the risk of short-term recurrence?<br>**O3** is significantly more effective in reducing the risk of OMAR?<br>**O4** is significantly more effective in reducing the risk of suppurative complications? |

**Table S4.19. Question 11. NICE GL 2018 Recommendations and Grading**

| | |
|---|---|
| **Question source.**<br>NICE guideline [NG91] 2018 Evidence Review Appendix E – H<br><br>***NICE Question***<br>***Antibiotic choice*** | |
| **Source of Recommendations**<br>NICE guideline [NG91] 2018<br><br>Recommendation 1.2.1 Follow the instructions in Table 1.<br><br>First choice oral antibiotic: Amoxicillin.<br><br>Second choice oral antibiotic: Amoxicillin-clavulanic<br>(worsening of symptoms after at least 2 or 3 days of first choice therapy)<br><br>There were no substantial differences in treatment success between classes of antibiotics, including penicillins, cephalosporins and macrolides for the treatment of uncomplicated acute otitis media in children. There was no difference in treatment success between ampicillin or amoxicillin versus ceftriaxone; co-amoxiclav versus ceftriaxone; co-amoxiclav versus azithromycin; or cefaclor versus azithromycin (low to moderate quality evidence). This was based on a systematic review and meta-analysis of RTCs (Shekelle et al. 2010).<br><br>Co-amoxiclav has been associated with a significantly higher number of adverse events than cephalosporin (very low to moderate quality evidence) or azithromycin (moderate quality evidence; Shekelle et al. 2010).<br><br>Based on evidence of no substantial differences in clinical efficacy between classes of antibiotics, the committee agreed that antibiotic choice should be largely guided by minimising the risk of resistance.<br><br>The committee discussed that if an antibiotic is needed to treat an infection which is not life threatening, a narrow-spectrum antibiotic should generally be the first choice. The indiscriminate use of broad-spectrum antibiotics creates a selective advantage for bacteria resistant even to these broad-spectrum "last line" agents and also kills normal commensal flora leaving people susceptible to antibiotic-resistant harmful bacteria such as Clostridium difficile. For infections that are not life threatening, broad-spectrum antibiotics should be reserved for second-choice treatment when narrow-spectrum antibiotics are ineffective. | **Grading**<br><br>1.2.1. Quality of evidence moderate (penicillins vs cephalosporins), low (penicillins vs. macrolides). Strong recommendation for intervention |

| | |
|---|---|
| ***Question 12: Topical antibiotic therapy may be useful in the treatment of perforated acute otitis?*** | **P** In children with AOM and otorrhea<br>**I** The combination of topical and oral therapy<br>**C** compared to oral therapy<br>**O1** is more effective in achieving healing?<br>**O2** is more effective in reducing the risk of short-term recurrence?<br>**O3** is more effective in reducing the risk of RAOM?<br>**O4** is more effective in reducing the risk of suppurative complications? |

**Table S4.20. Question 12. SIP GL 2019 Recommendations and Grading**

| | |
|---|---|
| **Question source.**<br>SIP GL 2019 - Treatment<br><br>***Question from the 2019 SIP GLs***<br>***Question 8. What is the role of topical antibiotic or steroid ear therapy in AOM?*** | |
| **Source of Recommendations**<br>SIP GL 2019<br><br>Recommendation No 18. Topical ear therapy with antibiotics, in combination or not to the steroid, is not recommended except in children with otorrhea by ventilation tubes.<br><br>Many antibiotics are used for ototopic use: chlortetracycline (burdened by the presence of many resistances due to its use in animal husbandry), ciprofloxacin (whose administration for ototopic use was approved by the FDA in 2005), clioquinol and "oldest" chloramphenicol, neomycin (very ototoxic), tobramycin and sulfadide.<br>The effectiveness of endoauricular antibiotic therapy has been evaluated through experimental research and clinical studies, Often on heterogeneous samples that mainly included subjects suffering from external otitis together with subjects with otitis media with or without perforation of the tympanic membrane, treated with combinations of different antibiotics.<br><br>Other good methodological studies have evaluated the efficacy of topical antibiotic therapy in a specific clinical indication (otorrhea in OMA with ventilation tubes): these results are certainly not sufficient to suggest changes to antibiotic therapy in OMA, Although complicated by spontaneous perforation. | **Grading**<br><br>Strong negative recommendation |

| | |
|---|---|
| ***Question 13: Is an antibiotic other than amoxicillin indicated for perforated acute otitis?*** | **P** In children with perforated AOM<br>**I1** Amoxicillin-clavulanic acid<br>**I2** cephalosporin<br>**I3** Quinolone<br>**C** compared to amoxicillin<br>**O1** is more effective in achieving healing?<br>**O2** is more effective in reducing the risk of short-term recurrence?<br>**O3** is more effective in reducing the risk of OMAR?<br>**O4** is more effective in reducing the risk of suppurative complications? |

**Table S4.21. Question 13. NICE GL 2018 Recommendations and Grading**

| | |
|---|---|
| **Question source.**<br>NICE guideline [NG91] 2018 Evidence Review Appendix E – H<br><br>***NICE Question***<br>***Children and young people who may benefit more from antibiotics (those of any age with otorrhea or those under 2 years with bilateral AOM) Antibiotic choice*** | |
| **Source of Recommendations**<br>NICE guideline [NG91] 2018<br><br>Recommendation 1.2.1 Follow the instructions in Table 1.<br><br>First choice oral antibiotic: Amoxicillin.<br><br>Second choice oral antibiotic: Amoxicillin-clavulanic<br>(worsening of symptoms after at least 2 or 3 days of first choice therapy<br><br>The most common complications of acute otitis media are relapse, hearing loss (usually temporary), and perforation of the eardrum. However, antibiotics make little difference on the risk of these complications (see antibiotic efficacy). [2018] | **Grading**<br><br>1.2.1. Quality of evidence moderate (penicillins vs cephalosporins), low (penicillins vs. macrolides). Strong recommendation for intervention |

**Table S4.22. Question 13. SIP GL 2019 Recommendations and Grading**

| Question source.<br>SIP GL 2019 - Treatment<br><br>***Question from the 2019 SIP GLs***<br>***Question 3. What molecules are recommended for antibiotic therapy??*** | |
|---|---|
| **Source of Recommendations**<br>SIP GL 2019<br><br>Recommendation 8. In case of AOM in children who have taken antibiotic therapy within the previous 30 days, in those with severe symptoms, and/or purulent conjunctivitis, or with history of recurrent AOM not responsive to amoxicillin, Spontaneous perforation othorrhea or in those with a high risk of resistant pathogens (frequency of infant community, non-vaccination against pneumococcal disease, from geographical areas with high prevalence of isolation of resistant bacteria) Amoxicillin- clavulanic acid is recommended at a dose of 80 - 90 mg /kg/day (calculated on amoxicillin)<br><br>The addition of clavulanic acid to amoxicillin effectively neutralizes even microorganisms such as Haemophilus influenzae and Moraxella catarrhalis producers of β-lactamase while maintaining an excellent antibacterial activity towards penicillin-strainsresistant of S. pneumoniae. (Pichichero 2013, Mittal 2018).<br><br>The association is considered by many authors preferable in children who have taken antibiotic therapy within the previous 30 days, in those with severe symptoms, and/or purulent conjunctivitis, or with history of recurrent AOM not responsive to amoxicillin, or in those with a high risk of resistant pathogens (frequency of infant community, lack of pneumococcal vaccination, coming from geographic areas with high prevalence of isolation of resistant bacteria (Lieberthal 2013, Rybak 2018, Marchisio 2017, Ovnat Tamir 2017, Harrison 2018).<br><br>In children with AOM with otorrhea from spontaneous perforation, given the relevance of beta-lactamase producing pathogens, the association amoxicillin-clavulanic acid has been suggested by some authors (Marchisio 2017). | **Grading**<br><br>8. Strong recommendation for intervention |
