# Supplementary material for: Antibiotic treatment of acute and recurrent otitis media in children: an Italian intersociety Consensus
Source: Ital J Pediatr. 2025 Feb 20;51:50. doi: 10.1186/s13052-025-01894-z (PMC11844117; doi:10.1186/s13052-025-01894-z)

S5. META-ANALYSIS

RAOM ANTIBIOTIC PROPHYLAXIS

Analysis 5.1 Amoxicillin-clavulanic acid compared with placebo

	Amoxi/Amoxi-Clav.		Control			Risk Ratio
**Study or Subgroup**	**Events**	**Total**	**Events**	**Total**	**Weight**	**M-H, Random, 95% CI**
Prellner 1994	17	35	39	41	65.8%	0.51 [0.36, 0.72]
Sih 1993	3	20	14	20	34.2%	0.21 [0.07, 0.63]
**Total (95% CI)**		**55**		**61**	**100.0%**	**0.38 [0.16, 0.90]**
Total events	20		53			

Heterogeneity: Tau² = 0.26; Chi² = 2.57, df = 1 (P = 0.11); I² = 61%
Test for overall effect: Z = 2.20 (P = 0.03)

Risk Ratio
M-H, Random, 95% CI

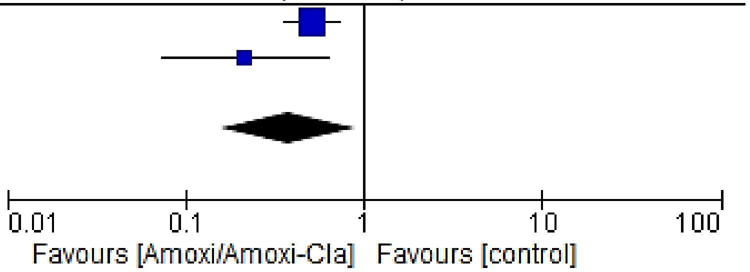

Analysis 5.2 Penicillin V compared with placebo

	PenicillinaV		Control			Risk Ratio
**Study or Subgroup**	**Events**	**Total**	**Events**	**Total**	**Weight**	**M-H, Fixed, 95% CI**
Persico 1985	22	60	29	48	100.0%	0.61 [0.41, 0.91]
**Total (95% CI)**		**60**		**48**	**100.0%**	**0.61 [0.41, 0.91]**
Total events	22		29			

Heterogeneity: Not applicable
Test for overall effect: Z = 2.42 (P = 0.02)

Risk Ratio
M-H, Fixed, 95% CI

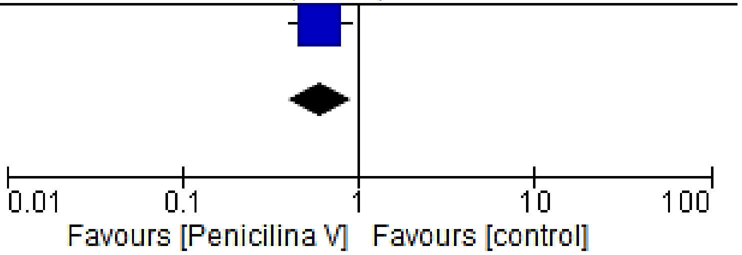

Analysis 5.3 TMP-SMZ compared with placebo

	TMP-SMZ		Control			Risk Ratio
Study or Subgroup	Events	Total	Events	Total	Weight	M-H, Fixed, 95% CI
Gaskins 1992	0	10	8	11	12.2%	0.06 [0.00, 0.99]
Gonzalez 1986	16	21	17	20	26.2%	0.90 [0.66, 1.21]
Schwartz 1982	10	33	27	33	40.6%	0.37 [0.22, 0.64]
Sih 1993	5	20	14	20	21.0%	0.36 [0.16, 0.80]
**Total (95% CI)**		**84**		**84**	**100.0%**	**0.47 [0.35, 0.63]**
Total events	31		66			

Heterogeneity: Chi² = 21.01, df = 3 (P = 0.0001); I² = 86%
Test for overall effect: Z = 5.03 (P < 0.00001)

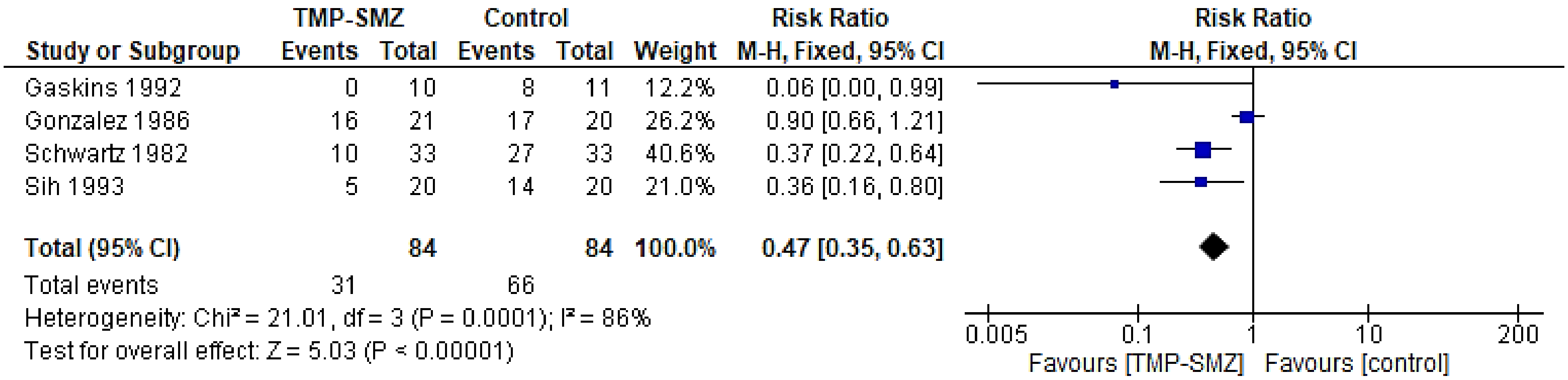

Analysis 5.4 Sulfisoxazole compared with placebo

	Sulfisoxazole		Control			Risk Ratio
Study or Subgroup	Events	Total	Events	Total	Weight	M-H, Fixed, 95% CI
Liston 1984	3	26	11	26	100.0%	0.27 [0.09, 0.87]
**Total (95% CI)**		**26**		**26**	**100.0%**	**0.27 [0.09, 0.87]**
Total events	3		11			

Heterogeneity: Not applicable
Test for overall effect: Z = 2.20 (P = 0.03)

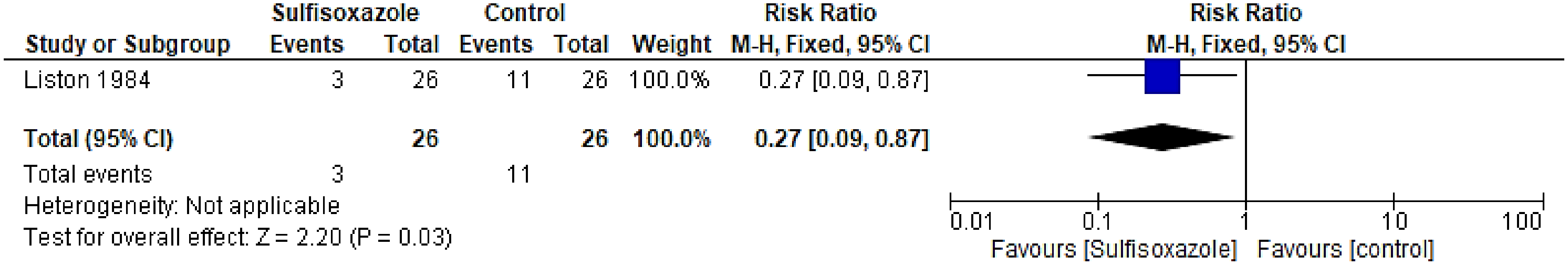

Analysis 5.5 Sulfisoxazole/Sulfafurazole compared with placebo

Study or Subgroup	Sulfisoxazolo/Sulfafurazole Mean	SD	Total	Control Mean	SD	Total	Weight	Mean Difference IV, Random, 95% CI
Gonzalez 1986	2	1	20	1.4	1	21	33.1%	0.60 [-0.01, 1.21]
Koivunen 2004	1	0.8	60	1.3	0.9	60	33.4%	-0.30 [-0.60, 0.00]
Schuller 1983	1.3	0.14	12	5.1	0.23	12	33.5%	-3.80 [-3.95, -3.65]
**Total (95% CI)**			**92**			**93**	**100.0%**	**-1.18 [-4.07, 1.72]**

Heterogeneity: Tau² = 6.50; Chi² = 539.14, df = 2 (P < 0.00001); I² = 100%
Test for overall effect: Z = 0.80 (P = 0.43)

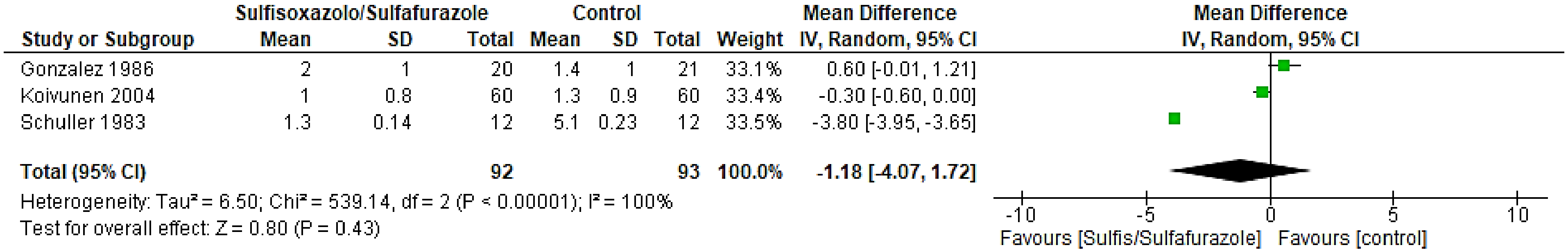

Supplement: Supplementary file 5 — Additional file 5. S5_RAOM META-ANALYSIS.pdf (RAOM antibiotic prophylaxis meta-analysis). [file 13052_2025_1894_MOESM5_ESM.pdf]
