# Supplementary material for: Antibiotic treatment of acute and recurrent otitis media in children: an Italian intersociety Consensus
Source: Ital J Pediatr. 2025 Feb 20;51:50. doi: 10.1186/s13052-025-01894-z (PMC11844117; doi:10.1186/s13052-025-01894-z)
Supplement: Supplementary file 6 — Additional file 6. S6_AOM-RAOM GRADE.pdf (RAOM GRADE evidence profile). [file 13052_2025_1894_MOESM6_ESM.pdf]

S6. RECURRENT ACUTE OTITIS MEDIA

GRADE EVIDENCE PROFILE

In children with RAOM, is the use of antibiotic prophylaxis effective in reducing the recurrence of episodes?

Patient or population: child with RAOM

Setting: Outpatient

Intervention: antibiotic prophylaxis with penicillins

Comparison: Compared to no prophylaxis

Certainty assessment							№ of patients		Effect		Certainty	Importance
№ of studies	Study design	Risk of bias	Inconsistency	Indirectness	Imprecision	Other considerations	[Profilassi antibiotica - penicilline]	[nessuna profilassi]	Relative (95% CI)	Absolute (95% CI)		

Table S6.1. Reduction in the number of AOMs (follow-up: range 3 months to 12 months; assessed with: AOM incidence in the follow-up period)

2[1,2]	randomised trials	serious[a,b]	not serious	not serious	not serious	publication bias strongly suspected[c,d]	20/55 (36.4%)	53/61 (86.9%)	**RR 0.38** (0.16 to 0.90)	**539 fewer per 1.000** (from 730 fewer to 87 fewer)	⨁⨁◯◯ Low	CRITIC

Table S6.2. Reduction in the number of AOM - prophylaxis with penicillin V in doses of 25 mg/kg/day (follow-up: range 3 months to 15 months; assessed with: number of children without any improvement, either total or partial (max 1 AOM in 3 months))

In children with RAOM, is the use of antibiotic prophylaxis effective in reducing the recurrence of episodes?

Patient or population: child with RAOM

Setting: Outpatient

Intervention: antibiotic prophylaxis with penicillins

Comparison: Compared to no prophylaxis

Certainty assessment							№ of patients		Effect		Certainty	Importance
№ of studies	Study design	Risk of bias	Inconsistency	Indirectness	Imprecision	Other considerations	[Profilassi antibiotica - penicilline]	[nessuna profilassi]	Relative (95% CI)	Absolute (95% CI)		
1[3]	randomised trials	serious[a,b]	not serious	not serious	not serious	all plausible residual confounding would reduce the demonstrated effect[e]	22/60 (36.7%)	29/48 (60.4%)	**RR 0.61** (0.41 to 0.91)	**236 fewer per 1.000** (from 356 fewer to 54 fewer)	⊕⊕⊕⊕ High	CRITIC

CI: confidence interval; **RR:** risk ratio

Explanations

at. Randomization: Unclear risk of bias

b. Allocation concealment: high risk of bias

c. clinically insignificant results: average <1 AOM/pc in the follow-up period

d. Low sample size

e. Correlation between therapeutic success and breast milk intake in both groups

References

1.Prellner K, Foglé-Hansson M,Jørgensen F,Kalm O,Kamme C. Prevention of recurrent acute otitis media in otitis-prone children by intermittent prophylaxis with penicillin. Acta Otolaryngol. 1994 Mar, 1983, 114(2):182-7.,Schuller_et,al... .

2.Sih T, Moura R,Caldas S,Schwartz B. Prophylaxis for recurrent acute otitis media: a Brazilian study. Int J Pediatr Otorhinolaryngol. 1993 Jan, 25(1-3):19-24.

3.Persico M., Podoshin L.,Fradis M.,et al. Recurrent acute otitis media - Prophylactic penicillin treatment: A prospective study. Part I. International Journal of Pediatric Otorhinolaryngology 1985 10:1 (37-46)..

In children with RAOM, is the use of antibiotic prophylaxis effective in reducing the recurrence of episodes?

Patient or population: child with RAOM

Setting: Outpatient

Intervention: antibiotic prophylaxis with sulfonamides or macrolides

Comparison: Compared to no prophylaxis

Certainty assessment							№ of patients		Effect		Certainty	Importance
№ of studies	Study design	Risk of bias	Inconsistency	Indirectness	Imprecision	Other considerations	[Profilassi antibiotica - Sulfamidici]	[nessuna profilassi]	Relative (95% CI)	Absolute (95% CI)		
**Table S6.3. Reduction in the number of AOMs - TMP-SMZ prophylaxis (follow-up: range 3 months to 6 months; assessed with: AOM incidence in the follow-up period)**												
4[1,2,3,4]	randomised trials	serious[a,b]	not serious	not serious	not serious	publication bias strongly suspected[c]	31/84 (36.9%)	66/84 (78.6%)	**RR 0.47** (0.35 to 0.63)	**416 fewer per 1.000** (from 511 fewer to 291 fewer)	⊕⊕◯◯ Low	CRITIC

Table S6.4. Reduction number of AOM - Sulfonamides (follow-up: range 3 months to 24 months; assessed with: No. of AOM/patient in the follow-up period - Average (DS))

In children with RAOM, is the use of antibiotic prophylaxis effective in reducing the recurrence of episodes?

Patient or population: child with RAOM

Setting: Outpatient

Intervention: antibiotic prophylaxis with sulfonamides or macrolides

Comparison: Compared to no prophylaxis

Certainty assessment							№ of patients		Effect		Certainty	Importance
№ of studies	Study design	Risk of bias	Inconsistency	Indirectness	Imprecision	Other considerations	[Profilassi antibiotica - Sulfamidici]	[nessuna profilassi]	Relative (95% CI)	Absolute (95% CI)		
3[2,5,6]	randomised trials	serious[d]	not serious	not serious	not serious	publication bias strongly suspected[d]	92	93	-	MD **1.18 fewer** (4.07 fewer to 1.72 more)	⊕⊕◯◯ Low	CRITIC

Table S6.5. Reduction in AOM number - Sulfisoxazole - NRCT (follow-up: mean 72 months; assessed with: incidence of AOM/year/group)

1[7]	non-randomised studies[e]	serious[e,f]	not serious	not serious	not serious	publication bias strongly suspected all plausible residual confounding would reduce the	3/26 (11.5%)	11/26 (42.3%)	**RR 0.27** (0.09 to 0.87)	**309 fewer per 1.000** (from 385 fewer to 55 fewer)	⊕⊕⊕◯ Moderate	CRITIC

In children with RAOM, is the use of antibiotic prophylaxis effective in reducing the recurrence of episodes?

Patient or population: child with RAOM

Setting: Outpatient

Intervention: antibiotic prophylaxis with sulfonamides or macrolides

Comparison: Compared to no prophylaxis

Certainty assessment							№ of patients		Effect		Certainty	Importance
№ of studies	Study design	Risk of bias	Inconsistency	Indirectness	Imprecision	Other considerations	[Profilassi antibiotica - Sulfamidici]	[nessuna profilassi]	Relative (95% CI)	Absolute (95% CI)		
						demonstrated effect[g]		42.3%		**309 fewer per 1.000** (from 385 fewer to 55 fewer)		

CI: confidence interval; **MD:** mean difference; **RR:** risk ratio

Explanations

a. Randomizzazione: UNCLEAR rischio di bias in 3 su 4 RCT

b. Allocation concealment: alto rischio di bias in 3 su 4 RCT

c. risultati clinicamente non rilevanti: mediamente meno di 1 AOM/pz in 3-6 mesi anche nel gruppo controllo

d. risultati discordanti rispetto agli studi che valutano l'incidenza. 2 su 3 RCT di migliore qualità metodologica non riportano differenze statisticamente significative

e. Studio di intervento controllato non randomizzato

f. mancanza di cecità

g. risultati per paziente clinicamente non rilevanti sia nel gruppo intervento che nel gruppo controllo: rispettivamente 0,1/pz/anno e 0.4/pz/anno

References

1.Gaskins JD, Holt RJ,Kyong CU,Weart CW,Ward J. Chemoprophylaxis of recurrent otitis media using trimethoprim/sulfamethoxazole. Drug Intell Clin Pharm. 1982 May, 16(5):387-90. Gonzalez et al.., 1986. .

2.Gonzalez C., Arnold J.E.,Erhardt J.B.,et al. Prevention of recurrent acute otitis media chemoprophylaxis versus tympanostomy tubes Laryngoscope 1986 96:12 (1330-1334).. .

3.Schwartz RH, Puglise J,Rodriguez WJ. Sulphamethoxazole prophylaxis in the otitis-prone child. Arch Dis Child. 1982 Aug, 57(8):590-3, . .

4.Sih T, Moura R,Caldas S,Schwartz B. Prophylaxis for recurrent acute otitis media: a Brazilian study. Int J Pediatr Otorhinolaryngol. 1993 Jan, 25(1-3):19-24., . .

5.Koivunen P., Uhari M.,Luotonen J.,Kristo A.,Raski R.,Pokka T.,Alho O.-P. Adenoidectomy versus chemoprophylaxis and placebo for recurrent acute otitis media in children aged under 2 years: Randomised controlled trial. British Medical Journal 2004 328:7438 (487-490). .

6.1983, Schuller_DE.,Prophylaxis,of,otitis,media,in,asthmatic,children.,Pediatric,Infectious,Disease, 2(4):280-3., . .

7.Liston TE, Harbison R. Sulfisoxazole chemoprophylaxis and recurrent otitis media. West J Med. 1984 Jan, 140(1):47-9, . .
