# Supplementary material for: Antibiotic treatment of acute and recurrent otitis media in children: an Italian intersociety Consensus
Source: Ital J Pediatr. 2025 Feb 20;51:50. doi: 10.1186/s13052-025-01894-z (PMC11844117; doi:10.1186/s13052-025-01894-z)
Supplement: Supplementary file 7 — Additional file 7. S7_AOM-RAOM References of tables.pdf (reference of tables). [file 13052_2025_1894_MOESM7_ESM.pdf]

S7. AOM-RAOM References of tables

Included GLs

- NICE guideline [NG91] Otitis media (acute): antimicrobial prescribing. Disponibile al link https://www.nice.org.uk/guidance/ng91 (ultimo accesso 14.08.2022)
- Linea Guida italiana 2019 Gestione dell'otite media acuta in età pediatrica: diagnosi, terapia e prevenzione. Disponibile al link https://www.sipps.it/wp/wp-content/uploads/2019/05/OTITE-MEDIA-ACUTA.pdf (ultimo accesso 14.08.2022)

Excluded GLs

- Leach AJ, Morris PS, Coates HL, et al. Otitis media guidelines for Australian Aboriginal and Torres Strait Islander children: summary of recommendations. Med J Aust. 2021 Mar;214(5):228-233. doi:10.5694/mja2.50953. Epub 2021 Feb 28.

Excluded SR

AOM Questions

- Djabali EJ, Smolinski NR, Al-Bahou J, Pomputius A, Antonelli PJ, Winterstein AG; Antibiotic Treatment for Pediatric Acute Otitis Media and the Prevention of Serious Complications: A Meta-analysis. Pediatrics February 2022; 149 (1 Meeting Abstracts February 2022): 143.
- Dawit G, Mequanent S, Makonnen E. Efficacy and safety of azithromycin and amoxicillin/clavulanate for otitis media in children: a systematic review and meta-analysis of randomized controlled trials. Ann Clin Microbiol Antimicrob. 2021 Apr 24;20(1):28
- Holm NH, Rusan M, Ovesen T. Acute otitis media and antibiotics - a systematic review. Dan Med J. 2020 Oct 29;67(11):A04200272.
- Spurling GK, Del Mar CB, Dooley L, Foxlee R, Farley R. Delayed antibiotic prescriptions for respiratory infections. Cochrane Database Syst Rev. 2017 Sep 7;9(9):CD004417.

Question 9 - RAOM

- Cheong KH, Hussain SS. Management of recurrent acute otitis media in children: systematic review of the effect of different interventions on otitis media recurrence, recurrence frequency and total recurrence time. J Laryngol Otol. 2012 Sep;126(9):874-85. doi: 10.1017/S0022215112001338. Epub 2012 Jul 5. PMID: 22874133.

Included studies

AOM Questions

- Hoberman A, Paradise JL, Rockette HE, Kearney DH, Bhatnagar S, Shope TR, Martin JM, Kurs-Lasky M, Copelli SJ, Colborn DK, Block SL, Labella JJ, Lynch TG, Cohen NL, Haralam M, Pope MA, Nagg JP, Green MD, Shaikh N. Shortened Antimicrobial Treatment for Acute Otitis Media in Young Children. N Engl J Med. 2016 Dec 22;375(25):2446-2456.

- Shahbaznejad L, Talaei E, Hosseinzadeh F, Masoumi B, Rezai S, Rezai MS. Comparing Watchful Waiting Approach vs. Antibiotic Therapy in Children with Nonsevere Acute Otitis Media: A Randomized Clinical Trial. Int J Pediatr. 2021 May 27;2021:551546

Question 9 - RAOM

- Gaskins JD, Holt RJ, Kyong CU, Weart CW, Ward J. Chemoprophylaxis of recurrent otitis media using trimethoprim/sulfamethoxazole. Drug Intell Clin Pharm. 1982 May;16(5):387-90. Gonzalez et al.., 1986
- Gonzalez C., Arnold J.E., Erhardt J.B., et al. Prevention of recurrent acute otitis media chemoprophylaxis versus tympanostomy tubes Laryngoscope 1986 96:12 (1330-1334).
- Koivunen P., Uhari M., Luotonen J., Kristo A., Raski R., Pokka T., Alho O.-P. Adenoidectomy versus chemoprophylaxis and placebo for recurrent acute otitis media in children aged under 2 years: Randomised controlled trial. British Medical Journal 2004 328:7438 (487-490)
- Liston TE, Harbison R. Sulfisoxazole chemoprophylaxis and recurrent otitis media. West J Med. 1984 Jan;140(1):47-9
- Persico M., Podoshin L., Fradis M., et al. Recurrent acute otitis media - Prophylactic penicillin treatment: A prospective study. Part I. International Journal of Pediatric Otorhinolaryngology 1985 10:1 (37-46).
- Prellner K, Foglé-Hansson M, Jørgensen F, Kalm O, Kamme C. Prevention of recurrent acute otitis media in otitis-prone children by intermittent prophylaxis with penicillin. Acta Otolaryngol. 1994 Mar;114(2):182-7. Schuller_et al.. 1983
- Schwartz RH, Puglise J, Rodriguez WJ. Sulphamethoxazole prophylaxis in the otitis-prone child. Arch Dis Child. 1982 Aug;57(8):590-3.
- Schuller_DE. Prophylaxis of otitis media in asthmatic children. Pediatric Infectious Disease 1983;2(4):280-3.
- Sih T, Moura R, Caldas S, Schwartz B. Prophylaxis for recurrent acute otitis media: a Brazilian study. Int J Pediatr Otorhinolaryngol. 1993 Jan;25(1-3):19-24.

Excluded studies

AOM Questions

- Ghosh A., Chatterjee S. Comparison of efficacy and safety of cefpodoxime and amoxicillin-clavulanate potassium in paediatric acute otitis media in children below two years: A prospective longitudinal study. Journal of Clinical and Diagnostic Research 2017 11:6 (FC01-FC04)
- Hay AD, Moore MV, Taylor J, Turner N, Noble S, Cabral C, Horwood J, Prasad V, Curtis K, Delaney B, Damoiseaux R, Domínguez J, Tapuria A, Harris S, Little P, Lovering A, Morris R, Rowley K, Sadoo A, Schilder A, Venekamp R, Wilkes S, Curcin V. Immediate oral versus immediate topical versus delayed oral antibiotics for children with acute otitis media with discharge: the REST three-arm non-inferiority electronic platform-supported RCT. Health Technol Assess. 2021 Nov;25(67):1-76.
- Hoberman A, Paradise JL, Rockette HE, Jeong JH, Kearney DH, Bhatnagar S, Shope TR, Muñiz G, Martin JM, Kurs-Lasky M, Haralam M, Pope MA, Nagg JP, Zhao W, Miah MK, Beumer J, Venkataramanan R, Shaikh N. Reduced-Concentration Clavulanate for Young

Children with Acute Otitis Media. Antimicrob Agents Chemother. 2017 Jun 27;61(7):e00238-17

- Hullegie S., Venekamp R.P., Van Dongen T.M.A., Mulder S., Van Schaik W., De Wit G.A., Hay A.D., Little P., Moore M.V., Sanders E.A.M., Bonten M.J.M., Bogaert D., Schilder A.G.M., Damoiseaux R.A.M.J. Topical or oral antibiotics for children with acute otitis media presenting with ear discharge: Study protocol of a randomised controlled non-inferiority trial. BMJ Open 2021 11:12
- Kono M, Fukushima K, Kamide Y, Kunimoto M, Matsubara S, Sawada S, Shintani T, Togawa A, Uchizono A, Uno Y, Yamanaka N, Hotomi M. Features predicting treatment failure in pediatric acute otitis media. J Infect Chemother. 2021 Jan;27(1):19-25
- Oliveira A.G., Marques J.S., Costa I.S., Reis S., Antunes J., Baptista C. Acute otitis media in children, diagnosis and management. Archives of Disease in Childhood 2021 106:SUPPL 2 (A29-)
- Ruohola A, Laine MK, Tähtinen PA. Effect of Antimicrobial Treatment on the Resolution of Middle-Ear Effusion After Acute Otitis Media. J Pediatric Infect Dis Soc. 2018 Feb 19;7(1):64-70.
- Tähtinen PA, Laine MK, Ruohola A. Prognostic Factors for Treatment Failure in Acute Otitis Media. Pediatrics. 2017 Sep;140(3):e20170072.
- Uitti JM, Tähtinen PA, Laine MK, Ruohola A. Close Follow-up in Children With Acute Otitis Media. Initially Managed Without Antimicrobials. JAMA Pediatr. 2016 Nov 1;170(11):1107-1108.

Question 9 - RAOM

- Appelman CL, Claessen JQ, Touw-Otten FW, Hordijk GJ, de Melker RA. Co-amoxiclav in recurrent acute otitis media: placebo controlled study. BMJ. 1991 Dec 7;303(6815):1450-2. doi: 10.1136/bmj.303.6815.1450.
- Arguedas A., Sher L., Lopez E., Sáez-Llorens X., Hamed K., Skuba K., Pierce P.F.Open label, multicenter study of gatifloxacin treatment of recurrent otitis media and acute otitis media treatment failure. Pediatric Infectious Disease Journal 2003 22:11 (949-955)
- Arrieta A, Arguedas A, Fernandez P, Block SL, Emperanza P, Vargas SL, Erhardt WA, de Caprariis PJ, Rothermel CD. High-dose azithromycin versus high-dose amoxicillin-clavulanate for treatment of children with recurrent or persistent acute otitis media. Antimicrob Agents Chemother. 2003 Oct;47(10):3179-86
- Bezáková N, Damoiseaux RA, Hoes AW, Schilder AG, Rovers MM. Recurrence up to 3.5 years after antibiotic treatment of acute otitis media in very young Dutch children: survey of trial participants. BMJ. 2009 Jun 30;338:b2525
- Block S.L., Harrison C.J., Hedrick J., Tyler R., Smith A., Hedrick R. Restricted use of antibiotic prophylaxis for recurrent acute otitis media in the era of penicillin non-susceptible Streptococcus pneumoniae. International Journal of Pediatric Otorhinolaryngology 2001 61:1 (47-60).
- Cárdenas N., Martín V., Arroyo R., López M., Carrera M., Badiola C., Jiménez E., Rodríguez J.M. Prevention of recurrent acute Otitis media in children through the use of lactobacillus salivarius PS7, a target-specific probiotic strain. Nutrients 2018 11:2
- Casselbrant ML, Kaleida PH, Rockette HE, Paradise JL, Bluestone CD, Kurs-Lasky M, Nozza RJ, Wald ER. Efficacy of antimicrobial prophylaxis and of tympanostomy tube insertion for prevention of recurrent acute otitis media: results of a randomized clinical trial. Pediatr Infect Dis J. 1992 Apr;11(4):278-86.
- Damoiseaux RA, Rovers MM, Van Balen FA, Hoes AW, de Melker RA. Long-term prognosis of acute otitis media in infancy: determinants of recurrent acute otitis media and persistent middle ear effusion. Fam Pract. 2006 Feb;23(1):40-5.

- De Diego JI, Prim MP, Alfonso C, Sastre N, Rabanal I, Gavilan J. Comparison of amoxicillin and azithromycin in the prevention of recurrent acute otitis media. Int J Pediatr Otorhinolaryngol. 2001 Apr 6;58(1):47-51
- Ellul D., Mohamad S.H., Miah M.S., Spielmann P., Hussain S.S.M. Recurrent acute otitis media in children: Prospective study of outcome following a six-week course of oral antibiotics. Journal of Laryngology and Otology 2018 132:4 (1-)
- Fauskin G. Acute otitis media in early infancy. Recurrence and prophylaxis. Acta Paediatrica Scandinavica 1991 80:4 (418-422)
- Fliss DM, Krauss M, Gorodischer R, Bearman J, Lieberman A. [Cefaclor and trimethoprim-sulfamethoxazole for recurrent otitis media]. Harefuah. 1989 Dec.1;117(11):361-3
- Foglé-Hansson M, White P, Hermansson A, Prellner K. Short-term penicillin-V prophylaxis did not prevent acute otitis media in infants. Int J Pediatr Otorhinolaryngol. 2001 Jun 7;59(2):119-23
- Gray B. Controlled trial of sulfamethoxazole-trimethoprim for the prevention of recurrent acute otitis media in young children. Current Chemotherapy & Immunotherapy. Proceedings of the 12th International Congress of Chemotherapy. Florence, Italy, 1981.
- Hampton T., Whitehall E., Beasley C., Stevens K., Lowe N., Hogg E., Bhat J., Emerson H., Krishnan M., Sharma S. Recurrent acute otitis media: A survey of current management in England. Journal of Laryngology and Otology 2021 135:10 (855-857).
- Jacobsson S, Fogh A, Larsson P, Lomborg S. Evaluation of amoxicillin clavulanate twice daily versus thrice daily in the treatment of otitis media in children. Danish-Swedish Study Group. Eur J Clin Microbiol Infect Dis. 1993 May;12(5):319-24
- Leach AJ, Morris PS, Mathews JD; Chronic Otitis Media Intervention Trial -One (COMIT1) group. Compared to placebo, long-term antibiotics resolve otitis media with effusion (OME) and prevent acute otitis media with perforation (AOMwiP) in a high-risk population: a randomized controlled trial. BMC Pediatr. 2008 Jun 2;8:23
- Mandel EM, Casselbrant ML, Rockette HE, Bluestone CD, Kurs-Lasky M. Efficacy of antimicrobial prophylaxis for recurrent middle ear effusion. Pediatr Infect Dis J. 1996 Dec;15(12):1074-82.
- Marchisio P, Principi N, Sala E, Lanzoni L, Sorella S, Massimini A. Comparative study of once-weekly azithromycin and once-daily amoxicillin treatments in prevention of recurrent acute otitis media in children. Antimicrob Agents Chemother. 1996 Dec;40(12):2732-6
- Maynard JE, Fleshman JK, Tschopp CF. Otitis media in Alaskan Eskimo children. Prospective evaluation of chemoprophylaxis. JAMA. 1972 Jan 31;219(5):597-9.
- Noel GJ, Blumer JL, Pichichero ME, Hedrick JA, Schwartz RH, Balis DA, Melkote R, Bagchi P, Arguedas A. A randomized comparative study of levofloxacin versus amoxicillin/clavulanate for treatment of infants and young children with recurrent or persistent acute otitis media. Pediatr Infect Dis J. 2008 Jun;27(6):483-9.
- Odio CM, Kusmiesz H, Shelton S, Nelson JD. Comparative treatment trial of augmentin versus cefaclor for acute otitis media with effusion. Pediatrics. 1985 May;75(5):819-26.
- Perrin JM, Charney E, MacWhinney JB Jr, McInerny TK, Miller RL, Nazarian LF. Sulfisoxazole as chemoprophylaxis for recurrent otitis media. A double-blind crossover study in pediatric practice. N Engl J Med. 1974 Sep 26;291(13):664-7
- Principi N, Marchisio P, Massironi E, Grasso RM, Filiberti G. Prophylaxis of recurrent acute otitis media and middle-ear effusion. Comparison of amoxicillin with sulfamethoxazole and trimethoprim. Am J Dis Child. 1989 Dec;143(12):1414-8.
- Roark R, Berman S. Continuous twice daily or once daily amoxicillin prophylaxis compared with placebo for children with recurrent acute otitis media. Pediatr Infect Dis J. 1997 Apr;16(4):376-81.

- Roos K, Larsson P. Efficacy of ceftibuten in 5 versus 10 days treatment of recurrent acute otitis media in children. Int J Pediatr Otorhinolaryngol. 2000. Sep 29;55(2):109-15.
- Sáez-Llorens X, Rodriguez A, Arguedas A, Hamed KA, Yang J, Pierce P, Echols R. Randomized, investigator-blinded, multicenter study of gatifloxacin versus amoxicillin/clavulanate treatment of recurrent and nonresponsive otitis media in children. Pediatr Infect Dis J. 2005 Apr;24(4):293-300
- Salah M., Abdel-Aziz M., Al-Farok A., Jebrini A.Recurrent acute otitis media in infants: Analysis of risk factors. International Journal of Pediatric Otorhinolaryngology 2013 77:10 (1665-1669)
- Sher L, Arguedas A, Husseman M, Pichichero M, Hamed KA, Biswas D, Pierce P, Echols R. Randomized, investigator-blinded, multicenter, comparative study of gatifloxacin versus amoxicillin/clavulanate in recurrent otitis media and acute otitis media treatment failure in children. Pediatr Infect Dis J. 2005 Apr;24(4):301-8
- Te Molder M., De Hoog M.L.A., Uiterwaal C.S.P.M., Van Der Ent C.K., Smit H.A., Schilder A.G.M., Damoiseaux R.A.M.J., Venekamp R.P. Antibiotic treatment for first episode of acute otitis media is not associated with future recurrences. PLoS ONE 2016 11:9
- Teele DW, Klein JO, Word BM, Rosner BA, Starobin S, Earle R Jr, Ertel CS, Fisch G, Michaels R, Heppen R, Strause NP; Greater Boston Otitis Media Study Group. Antimicrobial prophylaxis for infants at risk for recurrent acute otitis media. Vaccine. 2000 Dec 8;19 Suppl 1:S140-3
- Varsano I, Volovitz B, Mimouni F. Sulfisoxazole prophylaxis of middle ear effusion and recurrent acute otitis media. Am J Dis Child. 1985 Jun;139(6):632-5.
