# Supplementary material for: Antibiotic treatment of acute and recurrent otitis media in children: an Italian intersociety Consensus
Source: Ital J Pediatr. 2025 Feb 20;51:50. doi: 10.1186/s13052-025-01894-z (PMC11844117; doi:10.1186/s13052-025-01894-z)
Supplement: Supplementary file 8 — Additional file 8. S8_Italian intersociety consensus_Antibiotics_STRUCTURE AND METHODOLOGY OF THE DOCUMENT.pdf (Itaian Intersociety consensus on antibiotic therapy of respiratory infections in pediatric age). [file 13052_2025_1894_MOESM8_ESM.pdf]

S8. ITALIAN INTERSOCIETY CONSENSUS (SIPPS-SIP-SITIP-FIMP-SIAIP-SIMRI-FIMMG) ON ANTIBIOTIC THERAPY OF RESPIRATORY INFECTIONS IN PEDIATRIC AGE

STRUCTURE AND METHODOLOGY OF THE DOCUMENT

Document Structure

This document aims to give the generalist paediatrician (family paediatrician, outpatient or hospital specialist) a practical and up-to-date tool. The document has been designed and structured according to three possible consultation methods:

1. Summary of the recommendations: The summary of recommendations contain only the final practical indications related to the chapters and questions

2. Pathology sections: the pathology sections deal with the topic *in extenso*. Each chapter includes two parts. First, an introductory narrative part, with updated summaries of the definition of the treated pathology, the clinical framework and the diagnostic management. The second part is developed according to the GRADE methodology related to antibiotic treatment in children with this pathology. We answered the questions based on research, analysis and synthesis of the scientific evidence and the "Evidence to the Decision" (EtD) process that explained and graded the recommendations

This document does not address issues related to children with comorbidities or underlying chronic disease, as specified below in the paragraph "Setting and reference population", management with therapies other than antibiotics and management of complications.

3. Appendix: Boxes, Figures and Tables included in the Appendix allow a quick consultation of the references, the adopted processes, the obtained results, and the recommendations formulation .

Methods

We decided to produce a Consensus Conference (CC) to provide for an analysis of the available evidence on the subject of the judicious use of antibiotic therapy in respiratory tract infections in developmental age. These issues may not have a complete sharing of opinions or conditions of uncertainty. This lack of generality can lead to inhomogeneity of behaviours on a clinical level, and the management aspects of assistance.

The Panel drew up the project and defined the general aims of the document and the specific objectives. We alsoe established the time required for each phase and the coordination activity, the topics, the methods of consultation, the research and the selection of the studies, and the processes defining the Consensus by the Panel.

Working Groups

- Promoting Committee of the CC, which organized and directed the different stages of development of the CC;

- The Scientific and Technical Committee critically analyzed the literature, extracted and tabulated the relevant data, and elaborated the synthesis of the scientific literature. It also supported the formulation of recommendations according to the GRADE method. Finally, it prepared the

questionnaire for voting on the recommendations according to the Delphi method and analyzed the results;

- Multidisciplinary and multi-professional panel or Jury panel (G), which elaborated the clinical questions, discussed the efficacy tests and formulated the recommendations, divided into subgroups by pathology in some work phases ;

- Writing group, which drafted the final text of the CC.

- A group of External Auditors of paediatricians and infectious disease specialists with expertise in the specific pathologies.

The Scientific Technical Committee and the Panel Committee held periodic meetings. The dates of the meetings and all preliminary versions of the document have been recorded.

The multidisciplinary and multi-professional panel includes paediatricians experts in infectious diseases, developmental age, general paediatricians and paediatricians of free choice. The panel also includes experts in allergology, clinical pharmacology, microbiology, epidemiology and research methodology and parent representatives.

The members of the Groups have been indicated by the Scientific Societies of the various disciplines or by the reference Associations.

We used the Delphi method to reach an agreement on the selected topics and the strength of the recommendations[i].

The external auditors did not participate in any phase of the development and drafting of the document, nor they voted for the recommendations.

Consensus Conference Audience

There is a wide group of primary users of the CC including family paediatricians, general paediatricians working in outpatient facilities and outpatient DEAs, paediatricians experts in infectious diseases, general practitioners (GPs) and Continuity of Care doctors.
The group also includes otolaryngology specialists, pulmonologists, immunologists, hygienists, pharmacologists, nurses and pharmacists involved in the management of children with respiratory infections.Parents and caregivers are also users of the document.

Setting and target population

This CC refers to the outpatient setting and emergency room management in the context of both Family Paediatrics and General Practitioners, or to hospitalised patients in the clinical conditions considered in this document, with the exclusions reported below. The CC provides recommendations on the management of children aged one month to 18 years with the following respiratory infectious diseases: pharyngotonsillitis (FT), acute and chronic sinusitis, acute otitis media (OMA) and recurrent acute otitis media (OMAR), community-acquired pneumonia (CAP). Children less than one month of age, with chronic diseases or comorbidities are excluded for the treatment of which we refer to the specific guidelines by pathology. The chronic diseases and comorbidities include primary or secondary cystic fibrosis and/or CFTR-pathies, primary ciliary dyskinesia, non-cystic fibrosis bronchiectasis, genetic diseases, known malformations of the cardio-respiratory system, neuromuscular diseases and other pre-existing chronic lung diseases, neoplasms, asthma, diabetes mellitus.

Formulation of definitions and questions

The definition of the treated pathology has been reported at the beginning of each chapter according to the available literature.

The questions and the outcomes shared and discussed within the panel have been identified. The questions were formulated by the methodology group using the "PICO" model (Patient/Population [P]; Intervention/Indicator [I]; Comparator/Control [C]; Outcome [O]) and developed according to the GRADE method.

The panel identified the outcomes a priori and then ranked (from 1 to 9) them in terms of importance in the decision-making process

Only outcomes categorized as “critical” or “important” were considered for the literature review and the formulation of the recommendation.

In particular, the outcomes related to antibiotic therapy of respiratory infections in children are the only ones of concern, while those related to diagnostic management and therapies can be excluded. The treatment of the specific complications of the pathologies of concern have been neglected as well.

The subsets of the members' panel developed the clinical questions of the different pathologies.

5.7. Research of Scientific Evidence

We based the bibliographic search on the principle of hierarchical selection.

Firstly, summaries of evidence, Systematic Reviews, were sought.

We have also taken into account the most valid evidence-based LGs

The research was then completed, according to the principle of theoretical saturation, with the Primary Studies published after those included in the RS and with those considered relevant.

5.7.1. General Inclusion Criteria:

- Time limit of the search:

  for RS: last ten years

  for primary studies: from the date of closure of the bibliography of the RS included or, failing that, not older than ten years. We included the studies considered valid and relevant, found by manual research or indicated by experts regardless of the date of publication

- Publication language:

  English, Italian. We also evaluated studies published in other languages if known to the authors, found by manual research or indicated by experts and considered valid and relevant.

- Population :

paediatric and adolescent patients, older than one month, without comorbidities or risk factors, suffering from the following respiratory infectious diseases: pharyngotonsillitis (FT), acute and chronic sinusitis, acute otitis media (OMA) and recurrent acute otitis media (OMAR), community-acquired pneumonia (CAP).

- Type of studies:

Systematic Review, Meta-Analysis, Randomized Controlled Trial, Multicentre Study, Observational Study, Cohort Study, Longitudinal Study

- Relevance to the clinical question
- Methodological validity, evaluated based on the minimum criteria described in the chapter "Analysis of scientific evidence".

The research strategy was discussed and agreed upon among the methodologists.

At least two authors carried out the research, evaluation of scientific evidence and data extraction. In case of disagreement, we decided after a discussion among the methodologists.

Research of Systematic Reviews and Primary Studies

1. RS databases: *Cochrane Library*, CDSR – *Cochrane Database of Systematic Reviews,* DARE – *Database of Abstract of Review of Effects In Cochrane Reviews, Other Reviews, Trials*

2. PubMed *http:// www.ncbi.nlm.nih.gov/pubmed*

3. EMBASE *https://www.embase.com*

4. SCOPUS *https://www.scopus.com/*

5. Manual Search

6. Bibliography from experts

Keywords for Population, Intervention/Exposure Factor, Outcome and Search Strings

for each question are reported in the Supplementary files.

Analysis of scientific evidence

We used validated checklists and criteria to perform the evidence analysis and evaluation.

We used the validated AGREE II tool[ii]]

For the evaluation of other consensus documents, we used the criteria defined by the SNLG[iii]

- Relevance of the topic
- Publication date < 3 years
- Multidisciplinary and multi-professional composition of the panel of experts
- Clear and detailed description of the methodology adopted and in line with the standards adopted by CNEC to assess the quality of scientific evidence

We used the validated tool AMSTAR 2 (*Assessment of Multiple Systematic Reviews*)[iv].

Minimum score: overall judgement of high, moderate and low methodological quality.

We assessed any *bias* of the RCTs with the validated tool of the *Cochrane Collaboration* called "*Assessment of Risk of Bias*".[v]

We used the Cochrane ROBINS-I tool for assessing the non-randomized controlled intervention trials, [vi]

We used *Newcastle Ottawa Scales* to assess the observational studies: cohort, case-control, and cross-sectional.[vii]

We took the biases and the confounding factors into account to assess the quality of the studies.

Minimum validity criterion: absence of bias.

GRADE Method[viii,ix,x]

Grading the quality of the evidence

**Quality level**	**Meaning**	**Consequence**
High	High degree of confidence in the results	It is unlikely that further studies will change confidence in effect estimation
Moderate	A fair degree of confidence in the results	Further studies may likely confirm or change confidence in effect estimation
Low	The results are hardly credible	More research is needed to obtain reliable estimates of the positive and negative effects of the intervention
Very low	The data examined is unreliable	You cannot rely on available effect estimates

Criteria for *upgrading* or *downgrading* the quality assessment (high, moderate, low, very low) of the tests

<table>
<tr><td colspan="2">Type of Tests
Randomized Controlled Trial = High
Observational study = low
Any other information = very low</td></tr>
<tr><td>At. Decrease of the category Attribution (e.g. from "high" to "moderate")</td><td>1. Severe (-1 level) or very severe (-2 levels) limitations in the quality of study conduct
2. Inconsistency in results between different studies on the same question (-1 or -2 levels)
3. Some (-1 level) or important (-2 level) uncertainties about the direct transferability of results (directness)
4. Inaccuracy or insufficient data (sparse data) (-1 or -2 levels)
5. Possibility of selective publication and reporting bias (-1 or -2 levels)</td></tr>
</table>

**B.** Increase of the category Attribution (e.g. from "low" to "moderate")	1. Strong intervention-outcome association, i.e. with relative risk >2 (<0.5), based on concordant evidence from two or more observational studies, without any plausible confounding factor (+1 level)<br>2. Very strong intervention-outcome association, i.e. with relative risk >5 (<0.2) (+2 levels)<br>3. Presence of a dose-response gradient (+1 level)<br>4. All possible confounding factors that could have altered the effect estimates would have reduced the observed effect (+1 level)

Outline of recommendations

<u>Recommendations Strength Assessments</u>

Strong recommendation *for* action

Weak recommendation *in favour* of action

Weak recommendation *against* intervention

Strong Recommendation *Against* Intervention

Determinants of the strength of the recommendation

1) *Balance between desirable and undesirable effects*

If the difference between the magnitude of the desired and undesirable outcomes is large, the recommendation is strong (for or against the intervention). If the difference is small, the recommendation is weak.

2) *Overall quality of evidence for the outcomes considered*

The higher the quality of evidence for the outcomes considered, the stronger a recommendation.

3) *Values and preferences*

The more the assigned values and preferences diverge or the greater the uncertainty is, the greater the chance that the recommendation will be weak.

4) Costs (resource allocation)

The higher the costs of an intervention (i.e. the more resources consumed), the less likely it is to consider a strong recommendation.

Note. The formulation of the recommendations has been the subject of great attention and in-depth discussions, both on the substantive and formal aspects. Even in cases where good or moderate quality scientific evidence was not available, we noted that the authors tried to formulate shared recommendations, appropriate to the severity of the disease considered, taking into account the risks and benefits.That is, it is not unusual, nor wrong, to formulate strong recommendations based on low-quality evidence or even based on expert opinion.

Approval of recommendations

We used the Delphi method with a blinded questionnaire

to vote on the recommendations.

We prepared five possible answers: *strongly agree, agree, neither agree nor disagree, disagree, strongly disagree.*

There are no unambiguous criteria for approving recommendations.

Informally, we considered that in several documents of good methodological quality, the panel approved the recommendations with a percentage of agreement equal to 70-75% *("strongly agree", "agree"*).

We requested the reasons in case of *"neither agree nor disagree", "disagree", and "strongly disagree" answers*.

In any case, all the comments of the disagreeing votes were recorded and carefully considered, both on the content of the recommendation and on the formal correctness and clarity of exposition.

GRADE-ADOLOPMENT

The GRADE-ADOLOPMENT method is an evolution of the GRADE method that allows you to assess whether you can adapt to your context or adopt existing LG recommendations published to answer the PICO1 questions.

In this paper, we evaluated the possibility of adopting the recommendations of some LGs on the therapy of OMA and OMAR.

Presentation, participation of Users

We presented a non-final version of this CC to the Jury of experts, nurses' and parents' associations. After in-depth discussion, the comments and collected observations were incorporated into the document when considered appropriate.

The Jury then defined the conclusions, and the writing committee drafted the preliminary consensus document.

The document was internally reviewed and approved by all panel members.

Four external reviewers assessed the document.

The authors approved the final draft in October 2023

Software

We used the RevMan 5.4.1 software[1], Nordic Cochrane Centre, The Cochrane Collaboration, 2014, to evaluate the methodological quality of the RCTs, the meta-analyses and related figures.

We used the GRADEpro GDT software, developed by the GRADE Working Group, for the overall quality of the evidence and the related tables.

Update

We will update the document after three years or in case of publication of new evidence that leads to changes to the recommendations.

Implementation

We will present the document at scientific meetings, and courses, as well as paediatric *forums and mailing lists*. We will give widespread information to family paediatricians, general paediatricians working in outpatient facilities and outpatient DEAs, paediatricians infectious disease specialists, general practitioners (GPs) and Continuity of Care doctors.

Financing

The SIPPS covered the costs for the document's drafting and publication (panel meetings, editing, printing and distribution). All the authors worked free of charge.

Conflict of interest

Each of the members of the working groups signed a declaration on possible conflicts of interest (CI) in the preliminary stages of the project and at the end of the project.

Management of any CIs:

- the members of the methodology team and the external auditors did not have CI;
- the authors with any ICs did not participate, therefore, in the systematic review of the evidence, but participated in all the other phases of implementations, contributing as according to their competence;
- the methodology group and the authors without ICs checked the correctness and consistency of each part of the document and, in particular, of the recommendations;
- each author could vote, express and justify any disagreement anonymously;
- we discussed the results of the votes and the reasons for any disagreements collectively towards the final version of the conclusions and recommendations.

---

[i] Boulkedid R, Abdoul H, Loustau M, et al. Using and Reporting the Delphi Method for Selecting Healthcare Quality Indicators: A Systematic Review. PLoS One. 2011;6:e20476

[ii] Brouwers M, Kho ME, Browman GP, et al. for the AGREE Next Steps Consortium. AGREE II: Advancing guideline development, reporting and evaluation in healthcare. Can Med Assoc J. 2010. Available online July 5, 2010

[iii] SNLG. Good clinical-care practices. Available in https://snlg.iss.it/?cat=4 (last accessed 24-07-2021)

[iv] Shea BJ, Reeves BC, Wells G, et al. AMSTAR 2: a critical appraisal tool for systematic reviews that include randomised or non-randomised studies of healthcare interventions, or both. BMJ. 2017; 358:J4008

[v]Higgins, J.P.T.; Thomas, J.; Chandler, J.; Cumpston, M.; Li, T.; Page, M.J.; Welch, V.A. (Eds.). Cochrane Handbook for Systematic Reviews of Interventions Version 6.2 (Updated February 2021); Cochrane: 2021. Available online: www.training.cochrane.org/handbook (accessed on 15 July 2022).

[vi]Sterne, J.A.C.; Hernán, M.A.; Reeves, B.C.; Savović, J.; Berkman, N.D.; Viswanathan, M.; Henry, D.; Altman, D.G.; Ansari, M.T.; Boutron, I.; et al. ROBINS-I: A tool for assessing risk of bias in non-randomized studies of interventions. BMJ 2016, 355, i4919e

[vii]Wells GA, Shea B, O'Connell D, et al. The Newcastle-Ottawa Scale (NOS) for assessing the quality of nonrandomized studies in meta-analyses, 2012. Available at: http://wwwohrica/programs/clinical_epidemiology/oxfordasp (last accessed 24-07-2021)

[viii] Schünemann HJ, Oxman AD, Brozek J, et al. GRADE Working Group. Grading quality of evidence and strength of recommendations for diagnostic tests and strategies. BMJ. 2008; 336:1106-10

[ix] Guyatt GH, Oxman AD, Kunz R, et al. GRADE Working Group. Going from evidence to recommendations. BMJ. 2008;336:1049-51

[x] Guyatt GH, Oxman AD, Kunz R, et al. GRADE working group. Incorporating considerations of resource use into grading recommendations. BMJ. 2008;336:1170-73
